# TRIM37 recognizes a bipartite degron to ubiquitinate centrosome substrates

**DOI:** 10.1083/jcb.202608031

**Published:** 2026-11-10

**Authors:** Weronika E. Stachera, Judith Tafur, Kan Yaguchi, Nicole E. Familiari, Manolo U. Rios, Jeffrey B. Woodruff

**Affiliations:** 1 Department of Cell Biology, UT Southwestern Medical Center, Dallas, TX, USA

## Abstract

Dysregulation of the E3 ubiquitin ligase TRIM37 is associated with tumor formation and Mulibrey nanism, a recessive developmental syndrome. TRIM37 regulates steady-state levels of centrosome proteins and limits their ectopic assembly, but how it recognizes and ubiquitinates its substrates is poorly understood. We found that TRIM37 directly ubiquitinates the centrosome-forming protein Cep192 at 7 lysines clustered near its C terminus. TRIM37 binds Cep192 at a C-terminal intrinsically disordered region followed by an ASH domain (IDR+ASH8). Mutation of the 7 lysines or the IDR+ASH8 domain increased Cep192 levels and stability in cells, indicating loss of TRIM37-based regulation. Fusing IDR+ASH8 to unrelated proteins was sufficient to enable their degradation via TRIM37. Biochemical assays revealed that IDR+ASH8 is primarily monomeric and binds TRIM37 via two separate coiled-coil motifs with mid-nanomolar affinity. We propose that the IDR+ASH8 motif is a bipartite degron for TRIM37, enabling it to target centrosome proteins and adjust their steady-state levels.

## Teaser

Stachera et al. reveal that the E3 ligase TRIM37 recognizes a C-terminal domain, called IDR+ASH8, on the centrosome scaffold protein Cep192. The IDR+ASH8 motif is sufficient to transform EB1 and GFP into TRIM37 substrates.

## Introduction

Centrosomes are major microtubule-organizing centers in mitotic cells and are essential for accurate chromosome segregation. These organelles contain two barrel-shaped centrioles surrounded by a supramolecular mass of protein called pericentriolar material (PCM) (Woodruff et al., 2014). To safeguard genome stability, cells must tightly control not only centriole duplication but also the abundance and organization of the PCM (Godinho and Pellman, 2014; Lerit and Poulton, 2016; Watanabe et al., 2019). Excess PCM can impair spindle assembly, promote aneuploidy, and is positively associated with tumor grade (Lingle et al., 1998; Lingle et al., 2002; Xie et al., 2022). Although PCM composition and architecture are well understood, mechanisms that control the levels of core PCM scaffolding proteins remain poorly defined.

One mechanism for controlling PCM protein abundance is proteasome-mediated degradation. The E3 ubiquitin ligase TRIM37 influences the stability of several PCM components, including Cep192, a conserved scaffolding protein required for PCM assembly (Balestra et al., 2021; Meitinger et al., 2020; Yeow et al., 2020). TRIM37 overexpression markedly reduces cellular Cep192 levels; in the absence of centrioles, loss of TRIM37 leads to the accumulation of multiple cytoplasmic foci containing Cep192 (Meitinger et al., 2020). Despite the literature demonstrating a functional dependency between the two proteins, the evidence confirming that TRIM37 binds to Cep192 directly is still missing. Furthermore, the molecular mechanism underlying Cep192 recognition and ubiquitination by E3 ligases remains unknown.

Understanding this pathway is particularly important given the strong disease relevance of TRIM37. The genomic locus harboring *TRIM37* is amplified in several cancers—including neuroblastoma, breast, liver, and kidney tumors (Bhatnagar et al., 2014; Jiang et al., 2015; Meitinger et al., 2020; Miao et al., 2021)—and elevated TRIM37 levels can contribute to chemoresistance (Chen et al., 2020; Nishibeppu et al., 2021; Przanowski et al., 2020; Tan et al., 2021; Yeow et al., 2020). Several studies have proposed that excessive TRIM37 activity destabilizes PCM proteins, thereby compromising mitotic fidelity, which could contribute to cancer progression (Balestra et al., 2021; Meitinger et al., 2020; Yeow et al., 2020; Zhou et al., 2024). Conversely, loss-of-function mutations in *TRIM37* are the only known cause of Mulibrey nanism, an autosomal recessive growth disorder (Avela et al., 2000).

**Figure S1.**
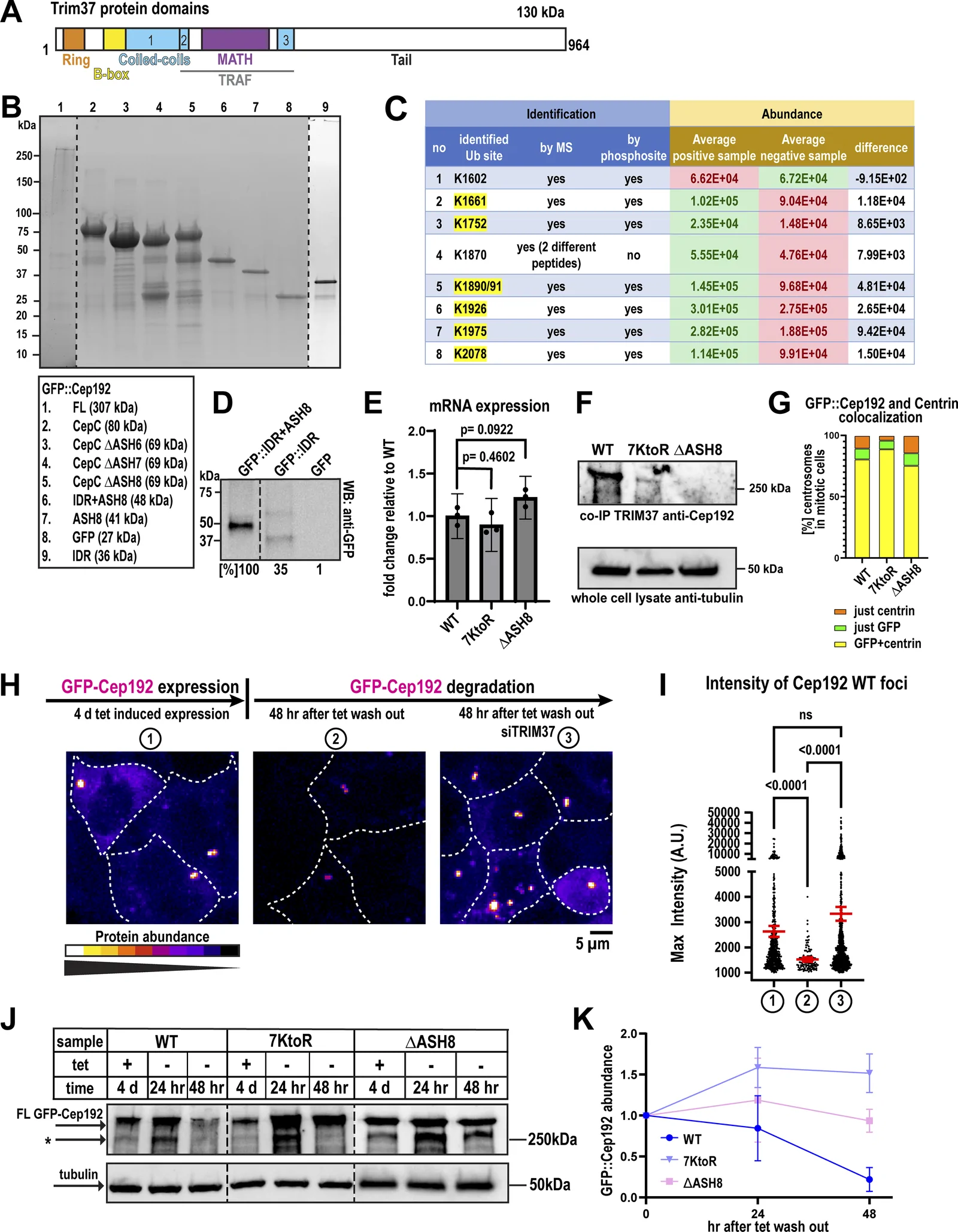
**Identification of ubiquitinated residues and the TRIM37-binding site on Cep192. (A)** Structured and conserved domains of TRIM37. **(B)** SDS-PAGE gel showing purified full-length Cep192 and fragments. **(C)** List of ubiquitinated lysines identified by MS and reported in PhosphoSitePlus (https://www.phosphosite.org/). **(D)** Western blot showing binding efficiency of Cep192 constructs to TRIM37. Constructs were purified from *Escherichia coli* and then pulled down using beads affixed to anti-TRIM37 antibody. Percentages represent relative binding capacity normalized to minimal TRIM37 binding region IDR+ASH8. **(E)** qPCR analysis of construct expression. Mean ± 95% CI; three biological replicates; P values from one-way ANOVA, followed by post hoc Dunnett’s multiple comparison. **(F)** Western blot showing binding efficiency of Cep192 constructs to TRIM37. Constructs were expressed in DLD-1 cells, followed by immunoprecipitation using beads coated with an anti-TRIM37 antibody. Tubulin from the total cell lysate (input) serves as a loading control prior to the pull-down. **(G)** Analysis of GFP-Cep192 and Centrobin co-localization (*n* ≥ 100 centrosomes, three biological replicates). **(H)** Representative images of degradation efficiency of GFP-Cep192(WT) with and without TRI37 knockdown (siTRIM37). **(I)** Quantification of maximum fluorescence intensity of Cep192 foci (mean ± 95% CI; *n* > 114 foci, three biological replicates) during expression and degradation phases. P values from Kruskal–Wallis’s test, followed by post hoc Dunn’s multiple comparison. **(J)** Representative western blot showing construct abundance (anti-GFP) during a degradation assay (top panel). The top band represents full-length GFP-Cep192. The asterisk represents a cross-reactive, lower molecular weight species. Tubulin was used as a loading control (bottom panel). **(K)** Quantification of Western blots showing construct abundance during the degradation assay (mean ± SEM; *n* = 3). Data are normalized to construct levels at 96 h of expression. Source data are available for this figure: SourceData FS1.

Structurally, TRIM37 contains a disordered C-terminal tail preceded by three conserved modules at its N terminus: (1) a RING domain required for E2 recruitment and ubiquitin transfer, (2) a B-box domain that mediates higher order assembly, and (3) a TRAF domain, which contains a folded MATH domain flanked by two coiled-coil domains, important for dimerization and recognition of pathological Centrobin condensates (Bellaart et al., 2025; Wang et al., 2017; Yeow et al., 2025) (Fig. S1 A). While TRIM37-mediated ubiquitination of Centrobin aggregates has been linked to multimerization-dependent activation, this mechanism appears specialized for targeting pathogenic condensates rather than endogenous PCM substrates. It is unclear if TRIM37 identifies and regulates physiological PCM scaffold proteins through the same mechanism.

Here, we identified Cep192 as a direct target of TRIM37 and show that TRIM37 binds a composite region that includes both an intrinsically disordered region (IDR) and a structured β barrel domain (IDR+ASH8). We mapped seven lysine residues that TRIM37 ubiquitinates to promote Cep192 degradation and demonstrate that the IDR+ASH8 module functions as a transferable degron capable of conferring TRIM37-dependent degradation to unrelated proteins.

## Results

### TRIM37 directly ubiquitinates 7 lysines on the C terminus of Cep192

**Figure 1.**
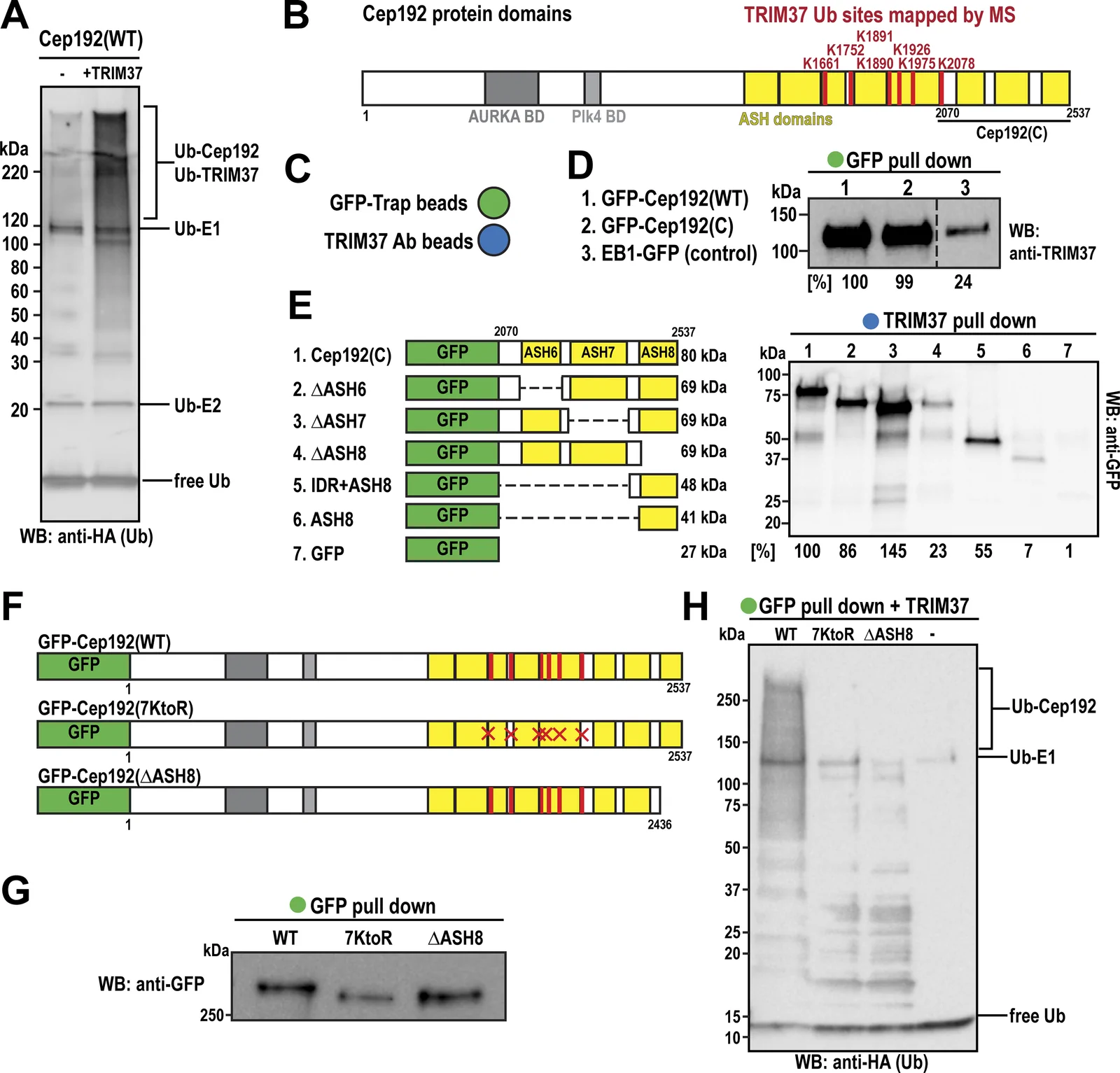
**TRIM37 directly binds the IDR+ASH8 domain of Cep192 and ubiquitinates it on seven specific residues. (A)** Western blot against HA-tagged ubiquitin from reconstituted ubiquitination reactions containing 10 µM HA-ubiquitin, 100 nM E1 UBE1, 100 nM E2 UbcH5b/UBE2D2, 1 µM Cep192, and either 0 µM (control) or 1 µM TRIM37. Representative of three replicates. **(B)** Schematic of Cep192 domains with ubiquitinated residues identified by MS. **(C)** Graphic representation of beads used for immunoprecipitation assays. Green: GFP-Trap magnetic particles; blue: beads conjugated to TRIM37 antibody. **(D)** Western blot showing TRIM37 binding to Cep192 constructs. Percentages represent relative binding capacity normalized to full-length Cep192. **(E)** Anti-GFP western blot showing Cep192 fragments that bind TRIM37. Percentages represent binding capacity normalized to the Cep192 C-terminal fragment. Representative of three replicates. **(F)** Cep192 constructs expressed in DLD-1 cells. **(G)** Western blot against GFP showing pull-down of Cep192 constructs isolated from DLD-1 cells to be used in subsequent assays. **(H)** Western blot against HA-tagged ubiquitin from reconstituted ubiquitination reactions using the Cep192 constructs from G. Reactions also contained 25 nM TRIM37 and 0.5 µM HA-Ubiquitin. Representative of three replicates. Source data are available for this figure: SourceData F1.

While previous studies have shown that TRIM37 associates with Cep192 and regulates its cellular levels (Meitinger et al., 2020; Tkach et al., 2022; Yeow et al., 2020), there is no clear evidence that TRIM37 directly targets Cep192 for ubiquitination. Thus, we established an *in vitro* ubiquitination assay. We purified full-length Cep192 (MW = 307 kDa) from baculovirus-infected insect cells (Fig. S1 B) and combined it with E1 (UBE1) and E2 (UBCH5B) enzymes, ATP, MgCl_2_, and HA-tagged ubiquitin. We then incubated the reactions at 37°C for 1 h, either in the presence or absence of purified TRIM37 (Fig. 1 A). Western blotting revealed an increased ubiquitination signal on high molecular weight proteins in the reaction containing TRIM37 (Fig. 1 A).

To identify specific ubiquitination sites on Cep192, we used mass spectrometry (MS) to detect di-glycine remnants indicative of lysine ubiquitination. We identified seven such peptides that were significantly more abundant in the presence of TRIM37: K1661, K1752, K1890, K1891, K1926, K1975, and K2078 (Fig. 1 B and Fig. S1 C). Notably, these ubiquitination sites cluster near the C-terminal region of Cep192, previously shown to pull down TRIM37 (Fig. 1 B) (Meitinger et al., 2020). AlphaFold3 predicts that this region contains three β barrel ASH-like domains connected by IDRs (Fig. 1 B). We compared the efficiency of TRIM37 binding between full-length Cep192 and the C terminus fragment (2,070–2,537 aa), both GFP-tagged (Fig. 1 B). EB1-GFP served as a negative control. We incubated each GFP fusion protein with an equal amount of TRIM37 at 37°C for 1 h, then precipitated them using GFP-Trap beads (Fig. 1 C). Minimal TRIM37 was detected in the control, while both full-length and C-terminal Cep192 showed similarly strong binding (Fig. 1, C and D). These results demonstrate that TRIM37 directly binds the Cep192 C terminus and ubiquitinates it on seven lysines.

### TRIM37 recognizes a bipartite motif at the C terminus of Cep192

To define which elements mediate TRIM37 binding, we generated GFP-tagged Cep192 C-terminal truncations lacking individual ASH-like domains (ΔASH6, ΔASH7, and ΔASH8) (Figs. 1 E, S1 B). In pull-down assays with bead-immobilized TRIM37 (Fig. 1 C), only Cep192(ΔASH8), which lacks the final 100 aa’s, failed to bind TRIM37 (Fig. 1 E). Surprisingly, neither ASH8 nor the preceding IDR alone bound to TRIM37 as robustly as the longer IDR+ASH8 fragment, indicating that both elements are required for stable interaction (Figs. 1 E, S1 D). Collectively, these data identify the ASH8 domain plus its preceding IDR (IDR+ASH8) as a recognition site for TRIM37.

To determine whether the seven identified ubiquitination sites and the TRIM37-binding domain are required for Cep192 regulation, we generated human colorectal cells expressing transgenes encoding GFP-tagged WT Cep192(WT), a mutant in which all seven lysines were replaced with arginines (7KtoR), or a mutant lacking ASH8 (ΔASH8) (Figs. 1 F, S1 E). We then lysed the cells, purified the proteins using GFP-Trap beads (Fig. 1, C and G), and subjected them to the *in vitro* ubiquitination assay described in Fig. 1 A. Only Cep192(WT) was ubiquitinated by TRIM37, whereas no ubiquitination was detected for the 7KtoR or ΔASH8 mutants (Fig. 1 H). We also tested whether these Cep192 mutants bind TRIM37. As expected, the mutant lacking the ASH8 domain failed to pull down TRIM37, whereas WT and 7KtoR could, although 7KtoR binding was reduced compared with WT (Fig. S1 F). Thus, mutating the ubiquitin acceptor sites or the docking module both prevent Cep192 ubiquitination by TRIM37.

### TRIM37-mediated ubiquitination controls Cep192 levels in human cells

**Figure 2.**
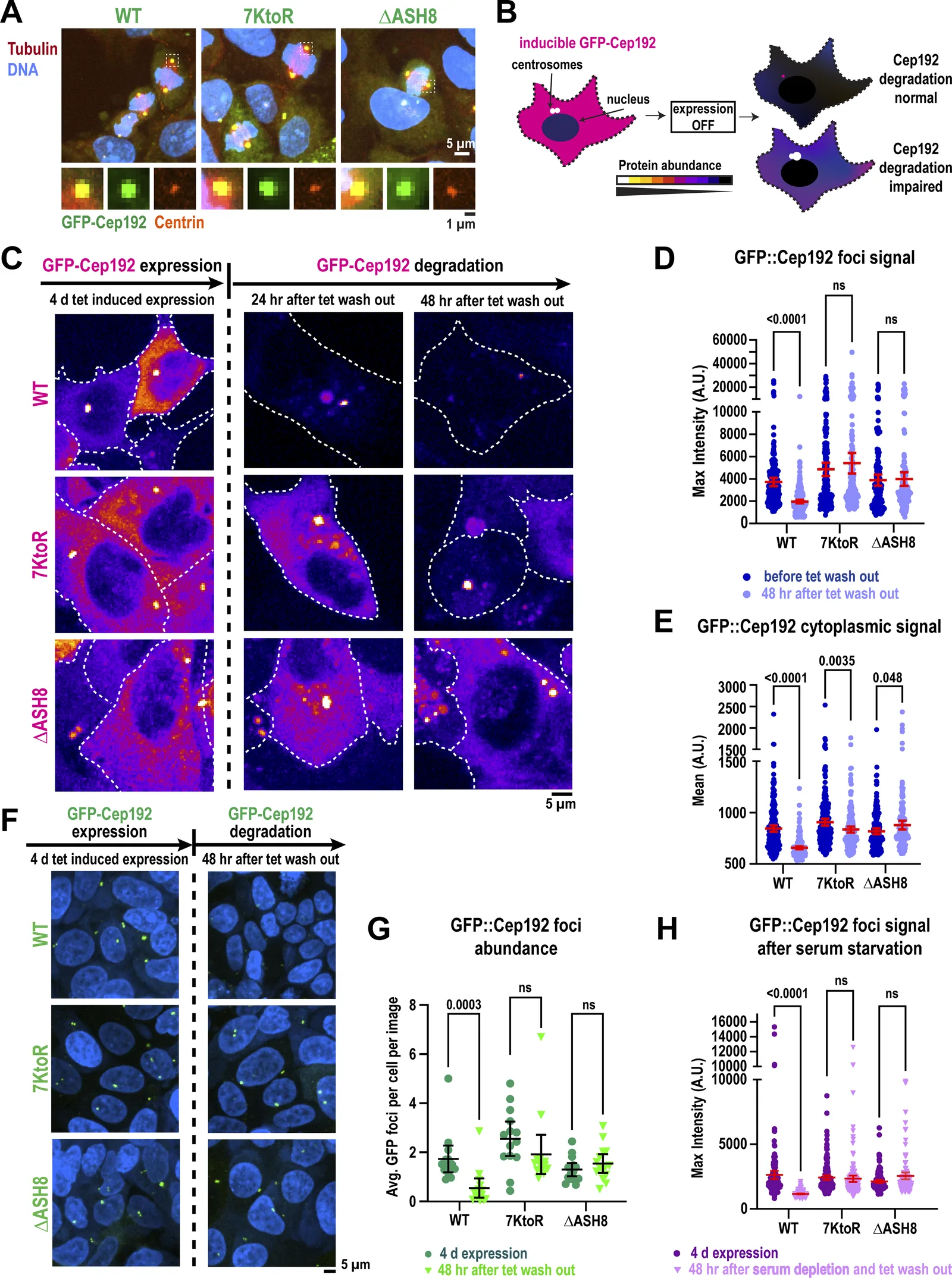
**Ubiquitination-deficient Cep192 mutants show elevated steady-state levels and stability. (A)** Representative immunofluorescence images of cells after 96 h tetracycline-induced expression of Cep192 constructs. GFP-tagged constructs (green), centrin (centrioles; orange), tubulin (dark red), and DNA (blue) are shown. The white box indicates the region magnified below, highlighting centrosomes. **(B)** Schematic of the construct degradation assay. GFP-Cep192 constructs localize to centrosomes and the cytoplasm after tetracycline-induced expression. After tetracycline washout, construct degradation is monitored; lighter color indicates higher protein abundance. **(C)** Representative images showing degradation efficiency of each construct. **(D)** Quantification of maximum fluorescence intensity of Cep192 foci (mean ± 95% CI; *n* > 150, five biological replicates) during expression and degradation (48 h after tet removal) phases. P values from Kruskal–Wallis test, followed by post hoc Dunn’s multiple comparison. **(E)** Quantification of mean fluorescence intensity of Cep192 cytoplasmic signal (mean ± 95% CI; *n* > 150, measured in five biological replicates) during expression and degradation (48 h after tet removal) phases. P values from Kruskal–Wallis test, followed by post hoc Dunn’s multiple comparison. **(F)** Representative images showing degradation efficiency of each construct during IF experiment to calculate the number of foci per cell. Cep192 constructs (green), DNA (blue). **(G)** Quantification of Cep192 foci per cell (mean ± 95% CI; *n* > 14 images per condition, three biological replicates) during expression and degradation (48 h after tet removal) phases. Each dot represents the mean number of foci per nucleus in a single image. P values from Kruskal–Wallis test, followed by post hoc Dunn’s multiple comparison. **(H)** Quantification of maximum fluorescence intensity of Cep192 foci during expression and cell cycle arrest (mean ± 95% CI; *n* = 150, three biological replicates). Cells were analyzed after 96 h of construct expression, followed by tetracycline washout and 48 h of serum starvation to induce cell cycle arrest in G_0_. P values from Kruskal–Wallis test, followed by post hoc Dunn’s multiple comparison.

We next asked whether this modification is necessary for Cep192 degradation in cells. GFP-tagged Cep192(WT), Cep192(7KtoR), and Cep192(ΔASH8) transgenes were induced by tetracycline in the presence of endogenous, untagged Cep192. All GFP-Cep192 constructs accumulated at centrosomes, as indicated by centrin and tubulin staining (Figs. 2 A, S1 G). To examine protein turnover, we removed tetracycline to stop transgene expression and measured GFP-Cep192 signal decay over time (Fig. 2, B–G). 48 h after tetracycline withdrawal, GFP-Cep192(WT) foci and cytoplasmic signal significantly decreased (Fig. 2, C–E). In contrast, foci formed by both Cep192 mutants persisted (Fig. 2, C and D). RT-qPCR confirmed that mRNA expression was similar for all constructs (Fig. S1 E), indicating that any changes in protein abundance are likely due to differences in degradation rates. TRIM37 depletion prevented degradation of GFP-Cep192(WT), mimicking the phenotype of the Cep192 mutants (Fig. S1, H and I). Cytoplasmic levels of Cep192(ΔASH8) slightly increased after tetracycline removal, while Cep192(7KtoR) levels decreased but remained much higher than Cep192(WT) levels (Fig. 2 E). Analysis of fixed cells stained with a DNA marker showed that the number of Cep192(WT) foci per cell significantly decreased following tetracycline removal, while the number of mutant foci remained high (Fig. 2, F and G). In support of the imaging data, western blotting showed that while Cep192(WT) abundance decreased after tetracycline removal, mutant protein levels remained elevated (Fig. S1, J and K). Together, these findings demonstrate that TRIM37-dependent ubiquitination is required for efficient Cep192 degradation and that disruption of this pathway leads to increased Cep192 stability and accumulation.

To verify that the decrease in Cep192 signal is due to degradation and not dilution of proteins caused by multiple cell divisions, we turned off GFP-Cep192 expression and arrested cells in G_0_ using serum starvation. GFP-Cep192(WT) foci still showed a significant reduction in fluorescence intensity, whereas mutant foci remained equally bright (Fig. 2 H). Overall, our findings suggest that TRIM37-mediated ubiquitination of Cep192 regulates its turnover in both mitotic and non-mitotic cells.

### Expression of degradation-resistant Cep192 transgenes causes mitotic arrest and cell death

**Figure 3.**
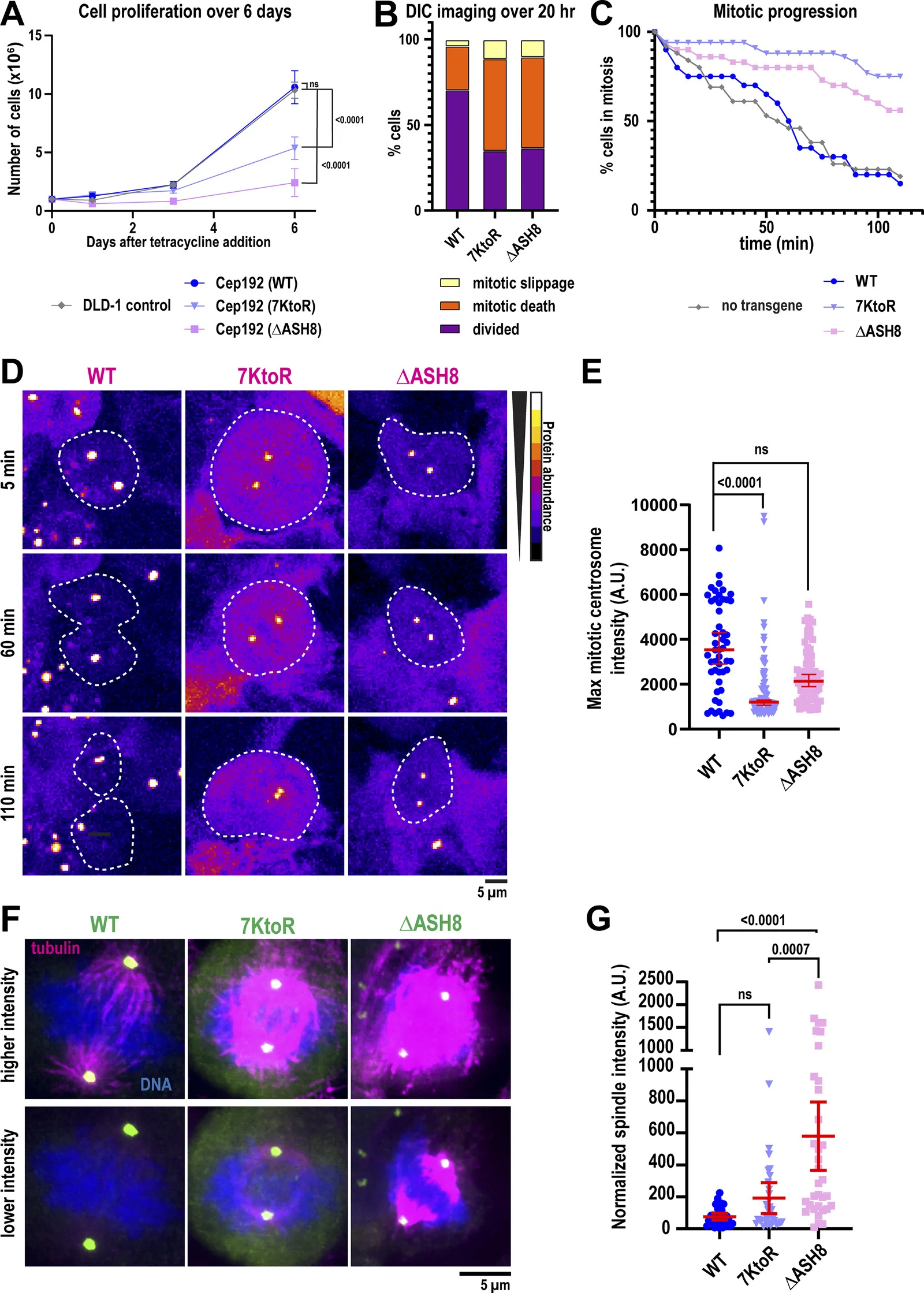
**Cells expressing ubiquitination-deficient Cep192 mutants arrest in mitosis. (A)** Quantification of cell proliferation during expression of Cep192 constructs (mean ± 95% CI; *n* = 6 biological replicates). P values (day 6 viability) from one-way ANOVA followed by post hoc Dunnett’s multiple comparison. **(B)** Quantification of cell fate after 20 h of live imaging (*n* ≥ 54 cells, three biological replicates). **(C)** Time-course quantification showing the cumulative percentage of cells completing mitosis over time with and without indicated Cep192 construct expression (*n* ≥ 20 cells, three biological replicates). **(D)** Representative images of cells expressing Cep192 constructs in mitosis over time. Cells are outlined in white. **(E)** Quantification of maximum fluorescence intensity of GFP-Cep192 constructs at mitotic centrosomes (median ± 95% CI; *n* ≥ 50 centrosomes, four biological replicates). P values from Kruskal–Wallis test, followed by post hoc Dunn’s multiple comparison. **(F)** Immunofluorescence of cells expressing Cep192 constructs highlighting differences in spindle density. GFP-tagged constructs (green), α tubulin (magenta), and DNA (blue) are shown. **(G)** Quantification of α tubulin signal intensity at the spindle during constructs expression (*n* ≥ 27 cells, three biological replicates). P values from Kruskal–Wallis test, followed by post hoc Dunn’s multiple comparison.

We next examined the functional consequences of impaired TRIM37-m8ediated degradation of Cep192. First, we expressed GFP-tagged Cep192 WT, 7KtoR, or ΔASH8 in cells and monitored cell proliferation over 6 days (Fig. 3 A). Cells expressing the 7KtoR mutant exhibited approximately a ∼50% reduction in proliferation relative to WT, whereas ΔASH8 expression resulted in a ∼75% decrease. We then analyzed cell division by differential interference contrast live-cell imaging over 20 h (Fig. 3 B). During this period, most cells expressing Cep192(WT) that entered mitosis—indicated by cell rounding—exited and divided (Fig. 3 B). In contrast, only ∼33% of cells expressing the degradation-deficient Cep192 mutants did so. Many cells instead remained rounded until the end of imaging, and some started blebbing, suggesting prolonged mitotic arrest followed by cell death. Fewer than 10% of cells expressing Cep192 mutants flattened and reentered interphase without dividing, indicating mitotic slippage (Fig. 3 B).

To further investigate the underlying cause of these phenotypes, we used fluorescence live-cell imaging to visualize centrosomes during mitosis. We synchronized cells in late G2 phase, released them, and monitored mitotic progression (Fig. 3, C and D). While DLD-1 cell mitosis averages ∼40 min, it can take up to 2 h (Bloomfield et al., 2020). ∼85% of cells expressing GFP-Cep192(WT) completed mitosis within 2 h, comparable with control cells not expressing any transgene (Fig. 3 C). In contrast, only 25% of 7KtoR and 38% of ΔASH8 cells completed division in that time (Fig. 3, C and D), indicating mitotic arrest.

**Figure S2.**
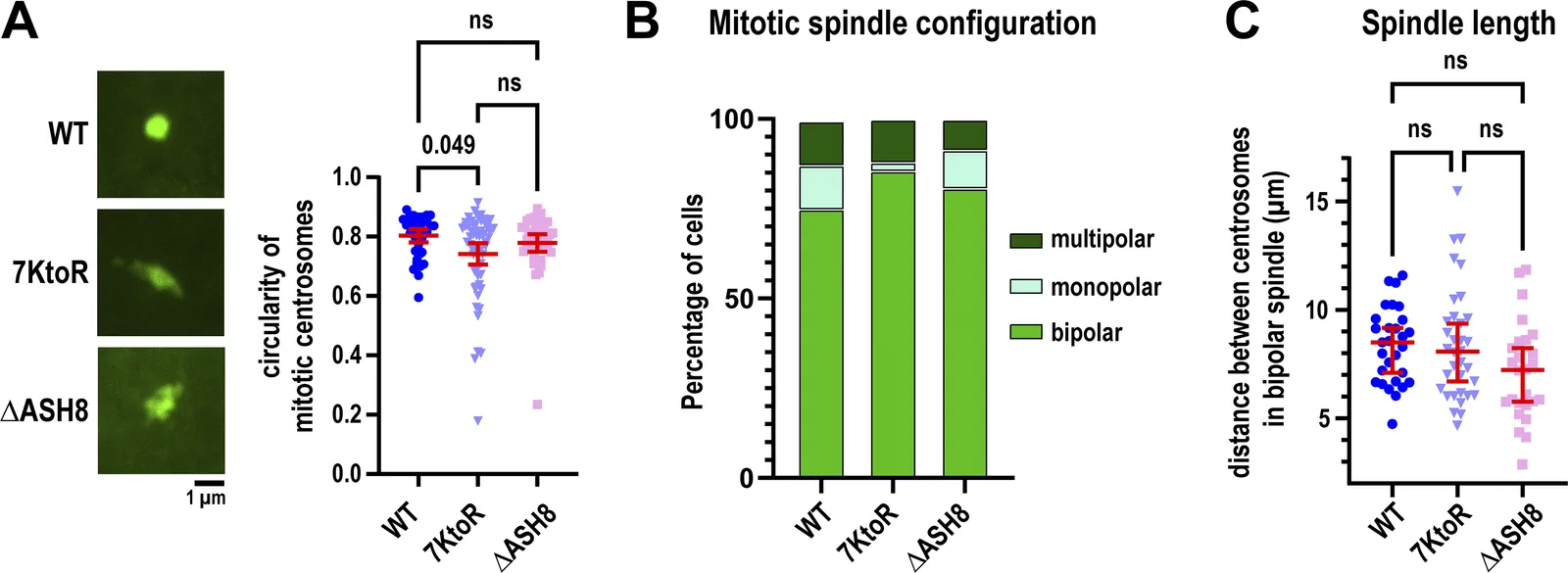
**Analysis of centrosome and spindle shape in cells expressing Cep192 constructs. (A)** Representative examples of centrosomes in transgenic cell lines. Centrosome circularity was quantified (mean ± 95% CI, *n* ≥ 38 centrosomes, three biological replicates). P values were obtained from Kruskal–Wallis test followed by Dunn’s multiple-comparisons test. **(B)** Analysis of mitotic spindle organization in transgenic cell lines, as determined by immunofluorescence (*n* > 30 cells, three biological replicates). **(C)** Quantification of spindle length (median ± 95% CI; *n* >28 cells, three biological replicates). P values from Kruskal–Wallis test followed by Dunn’s multiple-comparisons test.

We sought to determine the cause of these mitosis errors. Surprisingly, both mutants exhibited lower fluorescence intensity at mitotic centrosomes than Cep192(WT) (Fig. 3, D and E), in contrast to our observations in interphase cells (Fig. 2). Thus, although ubiquitin-deficient Cep192 mutants can accumulate to similar levels as WT Cep192 at interphase centrosomes, their recruitment or retention at mitotic centrosomes appears to be impaired. To further examine mitotic centrosome organization, we used super-resolution optical reassignment microscopy (SoRa). Centrosomes appeared elongated and less compact in mutant-expressing cells compared with WT (circularity = 0.803 for WT; 0.741 for 7KtoR; 0.778 for ΔASH8; Fig. S2 A).

Next, we examined spindle organization. Most cells displayed normal bipolar spindles with comparable spindle lengths across all conditions (Fig. S2, B and C). However, cells expressing the ΔASH8 mutant exhibited significantly higher α-tubulin intensity within the mitotic spindle than cells expressing Cep192(WT) (Fig. 3, F and G). Cells expressing the 7KtoR mutant also showed increased spindle α-tubulin intensity, although the difference was not statistically significant (Fig. 3, F and G). Together, these findings suggest that expression of degradation-resistant Cep192 disrupts centrosome organization and spindle architecture, thereby impairing faithful mitotic progression.

### IDR+ASH8 is sufficient to act as a degron

**Figure 4.**
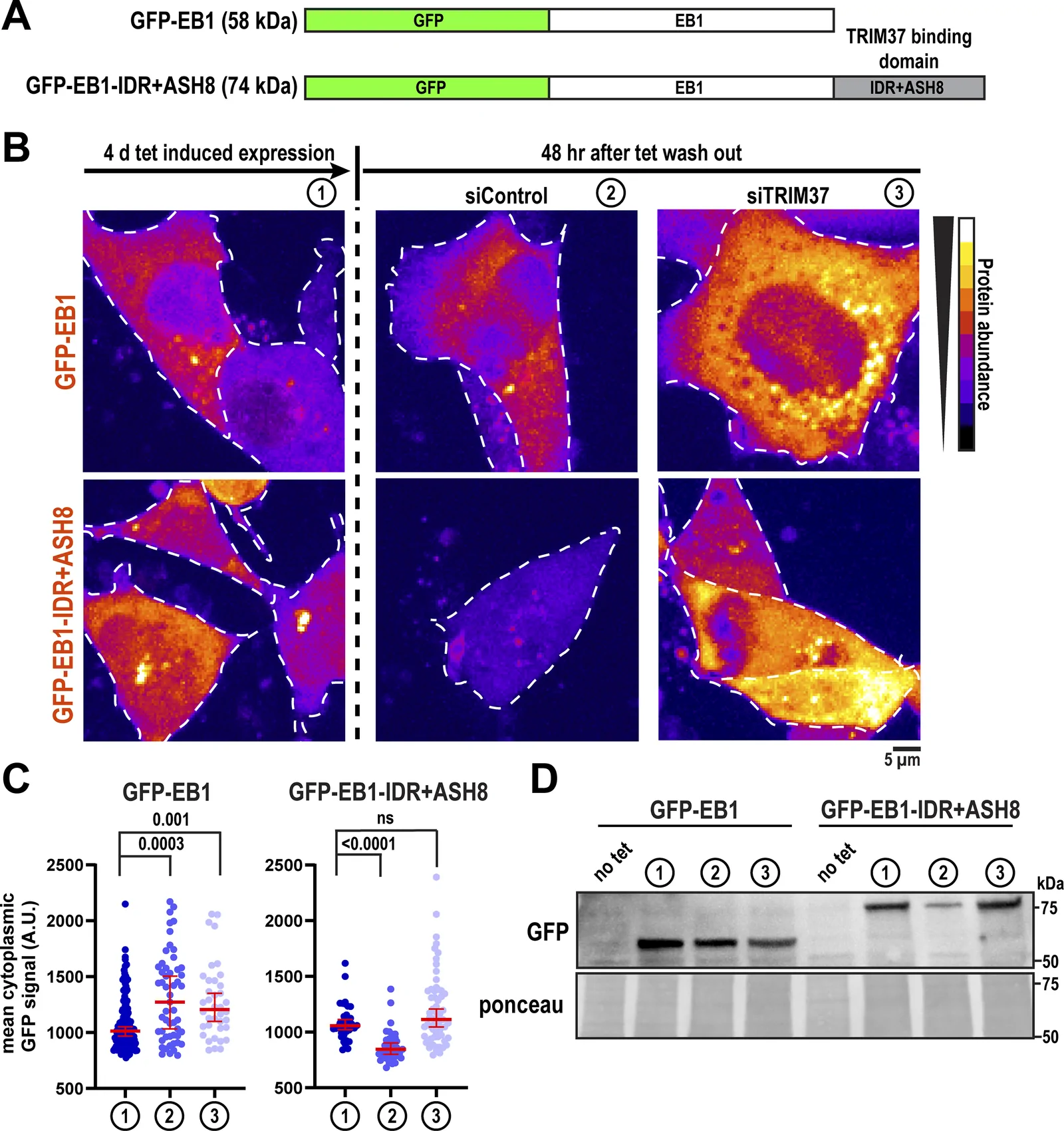
**IDR+ASH8 functions as a transferable degron recognized by TRIM37**. **(A)** EB1 constructs expressed in DLD-1 cells, including one containing the Cep192 IDR+ASH8 domain. **(B)** Representative images showing EB1 constructs: (1) during tetracycline-induced expression, (2) after tetracycline withdrawal with siControl, and (3) after tetracycline withdrawal with siTRIM37. Cells are outlined in white. **(C)** Quantification of cytoplasmic EB1-construct fluorescence under conditions described in B (median ± 95% CI; >35 cells, two biological replicates). P values from Kruskal–Wallis’s test, followed by post hoc Dunn’s multiple comparison. **(D)** Western blot against GFP showing total EB1-construct abundance under the conditions in B. Source data are available for this figure: SourceData F4.

**Figure S3.**
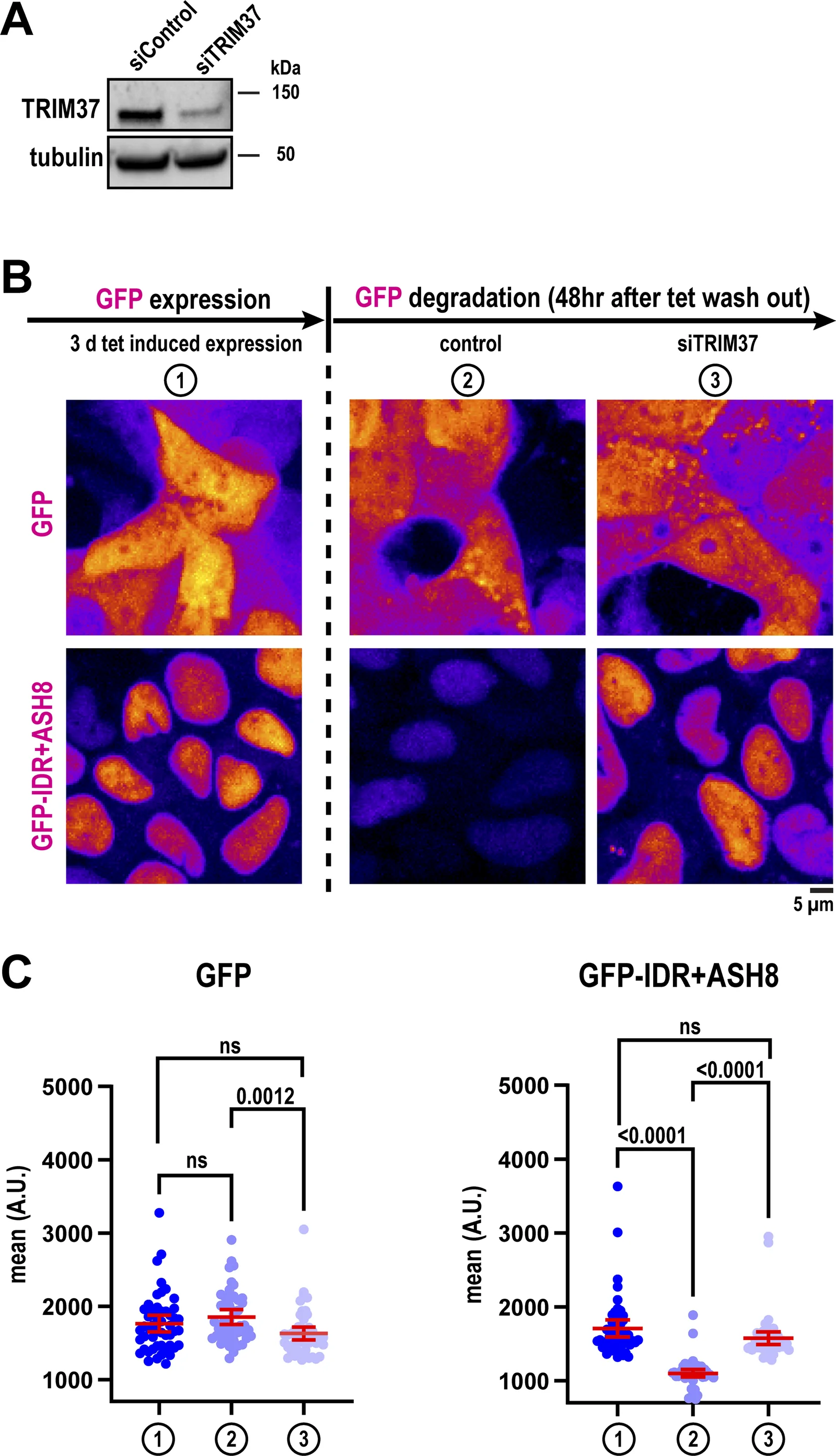
**Testing degron efficiency with a non-centrosomal protein. (A)** Western blot against TRIM37 showing its abundance after siTRIM37 treatment. Tubulin was used as a loading control. **(B)** Representative images showing degradation efficiency of each GFP construct. (1) during tetracycline-induced expression, (2) after tetracycline withdrawal with siControl, and (3) after tetracycline withdrawal with siTRIM37. **(C)** Quantification of mean fluorescence intensity of GFP signal (mean ± 95% CI; *n* > 48 cells, three biological replicates) under conditions described in B. P values from Kruskal–Wallis test, followed by post hoc Dunn’s multiple comparison. Source data are available for this figure: SourceData FS3.

We next examined whether the IDR+ASH8 is sufficient for TRIM37 targeting in cells. The microtubule end-binding protein EB1 localizes to both microtubules and centrosomes (Berrueta et al., 1998; Bu and Su, 2001; Louie et al., 2004); however, it does not interact with TRIM37 in either proximity-based assays (Yeow et al., 2025) or direct *in vitro*–binding experiments (Fig. 1 D). This indicates that EB1 is not a physiological substrate of TRIM37, despite their spatial proximity within the cell. We therefore used EB1 as a neutral scaffold to test whether appending the IDR+ASH8 domain would be sufficient to promote TRIM37-dependent degradation (Fig. 4 A). Using a tetracycline-inducible system in human colorectal cells, we expressed GFP-EB1 or GFP-EB1-IDR+ASH8 (Fig. 4, B–D, condition 1) and then withdrew tetracycline to monitor protein stability in the presence or absence of TRIM37 via siRNA-mediated knockdown (Fig. S3 A). After tetracycline removal, GFP-EB1-IDR+ASH8 levels decreased, whereas GFP-EB1 remained stable (Fig. 4, B–D, condition 2). In contrast, depletion of TRIM37 stabilized GFP-EB1-IDR+ASH8, restoring its levels to those of unmodified EB1 (Fig. 4, B–D, condition 3). These results demonstrate that TRIM37 mediates the degradation of EB1 when fused to the IDR+ASH8 module.

Next, we investigated whether proteins must localize to the centrosome or form higher-order assemblies to undergo TRIM37-mediated degradation. We thus fused the degron motif to monomeric GFP and monitored its degradation following tetracycline withdrawal. GFP alone remained stable after tetracycline removal (Fig. S3, B and C). In contrast, fusion of GFP to IDR+ASH8 caused degradation following tetracycline withdrawal (Fig. S3, B and C). This degradation was abolished upon TRIM37 depletion (Fig. S3, B and C). Thus, the IDR+ASH8 degron is sufficient to convert a non-targeted protein into a TRIM37 substrate. Furthermore, our results suggest that neither centrosome localization nor multimer formation is required for substrate recognition.

### Biochemical analysis of the TRIM37–Cep192(IDR+ASH8) complex

**Figure 5.**
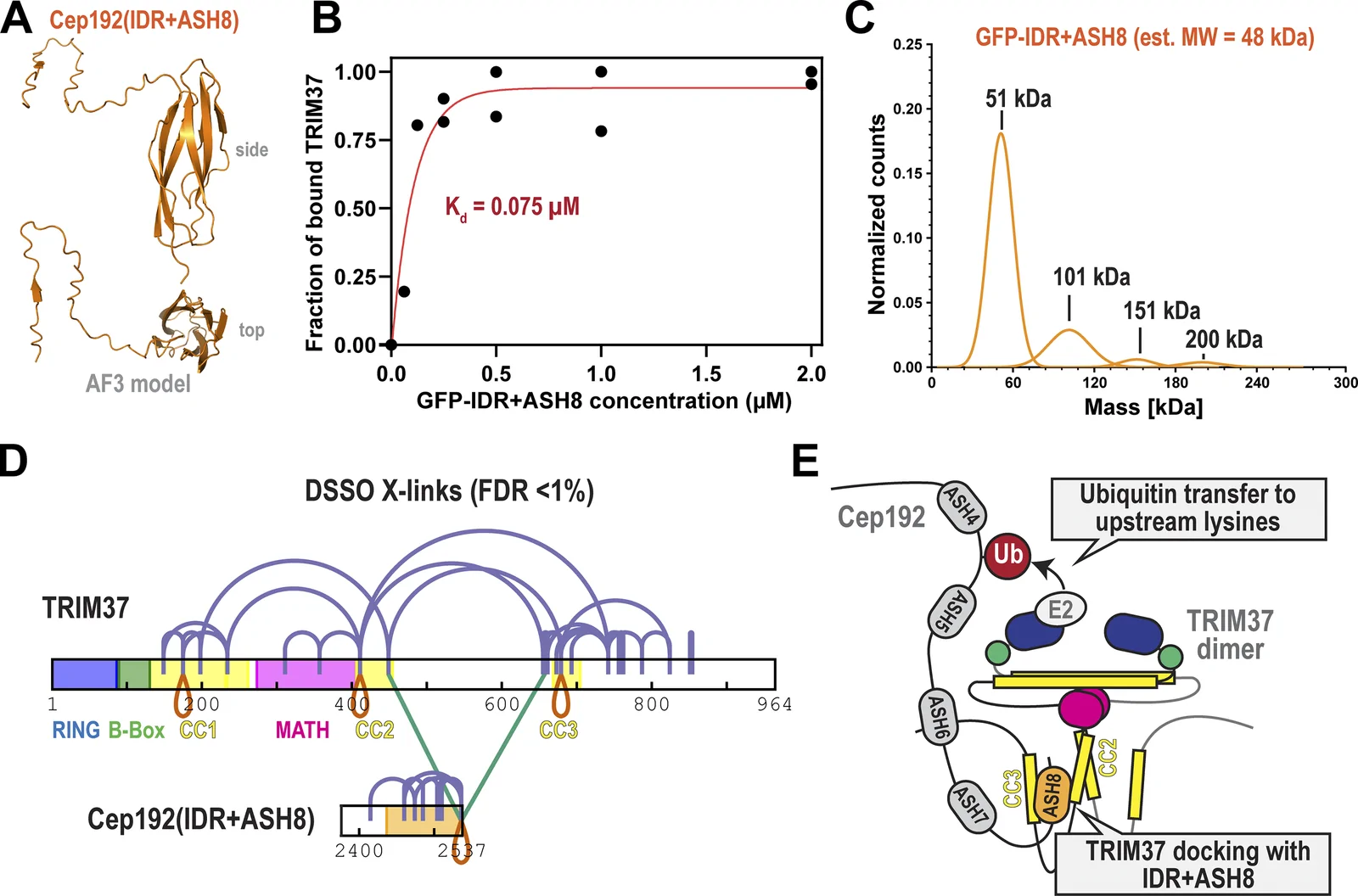
**Structural analysis of the TRIM37–IDR+ASH8 complex. (A)** AF3 prediction of the IDR+ASH8 domain of Cep192. Side and top views are shown. pTM = 0.68 for the whole domain; pTM = 0.86 for ASH8. **(B)** Binding capacity of purified GFP-IDR+ASH8 with 30 nM TRIM37. The amount of TRIM37 remaining in the supernatant after construct binding was measured to calculate the fraction of bound TRIM37 at each concentration of GFP-IDR+ASH8. K_d_ was determined from a one-phase association fit (red line; R^2^ = 0.93). *n* = 2 biological replicates. Associated with Fig. S4, A and B. **(C)** Mass photometry of 20 nM GFP-IDR+ASH8. The data represent three biological replicates. **(D)** DSSO cross-linking map of TRIM37 and IDR+ASH8. ASH8 is colored orange; IDRs are colored white. Cross-links: purple, intramolecular; green, intermolecular; red, intermolecular links between the same amino acid. Three biological replicates, FDR <1%. Associated with Fig. S4, C and D. **(E)** Model for how TRIM37 dimers recognize and ubiquitinate the C terminus of Cep192.

We next sought to define the structural mechanism by which TRIM37 recognizes the IDR+ASH8 module. The only known substrate motif recognized by TRIM37 is a disordered peptide motif within Centrobin (Bellaart et al., 2025). In contrast, AlphaFold3 predicts a tight β barrel structure for ASH8 (Fig. 5 A). Thus, we hypothesized that TRIM37 has multiple modes of substrate recognition.

**Figure S4.**
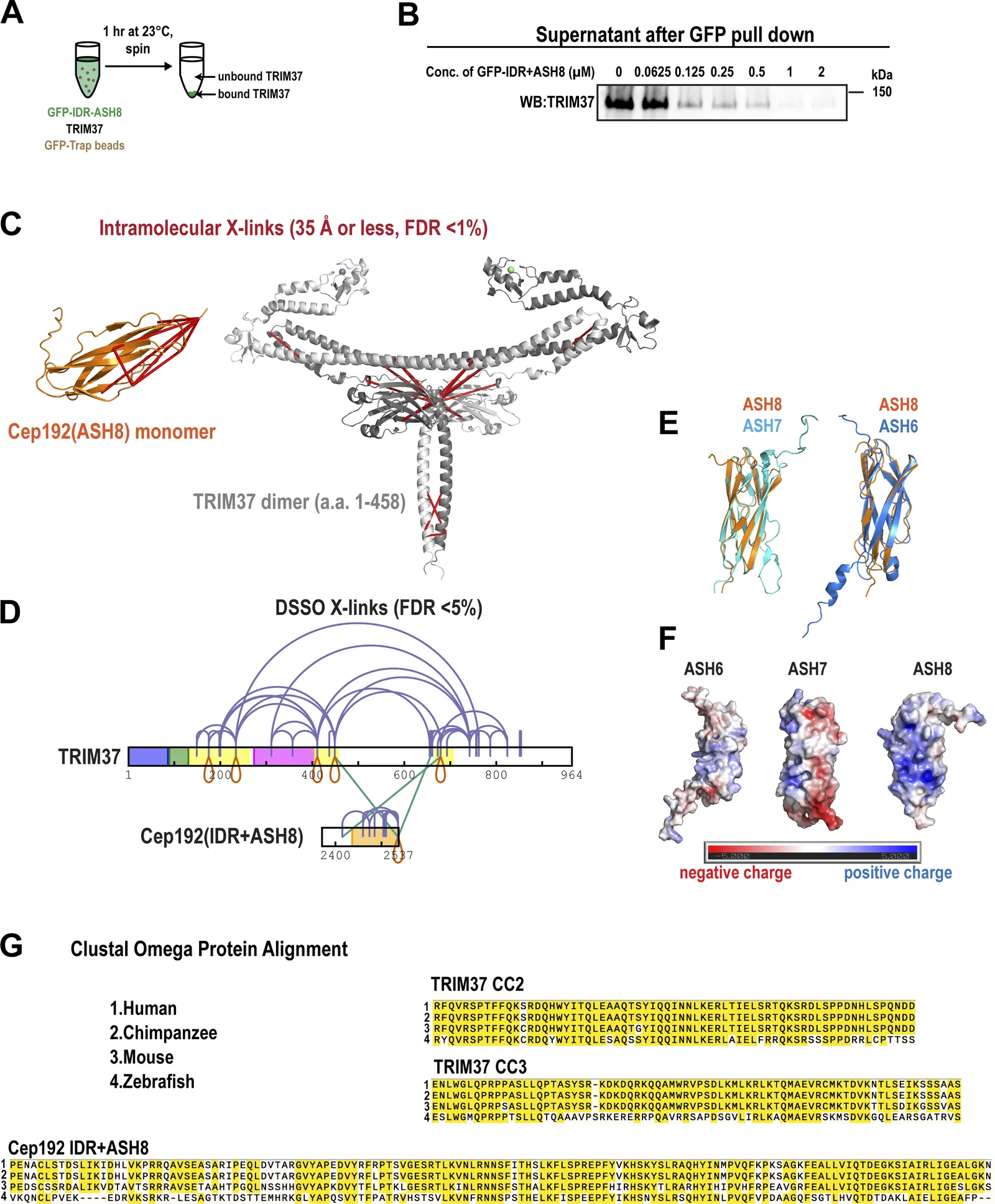
**Biochemical characterization of the IDR+ASH8–TRIM37 interaction. (A)** Schematic of pull-down assay from Fig. 5 B. **(B)** Western blot of TRIM37 found in the supernatant of experiments from Fig. 5 B. **(C)** Cross-links from Fig. 5 D mapped onto AF3 predicted structures of the ASH8 domain and a TRIM37 dimer (aa 1–458). Shown are cross-links that satisfy the maximum distance constraint for DSSO (35 Å; between α carbons). **(D)** DSSO cross-linking map of TRIM37 and IDR+ASH8 with calculated FDR <5% (medium confidence). ASH8 is colored orange; IDRs are colored white. Cross-links: purple, intramolecular; green, intermolecular; red, intermolecular links between the same amino acid. *n* = 3 biological replicates. **(E)** Overlay of ASH8 (AF3 model) with ASH7 (PDB: 6FVI) and ASH6 (AF3 model). **(F)** Estimated surface charge of structures in F (blue, positive; red, negative). **(G)** Protein sequence conservation of Cep192- and TRIM37-binding regions. Yellow highlights conserved residues. Source data are available for this figure: SourceData FS4.

We first measured binding affinity. We incubated 30 nM TRIM37 with increasing concentrations of GFP-IDR+ASH8, the minimal binding construct, and GFP-Trap beads. After 1 h, we spun down the beads and measured the concentration of unbound TRIM37 that remained in the supernatant (Fig. S4, A and B) (Pollard, 2010). Plotting the bound fraction of TRIM37 vs. IDR+ASH8 concentration revealed that the binding occurred with mid-nanomolar affinity (apparent K_d_ = 75 nM; Fig. 5 B). Mass photometry revealed that GFP-IDR+ASH8 is largely monomeric but can form small populations of dimers, trimers, and tetramers (Fig. 5 C). This result indicates that TRIM37 can recognize a non-oligomeric domain with high affinity.

Next, we performed cross-linking MS (XL-MS) to map the interactions between TRIM37 and IDR+ASH8. We used 1 mM DSSO, an amine-reactive, MS-cleavable molecule that links lysine residues. MS analysis with data filtering (<1% calculated false discovery rate [FDR], *n* = 3 replicates) identified 74 total cross-links with high confidence: 55 specific to TRIM37 molecules, 19 specific to IDR+ASH8, and 2 between TRIM37 and IDR+ASH8 (Fig. 5 D). When overlaid onto AlphaFold3 models, all cross-links within IDR+ASH8 satisfied distance constraints expected for DSSO (<35 Å; Fig. S4 C), supporting that ASH8 conforms to a compact β barrel structure. An intermolecular self-link (K2536–K2536; red loop in Fig. 5 D) indicates that IDR+ASH8 can at least form dimers, consistent with mass photometry. We also identified three self-links within TRIM37 (K175–K175, K411–K411, and K679–K679), consistent with TRIM37 forming dimers in cells (Fig. 5 D) (Yeow et al., 2025). In Fig. S4 C, we show cross-links that satisfy distance constraints when mapped onto an AlphaFold3 model of the dimeric N terminus (aa 1–458), which has well-defined structural motifs. These results support the accuracy of our cross-link assignments.

XL-MS revealed that the ASH8 β barrel interacts with the coiled-coil domain (CC2) following the MATH domain and a predicted coiled-coil domain (CC3) in the tail of TRIM37 (Fig. 5 D; FDR < 1%). By choosing a medium confidence cutoff (FDR < 5%), we revealed an additional interaction between CC3 and the IDR just upstream of ASH8 (Fig. S4 D). These data are consistent with our biochemical results showing that both the IDR and ASH8 are needed for TRIM37 binding (Fig. 1). Furthermore, we identified numerous high-confidence cross-links within CC3, including a self-link at K679, suggesting that this region may represent a new structural motif. Cross-links between CC3, the intrinsically disordered tail, and CC2 indicate that TRIM37’s tail (a0a 459–964) interacts with the N terminus. Our data support a model whereby TRIM37 uses two separate coiled-coil motifs to bind the bipartite IDR+ASH8 motif with high affinity.

## Discussion

How E3 ubiquitin ligases target specific proteins is an important but poorly understood area of cell biology. In this study, we revealed how the E3 ubiquitin ligase TRIM37 targets Cep192, a protein essential for centrosome assembly and function. We demonstrated that TRIM37 directly engages Cep192 through a defined C-terminal module called IDR+ASH8. TRIM37 then ubiquitinates an upstream cluster of 7 lysine residues on Cep192 (Fig. 5 E). Ubiquitination reduces the stability of Cep192, which regulates Cep192 steady-state levels and centrosome size. Finally, we showed that the IDR+ASH8 module can transform GFP-EB1 or GFP alone into a TRIM37 substrate, establishing that IDR+ASH8 is a *bona fide* degron. Thus, our findings provide a structural mechanism for how TRIM37 contributes to centrosome homeostasis.

Our data demonstrate that TRIM37-mediated ubiquitination is required for Cep192 degradation and that this process depends on both the TRIM37-binding region and the lysines that accept ubiquitin. In cells, eliminating the TRIM37-interaction site (ΔASH8) abolished degradation and completely prevented TRIM37 binding, showing that access to this motif is essential for initiating the reaction. Likewise, substituting the seven ubiquitinated lysines with arginines (7KtoR) stabilized Cep192 and blocked its ubiquitination, establishing that these residues are required for substrate degradation. Notably, although 7KtoR still engaged TRIM37, its reduced binding compared with the WT protein suggests that lysine availability could regulate ligase association. This decreased affinity further supports the idea that ubiquitination weakens TRIM37–Cep192 interactions. Such ubiquitin-triggered substrate release is consistent with established mechanisms for other RING E3s, including SCF(Cdc4) and APC/C (Deffenbaugh et al., 2003; Sitry-Shevah et al., 2024).

Overexpression of both Cep192 mutants (7KtoR and ΔASH8), but not Cep192(WT), caused cell cycle arrest in metaphase and eventually death. The molecular cause of this phenotype is unclear. Extended mitosis was observed in TRIM37-depleted cells (Meitinger et al., 2021), but this phenotype was rescued by Centrobin depletion, suggesting that excessive accumulation of Cep192 was not the direct cause of cell cycle arrest. We found that both Cep192 mutants exhibited reduced fluorescence intensity at mitotic centrosomes. Compared with the spherical centrosomes observed in Cep192(WT)-expressing cells, mutant centrosomes appeared slightly flatter and more elongated. These changes suggest that the mitotic PCM assembly is compromised. In addition, cells expressing the mutants exhibited denser mitotic spindles, which may contribute to the observed mitotic delay. These findings are unexpected and raise the intriguing possibility that Cep192 ubiquitination acts as an important posttranslational signal that supports correct PCM maturation during mitosis. This could impact cell proliferation given that mitotic PCM assembly affects the speed and fidelity of mitotic division in many species (Hamill et al., 2002; Lawo et al., 2012; Conduit et al., 2014; Cabral et al., 2019; Meitinger et al., 2020). These data align with earlier reports that observed increased levels of ubiquitinated proteins at the centrosome in metaphase relative to S phase (Kimura et al., 2014). However, we must express caution in interpreting these results, as they come from cells overexpressing transgenes. Together, these observations point to regulatory layers that extend beyond the scope of the present study and warrant further investigation.

While the functional consequences of ubiquitination-deficient Cep192 are intriguing, the central focus of this work was to define how TRIM37 identifies its substrates. We found that TRIM37 recognizes a specific module comprising the ASH8 domain and its adjacent linker (IDR+ASH8). Moreover, we demonstrated that the fusion of the IDR+ASH8 module to an unrelated protein is sufficient to trigger its degradation via TRIM37. AlphaFold3 predicts that the IDR region is not structured and that the ASH8 domain conforms to a compact β barrel (35 Å length, 12 Å width, and 39 Å in diameter) (Abramson et al., 2024). Interestingly, several TRIM37 interactors identified by proximity labeling (Yeow et al., 2020; Yeow et al., 2025) are predicted to contain similar structures: Cep170B, C2CD3, RPGRIP1L, TJP1, and MIS18BP1. This raises the possibility that a compact β barrel could be a general targeting motif for TRIM37. However, simply having a compact β barrel is not sufficient, since full-length Cep192 contains 8 β barrels (all ASH-like domains), but only the last one engages TRIM37. Structural comparisons of predicted ASH8 and ASH6 with the solved ASH7 structure (PDB: 6FVI) reveal that ASH8 lacks several protrusions found in the others (Fig. S4 E). Moreover, ASH8 has a more positively charged surface (Fig. S4 F). Identifying the functional epitope within ASH8 will be crucial for defining these structural distinctions that permit TRIM37 recognition. This pursuit would be aided by identifying additional *bona fide* substrates of TRIM37 and determining the similarities between their docking motifs.

Our work raises the possibility that TRIM37 has multiple modes of substrate recognition tailored for specific functions. Recent work demonstrated that a small peptide motif mediates the interaction between TRIM37 and Centrobin to prevent the formation of pathological PCM-like condensates (Balestra et al., 2021; Bellaart et al., 2025; Meitinger et al., 2021). In this case, TRIM37 uses its MATH domain to bind weakly (K_d_ = 14 μM) to Centrobin’s peptide motif. It was hypothesized that weak binding was a key feature of this regulatory system. Large-scale multimerization of the substrate would create a high local concentration of binding sites, thus attracting TRIM37 through an avidity effect. This would also promote TRIM37 multimerization, which is known to increase its ligase activity (Bellaart et al., 2025). By this mechanism, TRIM37 would only ubiquitinate and destroy aggregated forms of Centrobin. In contrast, our results demonstrate that TRIM37 can interact with a non-multimeric substrate, the IDR+ASH8 domain of Cep192. Our XL-MS results showed that two separate coiled-coil domains (CC2, CC3) on TRIM37 connect with ASH8 (Fig. 5 D), suggesting that the MATH domain is not involved. Centrobin contains no β barrels, while the C terminus of Cep192 does not contain the same IDR found in Centrobin. These data are consistent with a model whereby TRIM37 has two modes of substrate docking: (1) low-affinity, MATH domain-mediated recognition of a disordered peptide motif, and (2) high-affinity, coiled-coil-mediated recognition of an ASH8-like β barrel with a preceding IDR. We speculate that TRIM37 may switch between these modes depending on cellular context, enabling it to regulate diverse substrates with different structural features and functional consequences. For example, the low-affinity, avidity-dependent module may be important for disassembling supramolecular complexes (e.g., Centrobin condensates or multimeric proteins within the PCM), while the high-affinity module may be important for regulating unassembled, cytoplasmic substrates (e.g., Cep192 prior to incorporation in PCM).

Together, our findings provide a new mechanism for TRIM37 substrate recognition, revealing that the ligase can engage clients through high-affinity binding to a C-terminal bipartite motif. The IDR+ASH8 module of Cep192 acts as a transferable degron, explaining how TRIM37 regulates Cep192 levels and potentially other substrates. This mechanism is likely active in other species, since IDR+ASH8 and the coiled-coil recognition motifs of TRIM37 are well conserved amongst vertebrates (Fig. S4 G). Moreover, our discovery that the IDR+ASH8 module functions as a TRIM37-specific degron has broader implications for the hundreds of TRIM-family and other RING-type E3 ligases for which substrate recognition rules have yet to be defined (Zhang et al., 2025). The portability of this bipartite degron suggests that similar mixed-architecture motifs may exist across the proteome but remain unrecognized.

## Materials and methods

### Cell culture

DLD-1 cells (CCL-221) were cultured in DMEM (10-017-CV; Corning) supplemented with 10% fetal bovine serum (10082147; Gibco) and 100 U/ml penicillin-streptomycin (P4333; Sigma-Aldrich). All cell lines were maintained at 37°C in a humidified incubator with 5% CO_2_ and 21% oxygen. The DLD-1 cell line with the Flp-In T-REx system was generously provided by the Peter Ly laboratory (Tighe et al., 2004).

### Generation of cell lines expressing protein constructs

Cep192 (cDNA clone NM_032142.3; GenScript), EB1 (Woodruff et al., 2017; Zanic et al., 2009), and GFP constructs were made via PCR amplification and verified through Sanger sequencing. All plasmids used to transfect the DLD-1 cell line are listed in Table S3. Stable, isogenic cell lines overexpressing Cep192, EB1, or GFP variants were established by co-transfecting the pcDNA5 plasmids with the pOG44 FLP recombinase vector using JetPrime reagent. Positive clones were selected with 100 µg/ml hygromycin (Millipore Sigma), and construct expression was induced by treating cells with 2 µg/ml tetracycline (Millipore Sigma). For monitoring construct stability, we first overexpressed each construct for 96 h by adding tetracycline to the media (2 µg/ml). After imaging the 96 h expression time point, we washed out cells three times with PBS to remove tetracycline completely. We imaged cells again 24 and 48 h after tetracycline washout to track the construct degradation process.

### Protein expression and purification

Cep192 constructs were inserted into a baculoviral expression plasmid (pOCC29) using standard restriction cloning. Baculoviruses were generated using the FlexiBAC system (Lemaitre et al., 2019) in SF9 cells. We used SF9-ESF *Spodoptera frugiperda* insect cells (Expression Systems) grown at 27°C in ESF 921 Insect Cell Culture Medium (Expression Systems) supplemented with FBS (2% final concentration). Protein was harvested 72 h after infection during the P3 production phase. Cells were collected, washed, and resuspended in harvest buffer (25 mM HEPES, pH 7.4, and 150 mM NaCl). For the expression of smaller Cep192 truncations and EB1 constructs, we used BL21(DE3) bacterial cells. All expression plasmids are listed in Tables S1 and S2.

All subsequent protein purification steps were performed at 4°C. Cell pellets were resuspended in Lysis Buffer (25 mM HEPES, pH 7.4, 30 mM imidazole, 500 mM KCl, 0.5 mM DTT, 1% glycerol, and 0.1% CHAPS) + protease inhibitors and then lysed using a Dounce homogenizer. Proteins were bound to Ni-NTA (Qiagen), washed with 10 column volumes of Lysis Buffer, and eluted with 250 mM imidazole. The eluates were concentrated using Ultra-15 Centrifugal Filters (Millipore) and incubated with 3C PreScission/TEV protease overnight to remove purification tags. Then we ran purified proteins through AKTA FPLC using Superdex 200 (28-9909-44; Cytiva) or Superdex 75 (29148721; Cytiva) Increase 10/300 Gl column, depending on the size of the protein. During gel filtration, all proteins were in Gel Filtration Buffer (25 mM HEPES, pH 7.4, 500 mM NaCl, 0.5 mM DTT, 1% glycerol, and 0.1% CHAPS). All eluted proteins were then concentrated and aliquoted in PCR tubes, flash-frozen in liquid nitrogen, and stored at −80°C. Protein concentration was determined by measuring absorbance at 280 nm using a NanoDrop ND-1000 spectrophotometer (Thermo Fisher Scientific).

### *In vitro* co-immunoprecipitation

Purified Cep192 constructs (1 µM) and EB1 (1 µM, control) were incubated with TRIM37 (1 µg) for 1 h at 37°C with rotation. GFP-Trap Magnetic Particles M-270 (gtd-20; Chromotek) or Dynabeads Protein A (conjugated with TRIM37 antibody, Bethyl A301-174A) were pre-washed with buffer (150 mM KCl, 25 mM HEPES, 1% glycerol, and 0.05% NP-40). The TRIM37-GFP protein reactions were incubated with 25 μl pre-washed beads at room temperature for 10 min with rotation, followed by incubation at 4°C for 1 h with rotation. Then beads were washed three times with washing buffer, followed by one additional wash with a higher salt washing buffer (500 mM KCl, 25 mM HEPES, 1% glycerol, and 0.05% NP-40). We eluted the bound fraction by incubating with SDS sample buffer at 95°C for 5 min, and the bound components were detected by western blotting.

### *In vitro* ubiquitination assays

For the experiment in Fig. 1 A, we combined ligases previously associated with TRIM37 (Bhatnagar et al., 2014): 100 nM E1 UBE1 (E-305-025; Bio-Techne) and 100 nM E2 UbcH5b/UBE2D2 (E2-622-100; Bio-Techne). We added 10 µM HA-Ubiquitin (gifted from V. Tagliabracci, UT Southwestern Medical Center, Dallas, TX, USA), 1 µM purified Cep192 protein, and ubiquitin reaction buffer (5 mM MgCl_2_, 0.5 mM DTT, 2mM ATP, 25 mM HEPES, 100 mM KCl, and 100nM ZnCl_2_). To the positive sample, we added 1 µM TRIM37, while the negative sample did not contain any E3 ligase. We sent the samples for identification of posttranslational modifications, specifically for GG marks that are left after ubiquitination.

For the experiment in Fig. 1 H, we first pulled down GFP-Cep192 constructs from DLD-1 cells. Cells were harvested from 10-cm plates, counted, and 8 × 10^6^ cells were used per condition. Cells were washed once with ice-cold PBS supplemented with protease inhibitor cocktail (539134-1SET; Millipore). Cell lysis was performed with lysis buffer (500 mM KCl, 25 mM HEPES [pH 7.4], 1% glycerol, and 1% NP-40) and sonication for 15 s at 30% amplitude. Lysates were passed over GFP-Trap beads, then washed 3× in 150 mM KCl, 25 mM HEPES (pH 7.4), 1% glycerol, and 0.05% NP-40. To these beads we added the ubiquitin reaction mixture: 10 nM E1 UBE1, 10 nM E2 UbcH5b/UBE2D2, 0.5 µM HA-Ubiquitin (U-110, R&D systems), 25 nM TRIM37 (ab269066; Abcam), and ubiquitin reaction buffer (5 mM MgCl_2_, 0.5 mM DTT, 2mM ATP, 25 mM HEPES, 100 mM KCl, and 100 nM ZnCl_2_). This reaction was incubated for 1 h at 37°C. Western blots were performed against HA and GFP tags.

### MS identification of ubiquitinated sites

Samples were digested overnight with trypsin (Pierce) following reduction and alkylation with DTT and iodoacetamide (Sigma-Aldrich). Following solid-phase extraction cleanup with an Oasis HLB µelution plate (Waters), the resulting peptides were reconstituted in 10 µl of 2% (vol/vol) acetonitrile (ACN) and 0.1% trifluoroacetic acid in water. 5 ul of this was injected onto an Orbitrap Eclipse mass spectrometer (Thermo Fisher Scientific) coupled to an Ultimate 3000 RSLC-Nano liquid chromatography system (Thermo Fisher Scientific). Samples were injected onto a 75 μm i.d., 75-cm-long Easy-Spray column (Thermo Fisher Scientific), and eluted with a gradient from 0 to 28% buffer B over 90 min. Buffer A contained 2% (vol/vol) ACN and 0.1% formic acid in water, and buffer B contained 80% (vol/vol) ACN, 10% (vol/vol) trifluoroethanol, and 0.1% formic acid in water. The mass spectrometer operated in positive ion mode with a source voltage of 2.5 kV and an ion transfer tube temperature of 300°C. MS scans were acquired at 120,000 resolution in the Orbitrap, and up to 10 MS/MS spectra were obtained in the Orbitrap for each full spectrum acquired using higher-energy collisional dissociation for ions with charges 2–7. Dynamic exclusion was set for 25 s after an ion was selected for fragmentation. Each sample was injected twice.

Raw MS data files were analyzed using Proteome Discoverer v.3.0 (Thermo Fisher Scientific), with peptide identification performed using Sequest HT searching against the human reviewed protein database from UniProt (downloaded January 4, 2024, 20354 entries). Fragment and precursor tolerances of 10 ppm and 0.6 Da were specified, and three missed cleavages were allowed. Carbamidomethylation of Cys was set as a fixed modification, with oxidation of Met and GlyGly modification of Lys set as variable modifications. The FDR cutoff was 1% for all peptides. The sum of peak intensities for all peptides matched to a protein was used as protein abundance values. A list of identified peptides together with PTM sites found can be found in Data S1.

### Cross-linking MS (XL-MS)

Reactions were assembled using purified proteins at final concentrations of 5 µM TRIM37(C18R) (Table S1) and 5 µM IDR+ASH8 (Table S2). The proteins were combined with 100 nM ZnCl_2_, 25 mM HEPES, and 150 mM KCl (pH 7.4). Reactions were incubated for 20 min at 37°C with shaking at 350 rpm. Cross-linking was initiated by adding DSSO to a final concentration of 1 mM, followed by a 5-min incubation. The reaction was then quenched with ammonium bicarbonate (50 mM final) and incubated for an additional 30 min at 37°C with shaking at 350 rpm. Samples were denatured by adding 6× SDS sample buffer and heating for 5 min at 95°C. Proteins were separated by SDS-PAGE, stained with Coomassie Blue, and the band corresponding to the TRIM37–ASH8 complex was excised. Gel slices were submitted for MS analysis, requesting three injections per sample.

Samples were digested overnight with trypsin (Pierce) following reduction and alkylation with DTT and iodoacetamide (Sigma–Aldrich). Following solid-phase extraction cleanup with an Oasis HLB µelution plate (Waters), the resulting peptides were reconstituted in 10 μl of 2% (vol/vol) ACN and 0.1% trifluoroacetic acid in water. Three aliquots of each sample were separately injected onto an Orbitrap Fusion Lumos mass spectrometer coupled to an Ultimate 3000 RSLC-Nano liquid chromatography system. Samples were injected onto a 75 um i.d., 75-cm-long Easy-Spray column (Thermo Fisher Scientific) and eluted with a gradient at a flow rate of 250 nl/min from 0 to 5% buffer B over 1 min, 5–40% B over 60 min, 40–99% over 25 min, and held at 99% B for 5 min before returning to 0% B for column equilibration. Buffer A contained 2% (vol/vol) ACN and 0.1% formic acid in water, and buffer B contained 80% (vol/vol) ACN, 10% (vol/vol) trifluoroethanol, and 0.1% formic acid in water. The mass spectrometer operated in positive ion mode with a source voltage of 2.0 kV and an ion transfer tube temperature of 275°C. MS scans were acquired at 120,000 resolution in the Orbitrap. MS/MS spectra were obtained in the Orbitrap using top-speed mode (5 s) for each full spectrum acquired using collision-induced dissociation (CID) for ions with charges 3–7. Dynamic exclusion was set for 25 s after an ion was selected for fragmentation. MS2 fragment ions with 1–4 charge states and a mass difference of 31.9721 with 10–100% intensity range were selected for MS3 CID fragmentation, with a mass tolerance of 25 ppm, 2 m/z isolation window, and at 15,000 resolution.

Raw MS data files were analyzed using Proteome Discoverer v.3.0 (Thermo Fisher Scientific), with peptide identification performed using MS Annika 2.0 (Birklbauer et al., 2023) searching against the amino acid sequences of TRIM37 and CEP192. Fragment and precursor tolerances of 10 ppm (MS1), 20 ppm (MS2), and 20 ppm (MS3) were specified, and up to four missed cleavages were allowed. DSSO (+158.004 Da, modification on K) was set as the crosslinker modification, with N-terminal crosslinks allowed. Diagnostic ions of 138.0911, 155.1179, 170.0634, and 187.0900 were included. Carbamidomethylation of Cys was set as a fixed modification, with oxidation of Met and DMSO modification of Lys set as variable modifications. The FDR cutoff was 1% (i.e., estimated FDR) at the CSM and cross-link levels. The peak intensities of the peptides were used as abundance values. Calculated FDRs ranged from 5 to 7% for all cross-links (see Data S2). In Fig. 5, the most confident cross-links (calculated FDR <1%) were chosen and visualized with xiVIEW (www.xiview.org).

### Western blotting

Samples were incubated with SDS sample buffer for 5 min at 95°C and separated by SDS-PAGE. Protein from each gel was transferred to a nitrocellulose membrane using a Trans-Blot Turbo transfer for high molecular weight proteins (10 min). Membranes were incubated in a blocking buffer consisting of 1× TBS-T + 3% Blotting-Grade Blocker (Bio-Rad), shaking at room temperature for 1 h. Membranes were then washed three times with fresh 1× TBS-T and incubated with primary antibodies, shaking overnight at 4°C. The primary antibody was washed three times with fresh 1× TBS-T and incubated by shaking with secondary antibodies at room temperature for 1 h. The membrane was washed hree times with 1x TBS-T, then once with TBS. Each membrane was then incubated in ECL reagent (Thermo Fisher Scientific, SuperSignal West Femto) for 5 min and imaged with a ChemiDoc Touch Imaging System. Primary antibodies: Mouse anti-α tubulin (3873S; Cell Signaling Technologies, 1:1,000), Goat anti-enhanced GFP (1:5,000; Dresden PEP facility, 15 mg/ml; [Rios et al., 2024]), and Rabbit anti-TRIM37 (1:1,000; A301-174A; Bethyl/Thermo Fisher Scientific); secondary Antibodies (1:20,000-1:100,000): HRP-conjugated Goat anti-Rabbit IgG (1 mg/ml) (65-6120; Invitrogen), HRP-conjugated Goat anti-Mouse (1.5 mg/ml) (62-6520; Invitrogen), and HRP-conjugated Donkey anti-Goat (1 mg/ml) (A15999; Invitrogen).

### RNA interference

For TRIM37 silencing, DLD-1 cells were transfected with siTRIM37 (siRNA ID 144318; Thermo Fisher Scientific) or Stealth Negative Control (452002; Invitrogen) using Lipofectamine RNAiMAX reagent.

### Imaging

Cells were seeded onto a 24-well glass-bottom tissue culture plate (229125; CELLTREAT) and then grown for 48 h before imaging. Each cell line was imaged in DMEM without phenol red (21063-029; Gibco). Live-cell imaging was performed inside a stage-top incubator (Tokai Hit STX), maintained at 37°C with 5% CO_2_. Images were acquired using an inverted Nikon Eclipse Ti2-E microscope equipped with a Yokogawa CSU-W1 confocal scanner unit, piezo Z stage, and an iXon Ultra 888 EMCCD camera (Andor), controlled by Nikon Elements software.

A 60× 1.2 NA Plan Apochromat water-immersion objective was used to acquire 31 Z-stacks (0.5-µm steps), each with 100 ms laser exposure. Fluorescence imaging was performed sequentially using the following settings:

• 488 nm excitation (15% laser power, 2 × 2 binning)
• 561 nm excitation (15% laser power, 1 × 1 binning)
• 640 nm excitation (10% laser power, 1 × 1 binning).

For imaging mitotic progression, we first expressed constructs inside cells using tetracycline treatment for 24–72 h. Then we arrested cells in late G2 phase by incubating them overnight with 9 µM CDK1 inhibitor (RO-3306, S7747; Selleck Chemicals). After washing out the drug, we waited an hour to make sure most cells would enter mitosis. Then we imaged cells using 5% laser power with 1 × 1 binning.

For DIC imaging, we plated cells 3 days before imaging on glass-bottom microwell dishes (35 mm Mattek, P35G1.514C.S) and incubated them with tetracycline to induce construct expression. Then we imaged cells using the same microscope as described above with DIA Illuminator, Iris intensity 92.3, exposure for 100 ms (1 × 1 binning), 21 Z stacks (1 µm steps), imaging every 10 min for 20 h.

For SoRa imaging, super-resolution images were taken using a inverted Nikon ECLIPSE Ti2 equipped with a Yokogawa Spinning Disk system (Super Resolution by Optical Pixel Reassignment System; CSU-W1 SoRa) and an ORCA-Fusion CMOS camera (HAMAMATSU) controlled by NIS-Elements software. To investigate spindle and centrosome morphology, we visualized the IF samples using a 60× 1.42-NA Plan Apochromat oil objective with 4× intermediate magnification. Every image was taken using the following settings: 41 Z-stacks (0.2-µm steps), each with 100 ms laser exposure (1 × 1 binning for each).

### Image quantification and statistical analysis

Images were analyzed in FIJI using semiautomated, threshold-based particle analysis with a defined ROI for every GFP focus or cytoplasmic measurement. Data were plotted, and statistical tests were performed using GraphPad Prism. The sample size, measurement type, error type, and statistical test are described in the figure legends, where appropriate.

### Construct degradation during cell cycle arrest

To induce construct expression, cells were incubated with tetracycline for 96 h and then imaged. Following initial imaging, tetracycline was washed out, and the culture medium was replaced with serum-depleted media (DMEM + 1% penicillin-streptomycin, without FBS). Cells were then incubated for an additional 48 h to induce cell cycle arrest in G_0_ and then imaged.

### Immunofluorescence

A day before tetracycline induction, cells were plated onto collagen-coated 24-well glass plates (CLS354236; Corning). To induce Cep192 overexpression, tetracycline was added to a final concentration of 2 µg/ml, and cells were incubated for 96 h. Cells were then washed once with PBS and fixed in 100% methanol for 15 min at −30°C. After fixation, methanol was removed by rinsing with PBS and incubating the plate overnight at 4°C in the dark. The following day, cells were permeabilized with 0.5% Triton X-100 for 5 min at room temperature and then blocked with 5% BSA in TBS for 30 min. Cells were incubated with primary antibodies conjugated with fluorophores for 48 h at 4°C in the dark: anti-Centrin-1 (conjugated with CoraLite591; CL594-12794; Proteintech, 1:500), anti-tubulin (conjugated with Alexa 647, ab195884; Abcam; 1:1,000), and anti-GFP (conjugated with Alexa 488; A-21311; Thermo Fisher Scientific; 1:500). After incubation, cells were washed twice with PBS, with a 10-min room temperature incubation during the second wash. Nuclei were stained overnight at 4°C in the dark using DAPI (62248; Thermo Fisher Scientific; 1:1,000 in PBS). Prior to imaging, cells were rinsed once with PBS.

### Cell viability assay

For each construct, cells were plated in triplicate on a 6-well plate at a density of 1 × 10^5^ cells per well. Tetracycline was added to the culture medium at a final concentration of 2 µg/ml to induce construct expression. We counted the number of cells on days 1, 3, and 6 using a hemocytometer, with all cells passaged back into culture after counting. The experiment was performed three independent times, and the mean cell number for each day of construct expression was plotted.

### qPCR

We used the Arcturus PicoPure RNA Isolation kit (12204-01; Thermo Fisher Scientific) to purify RNA from the lysed cells expressing each construct for 96 h, collected in triplicate. We measured RNA concentration using a NanoDrop and saved 1 µg of RNA for further analysis. We performed cDNA synthesis from RNA using iScript Reverse Transcript Supermix for RT-qPCR (1708841; Bio-Rad) following the product protocol. Then we set up the RT-qPCR reaction using SsoAdvanced Universal SYBR Green Supermix (1725271; Bio-Rad) and primers detecting GFP (Fw- 5′-GGA​CGA​CGG​CAA​CTA​CAA​GA-3′, Rv –5′-AAG​TCG​ATG​CCC​TTC​AGC​TC-3′) and housekeeping gene 36B4 as a control (Fw –5′-AAC​ATG​CTC​AAC​ATC​TCC​CC-3′, Rv- 5′-CCG​ACT​CCT​CCG​ACT​CTT​C-3′) and following the product protocol.

### Mass photometry

GFP-Cep192(IDR+ASH8) was centrifuged to remove potential aggregates, then diluted into PBS to a final concentration of 20–50 nM on a clean glass coverslip. Protein was analyzed using a TwoMP mass photometer (Refeyn) using four species of BSA (monomer, dimer, trimer, and tetramer) to create a molecular weight standard curve.

### Protein affinity assay

Purified TRIM37 and GFP-Cep192(IDR+ASH8) were dialyzed and diluted using binding buffer (25 mM HEPES, pH 7.4, 150 mM KCl, and 1 mM DTT). 30 nM TRIM37 was incubated with a range of GFP-Cep192(IDR+ASH8) concentrations, GFP-Trap Magnetic Particles (Proteintech), and binding buffer to a final volume of 50 μl. After 1-h incubation at room temperature, beads were sedimented, and the supernatant was collected and analyzed by SDS-PAGE and western blotting against TRIM37. Signal was measured using FIJI, the background signal was subtracted, and the remaining signal was normalized to the negative control (no Cep192). The fraction of bound TRIM37 is defined as one- (normalized supernatant signal).

### Online supplemental material

Fig. S1 shows identification of ubiquitinated residues and the TRIM37-binding site on Cep192. Fig. S2 shows analysis of centrosome and spindle shape in cells expressing Cep192 constructs. Fig. S3 shows testing of degron efficiency with a non-centrosomal protein. Fig. S4 shows biochemical characterization of the IDR+ASH8–TRIM37 interaction. Table S1 shows baculovirus constructs for protein expression. Table S2 shows bacterial constructs for protein expression. Table S3 shows constructs for protein expression using the TREx Flp-In system in DLD-1 human colon cancer cells. Data S1 shows MS data for detection of ubiquitinated sites on Cep192. Data S2 shows XL-MS data.

## Supplementary Material

Review History

Table S1 shows baculovirus constructs for protein expression.

Table S2 shows bacterial constructs for protein expression.

Table S3 shows constructs for protein expression using the TREx Flp-In system in DLD-1 human colon cancer cells.

Data S1 shows MS data for detection of ubiquitinated sites on Cep192.

Data S2 shows XL-MS data.

SourceData F1 is the source file for Fig. 1.

SourceData F4 is the source file for Fig. 4.

SourceData FS1 is the source file for Fig. S1.

SourceData FS3 is the source file for Fig. S3.

SourceData FS4 is the source file for Fig. S4.

## Data Availability

MS raw data are accessible from MassIVE (accession number MSV000100205). Processed MS data are available in the Supplemental data sets. All other data are available upon reasonable request.
