# Supplementary material for: TRIM37 recognizes a bipartite degron to ubiquitinate centrosome substrates
Source: J Cell Biol. 2026 Nov 10;226(1):e202608031. doi: 10.1083/jcb.202608031 (PMC13750308; doi:10.1083/jcb.202608031)
Supplement: Supplementary file 1 [file jcb_202608031_review_history.pdf]

TRIM37 recognizes a bipartite degron to ubiquitinate centrosome substrates

Weronika Stachera, Judith Tafur, Kan Yaguchi, Nicole Familiari, Manolo Rios, and Jeffrey Woodruff

Corresponding Author(s): Jeffrey Woodruff, The University of Texas Southwestern Medical Center

Review Timeline:

Submission Date:	2026-08-11
Editorial Decision:	2026-09-26
Revision Received:	2026-10-02

Monitoring Editor: Anna Akhmanova

Scientific Editor: Dan Simon

Transaction Report:

(Note: With the exception of the correction of typographical or spelling errors that could be a source of ambiguity, letters and reports are not edited. The original formatting of letters and referee reports may not be reflected in this compilation.)

DOI: https://doi.org/10.1083/jcb.202608031

<u>Revision 0</u>

Review #1

1. Evidence, reproducibility and clarity:

Evidence, reproducibility and clarity (Required)

In this study, Stachera and colleagues investigate TRIM37 mode of action in recognizing centrosomal proteins, which leads to their ubiquitination and degradation by the proteasome. Much attention has been given in past years to TRIM37. This E3 ubiquitin ligase is associated with tumor formation and Mulibrey nanism. Past work from multiple labs has identified CEP192 and Centrobin (CENT) as centrosomal substrates . Still, we do not know much about the mechanisms at play and it is relevant to decipher how TRIM37 interacts with its substrates to promote their timely ubiquitination.

Here the authors tested how direct is TRIM37's function in promoting Cep192 ubiquitination. They identified a cluster of 7 lysines embedded in ASH domains, which are located at the C-terminus of Cep192. They show that this is essential to promote binding and ubiquitination. Further, in a very nice experimental set up they show that fusion of this IDR+ASH8 domain to a substrate that it is not normally recognized by TRIM37 results in its degradation. Finally, through the biochemical characterization of TRIM37-Cep192 complex, the authors show that IDR-ASH8 is mostly monomeric but can form higher ordered structures such dimers, trimers and tetramers. Combining alphafold structures with biochemical data allowed for fitting possible structures of ASH8 monomers with TRIM37 dimers.

This is an easy paper to read, written in a clear manner that allows the reader to go through the text and figures without much effort.

I do think that most claims and conclusions are supported by the figures. However, I note that some figures, like western blots lack quantifications or even information about how many times these experiments were performed. For example, Figures 1A, 1E and 1H. But many others.

A few things should be clarified or explained:

If I look at Figure 1A, the amount of free tubulin appears similar, is this expected? I would expect a reduction, in the +Trim37 lane, like in 1H. In 1D, the GFP pull down of GFP-EB1, how to explain that it brings TRIMP37, even if to a reduced level?

The inducible GFP-Cep192 experiments in Figure 2, they are performed in a WT background? Meaning with endogenous Cep192 present? This should be stated.

I do not understand the quantification in Fig 2C. Cep192 Foci /per image. Which type of image? Containing many cells (and so not shown)? I see very few Cep192 foci actually in the panel shown in Fig2B. It is difficult to understand the Y axis with 100 foci or even more in certain conditions.

I am not sure is good to show these images with the fire luts. But even so, how to explain that at 48hrs, 7Kto R cytoplasmic signals is decreased in one cell but not the other, while it is maintained in delta ASH8?

Still on this figure, the western blot does not show a reduction of WT cep192 at 24hrs, but

the graphs in C and D do show highly statistically significative differences in the levels and number of foci. How to interpret this?
The lengthier mitotic timing is interesting, but it should be better quantified or characterized. Can the authors show movies with a chromosome marker or if not possible DIC, to infer in cell shape changes that may inform on possible differences in mitotic duration? Do they think this lengthier mitosis accounts for decreased proliferation or there are other factors? I ask this question because the lengthier mitosis is seen in the cell line that expresses 7KtoR, but the proliferation of this cell is not as decreased as in cells expressing delta ASH8. Maybe they should actually film these cells over an entire cell cycle to take such conclusions? Otherwise they should be very careful in presenting these results together.

As a minor comment

I think the results can be better structured and should be limited to presenting the results and taking conclusions. Some data related to other studies, are presented in results and discussion. This is not necessary. They can be left for the discussion section.

2. Significance:

Significance (Required)

This is an interesting study certainly contributing for our understanding of how TRIM37 recognizes its substrates and interacts with CEP192 proteins.

3. How much time do you estimate the authors will need to complete the suggested revisions:

Estimated time to Complete Revisions (Required)
(Decision Recommendation)

Between 1 and 3 months

4. *Review Commons* values the work of reviewers and encourages them to get credit for their work. Select 'Yes' below to register your reviewing activity at [Web of Science Reviewer Recognition Service]() (formerly Publons); note that the content of your review will not be visible on Web of Science.

Web of Science Reviewer Recognition

Yes

Review #2

1. Evidence, reproducibility and clarity:

Evidence, reproducibility and clarity (Required)

In this manuscript, the authors elucidate the molecular mechanism by which TRIM37 mediates the ubiquitination and subsequent degradation of Cep192. Using mass spectrometry, they identify seven amino acid residues on Cep192 that serve as ubiquitination sites targeted by TRIM37. They further map ASH8 as a binding region within Cep192. Through an in vitro reconstitution system, the authors biochemically demonstrate that TRIM37 ubiquitinates Cep192, and that this process critically depends on both the identified ubiquitination sites and the interaction between two proteins.
In cells, expression of either an ASH8-deficient or ubiquitination-site mutants results in

stabilization of Cep192 during interphase, suggesting that TRIM37-dependent ubiquitination contributes to Cep192 turnover. During mitosis, these mutants show reduced accumulation at centrosomes, accompanied by phenotypes including prolonged mitosis and suppressed cell proliferation.

The authors then perform functional analyses of an IDR region containing ASH8 and the ubiquitination-related segment. When this region is fused to GFP-EB1, a reduction in protein abundance is observed, and this effect is rescued by siTRIM37, indicating that this region is sufficient to confer TRIM37-dependent targeting. In addition, the authors characterize the binding affinity and interaction mode between this region and TRIM37 using purified-protein binding assays and XL-MS.

Overall, this study provides the first detailed experimental evidence describing the mechanism by which TRIM37 ubiquitinates Cep192, and links this intracellular ubiquitination process to cellular phenotypes through analyses of mutant proteins. However, the downstream mechanisms explaining how this ubiquitination pathway regulates mitotic duration and cell proliferation remain insufficiently resolved.

Major Points

1. Physiological significance of ubiquitination: What is the physiological role of this ubiquitination event? Although bipolar spindle formation appears to proceed to some extent, mitosis is still prolonged-why is this the case? More specifically, which mitotic phase is delayed or arrested?
2. Discrepancy between mutant phenotypes and siTRIM37 phenotype: Why do the mitotic phenotypes of the ASH8-deficient and ubiquitination-site mutant forms of Cep192 differ from the phenotype observed upon siTRIM37? If these mutants exhibit additive phenotypes, how can this be mechanistically explained?
3. Reduced centrosomal localization in mitosis: Why do these mutants display reduced Cep192 accumulation at centrosomes during mitosis? Additionally, if examined by super-resolution microscopy, does the intra-centrosomal distribution pattern of Cep192 change?
4. Impact on PCM architecture and MTOC function: Upon expression of the ASH8 deficient and ubiquitination-site mutant forms of Cep192, how are PCM organization and microtubule-nucleation/MTOC function altered during interphase and mitosis?
5. Subcellular localization of TRIM37 across the cell cycle: Where does TRIM37 localize throughout the cell cycle, and where is Cep192 likely being degraded? The manuscript would benefit from a clearer model of the spatiotemporal site of degradation.

Minor Points

6. In Figure 1E, the IDR+ASH8 protein appears to bind TRIM37 more strongly than ASH8 alone. Does the IDR region by itself also bind to TRIM37?
7. In Figure 2B, in WT, Cep192 degradation seems more pronounced in the cytoplasm, whereas in the two mutants Cep192 appears to persist in the cytoplasm for a longer

period. It would be helpful to compare quantitative measurements of total cellular or cytoplasmic Cep192 signal. Also, where in the cell is this degradation occurring?
8. In Figure 3, mitosis is prolonged in cells expressing the two mutant proteins. What happens to these cells afterward? Do they undergo mitotic slippage or chromosome segregation errors? Quantification of these outcomes may strengthen the conclusions.
9. In Figure 5D,E, how would a full-length AF model of TRIM37 predict binding to full-length ASH8 and/or full-length Cep192? In Figure 5D, ASH8 appears proximal to multiple regions of TRIM37-this may warrant further clarification.
10. To what extent is this ubiquitination mechanism conserved across species?

2. Significance:

Significance (Required)

This manuscript is valuable in that it experimentally clarifies the previously unresolved interaction between TRIM37 and Cep192 and provides detailed insights into the ubiquitination mechanism. The work is expected to contribute meaningfully to the centrosome field and have substantial impact. On the other hand, the analysis of how this mechanism influences centrosome structure and function is not yet fully developed, and further clarification in this direction would significantly strengthen the manuscript. I am an expert of centrosome research field.

3. How much time do you estimate the authors will need to complete the suggested revisions:

Estimated time to Complete Revisions (Required)

(Decision Recommendation)

Between 1 and 3 months

4. *Review Commons* values the work of reviewers and encourages them to get credit for their work. Select 'Yes' below to register your reviewing activity at [Web of Science Reviewer Recognition Service] (formerly Publons); note that the content of your review will not be visible on Web of Science.

Web of Science Reviewer Recognition

Yes

Review #3

1. Evidence, reproducibility and clarity:

Evidence, reproducibility and clarity (Required)

Summary

Stachera et al., examine how the E3 ubiquitin-ligase TRIM37 targets the centrosomal protein CEP192 for degradation. They identify 7 C-terminal Lys residues that can be ubiquitinated by TRIM37 in vitro and identify a domain in CEP192 (IDR+ASH8) that binds to TRIM37 in a bipartite manner. The behaviour of various mutant constructs suggest that this binding mechanism is important in regulating CEP192 levels in cells. Moreover, when fused to another protein, this IDR+ASH8 domain can trigger its degradation, confirming that it can function as a degron. Importantly, the authors suggest that IDR+ASH8 is monomeric and binds to TRIM37 with relatively high-affinity, indicating a different mode of binding to that suggested in previous studies.

The findings reported here are potentially very interesting, but there are several points that the authors should consider.

Major points:

1. Two recent papers assessed how TRIM37 targets centrosomal proteins for degradation (Yeow et al., NSMB., 2025; Bellaart et al., NSMB., 2025). They both concluded that the TRAF domain of TRIM37 initially engages with substrates (either Centrobin or CEP192) through low-affinity interactions but, if the substrates are in an oligomeric form, this can trigger the formation of high-avidity TRIM37-dependent assemblies and stimulate degradation. The authors cite these papers but don't mention the TRAF domain in their analysis, making it hard for readers to assess the significance and novelty of the current work. Instead, the authors describe CEP192 binding primarily in terms of interactions with TRIM37 CC2/CC3 and the C terminal tail and suggest that the MATH domain is not involved. It took me a while to realise that the MATH domain is (or at least partially overlaps with) the TRAF domain. I'm not exactly sure how these domains are related, but this is clearly relevant, and it shouldn't be up to the reader to try to figure this out. I think a unified domain schematic aligning these domains and summarising the different ways CEP192 and Centrobin are thought interact with this module would be helpful. Out of interest, is it obvious why the previous studies identified the low-affinity TRAF interaction with CEP192 but missed the high-affinity interaction the authors report here?
2. The most interesting and novel aspect of this work is that TRIM37 can apparently bind monomeric IDR+ASH8 with high affinity and target it for destruction. Thus, in contrast to both previous reports described above, there is no need for the substrate to be already incorporated into higher order assemblies to be degraded. There are two potential issues with this that should at least be commented on.

2a. While the IDR+ASH8 domain is mostly monomeric, it can clearly form multimers in vitro (Figure 5C). Is it possible that these multimers are preferentially the target of ubiquitination/degradation in vitro? Could this effect the Kd calculation (i.e. could the multimeric forms bind more tightly than the monomers, skewing the "average" Kd)?

2b. The authors should discuss why it might be advantageous to target CEP192 monomers, as this is not obvious to me. Couldn't the mechanism they describe here still be used to preferentially target CEP192 oligomers in the cell due to the oligomerizing potential of TRIM37?
3. The important data characterising the degradation of WT and mutant forms of CEP192 by western blotting seems to rely on a single western blot that is not quantified (Figure 2E). Is this correct? This experiment should be repeated and ideally quantified.

Minor points:

1. In several places the authors make statements that I think should be modified.

Intro Para. 1: "Even modest disruptions in PCM protein levels can impair spindle assembly, promote aneuploidy, and drive tumorigenesis (REFs)." I don't think the papers they quote (or any papers for that matter) show that modest disruptions in PCM protein levels can drive tumorigenesis.

Intro Para. 2: "Whether TRIM37 targets CEP192 directly or indirectly through other pathways has not been tested." When I read this, I thought it odd as my impression is that there is strong evidence that TRIM37 binds to CEP192 and targets it for degradation. I take it the authors mean that it was possible that TRIM37 might not bind to CEP192 directly, but could bind via another protein and engage a different E3 ligase to target CEP192? Of course this was possible, but nobody thought this was likely, so I would reword.

Last sentence of Introduction: "Together, these findings reveal the molecular basis of Cep192 regulation and illustrate how a RING E3 ligase recognizes both unstructured and folded substrate elements to achieve substrate specificity." These are very grand claims. I'm sure CEP192 function is regulated in many ways (e.g., phosphorylation), and there are no actual atomic structures presented here (just domain deletions), which is what I would expect for this type of statement.

2. The observation that ubiquitin-deficient CEP192 accumulates at interphase centrosomes but is depleted from mitotic centrosomes is intriguing. Has this been observed in any previous studies? I would be interested to hear a bit more detail on the authors thinking on this point.

2. Significance:

Significance (Required)

This is the first paper to directly show that CEP192 can be ubiquitinated by TRIM37, and that ubiquitination is required CEP192 degradation. This is important but was widely anticipated.

The most interesting and novel aspect of this work is that the authors suggest a mechanism for TRIM37 degrading CEP192 that is different to the consensus mechanism that emerged from the two recently published papers discussed above. These papers were probably more substantial pieces of work and they both postulated a multimerization mechanism that drew interesting parallels with how TRIM5 degrades HIV capsids. This paper is potentially important because it suggests an alternative mechanism where substrate (CEP192) multimerization is not required to trigger degradation. This is interesting, although it remains unproven whether the TRIM37-dependent degradation of CEP192 monomers in the cytoplasm suggested here is physiologically important.

This paper will be of interest to the centrosome/mitosis field and to a more general ubiquitination/protein degradation audience.

My expertise is in centrosome and cell cycle biology.

3. How much time do you estimate the authors will need to complete the suggested revisions:

Estimated time to Complete Revisions (Required)
(Decision Recommendation)
Between 1 and 3 months
4. *Review Commons* values the work of reviewers and encourages them to get credit for their work. Select 'Yes' below to register your reviewing activity at Web of Science Reviewer Recognition Service (formerly Publons); note that the content of your review will not be visible on Web of Science.
Web of Science Reviewer Recognition
No

Reviewer #1

Evidence, reproducibility and clarity

In this study, Stachera and colleagues investigate TRIM37 mode of action in recognizing centrosomal proteins, which leads to their ubiquitination and degradation by the proteasome. Much attention has been given in past years to TRIM37. This E3 ubiquitin ligase is associated with tumor formation and Mulibrey nanism. Past work from multiple labs has identified CEP192 and Centrobin (CENT) as centrosomal substrates . Still, we do not know much about the mechanisms at play and it is relevant to decipher how TRIM37 interacts with its substrates to promote their timely ubiquitination.
Here the authors tested how direct is TRIM37's function in promoting Cep192 ubiquitination. They identified a cluster of 7 lysines embedded in ASH domains, which are located at the C-terminus of Cep192. They show that this is essential to promote binding and ubiquitination. Further, in a very nice experimental set up they show that fusion of this IDR+ASH8 domain to a substrate that it is not normally recognized by TRIM37 results in its degradation. Finally, through the biochemical characterization of TRIM37-Cep192 complex, the authors show that IDR-ASH8 is mostly monomeric but can form higher ordered structures such dimers, trimers and tetramers. Combining alphafold structures with biochemical data allowed for fitting possible structures of ASH8 monomers with TRIM37 dimers.
This is an easy paper to read, written in a clear manner that allows the reader to go through the text and figures without much effort.

I do think that most claims and conclusions are supported by the figures. However, I note that some figures, like western blots lack quantifications or even information about how many times these experiments were performed. For example, Figures 1A, 1E and 1H. But many others.

We made sure to include information about the N value and number of biological replicates in the figure legends.

A few things should be clarified or explained:
If I look at Figure 1A, the amount of free tubulin appears similar, is this expected? I would expect a reduction, in the +Trim37 lane, like in 1H.

The reviewer probably means “free ubiquitin” rather than “free tubulin”. In this experiment, we added more free ubiquitin than usual to ensure full ubiquitination and improve our mass spec identification (10 uM ubiquitin in Fig. 1A vs 0.5 uM ubiquitin in

Fig. 1H). Because of this, for Figure 1A, there may be a difference in the free ubiquitin amounts between lanes, but it is too slight to detect in this blot.

In 1D, the GFP pull down of GFP-EB1, how to explain that it brings TRIMP37, even if to a reduced level?

This interaction is likely weak or non-specific binding of TRIM37 to the GFP-trap beads or with EB1 itself. We know this because pre-blocking the beads with milk reduces this non-specific interaction. Thus, for Figure 1D, we pre-blocked the beads, then performed the pull down. We chose to show GFP-EB1 as a control to illustrate what non-specific binding looks like to emphasize the specificity of Cep192 binding.

The inducible GFP-Cep192 experiments in Figure 2, they are performed in a WT background? Meaning with endogenous Cep192 present? This should be stated.

That is correct. We have now updated the text to make this more obvious.

I do not understand the quantification in Fig 2C. Cep192 Foci /per image. Which type of image? Containing many cells (and so not shown)? I see very few Cep192 foci actually in the panel shown in Fig2B. It is difficult to understand the Y axis with 100 foci or even more in certain conditions.

The reviewer has misinterpreted the data, possibly due to our choice of data display and inadequate explanation. The images shown were cropped to highlight a few cells. The quantification was performed on much larger fields of view. We also admit that reporting "Cep192 foci per image" was improper and confusing.

In the revised version, we repeated the experiment with fixed cells and counted the number of Cep192 foci per cell per large field of view (Fig. 2F,G). Again, we show cropped images in the figure. Expression of Cep192 mutants leads to more foci that persist even after expression is turned off. Thus, our main conclusion remains the same.

F

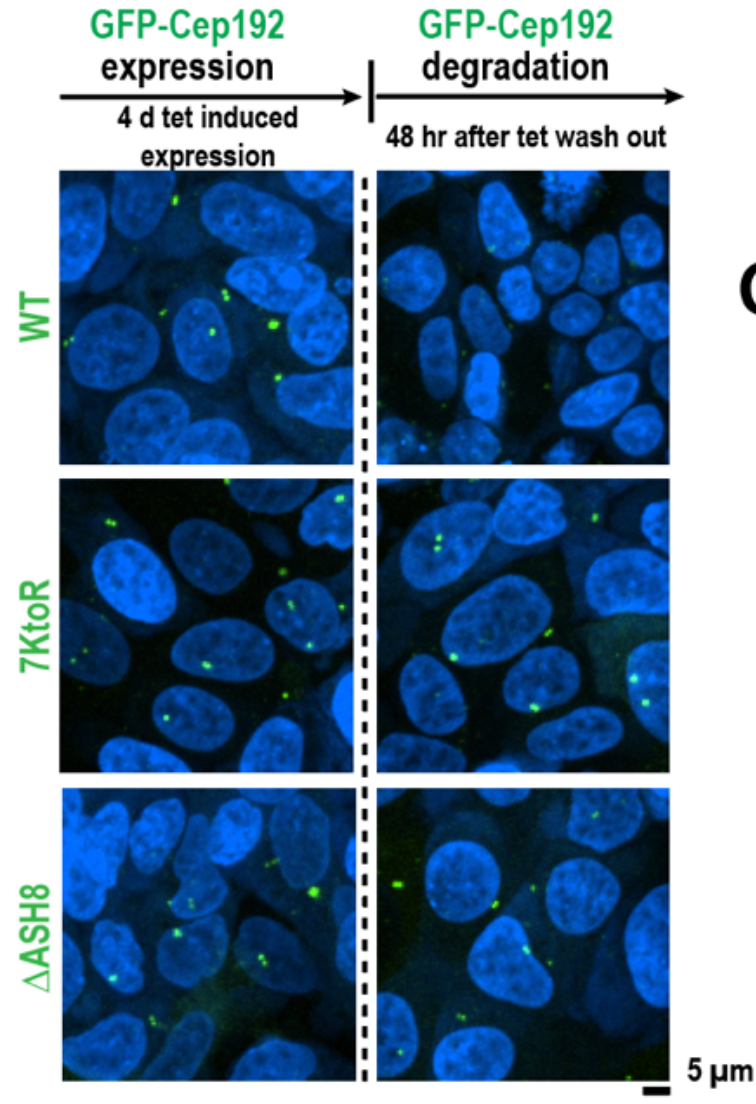

G

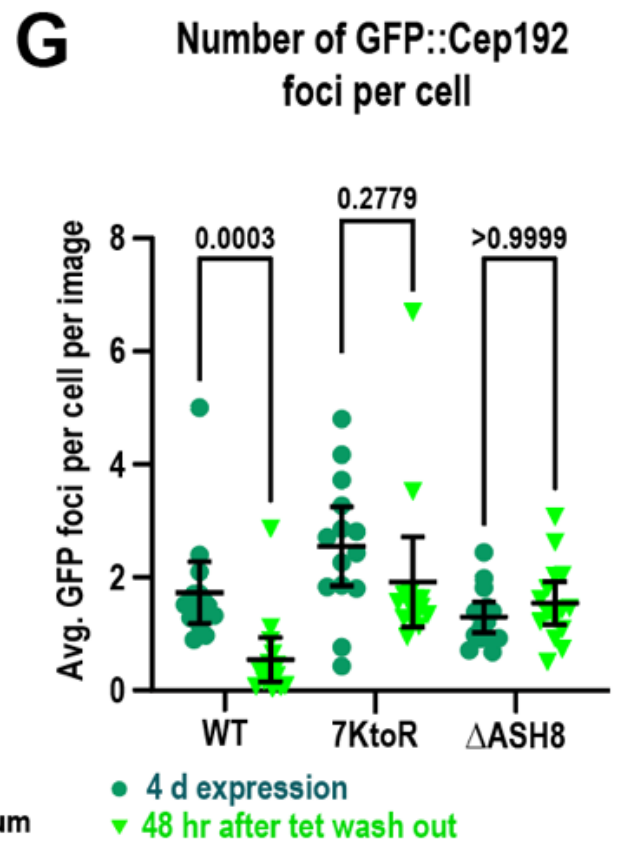

I am not sure is good to show these images with the fire luts. But even so, how to explain that at 48hrs, 7Kto R cytoplasmic signals is decreased in one cell but not the other, while it is maintained in delta ASH8?

I prefer fire LUTs because they display the range of data. Also, personally, I have a hard time discerning gradations of a single color like green.

Regarding the images, there is cell to cell variation in expression of the transgenes. To provide a better representation of the data, we quantified cytoplasmic signal (Fig. 2E). This shows that the mutant constructs persist in the cytoplasm after expression is shut off.

E

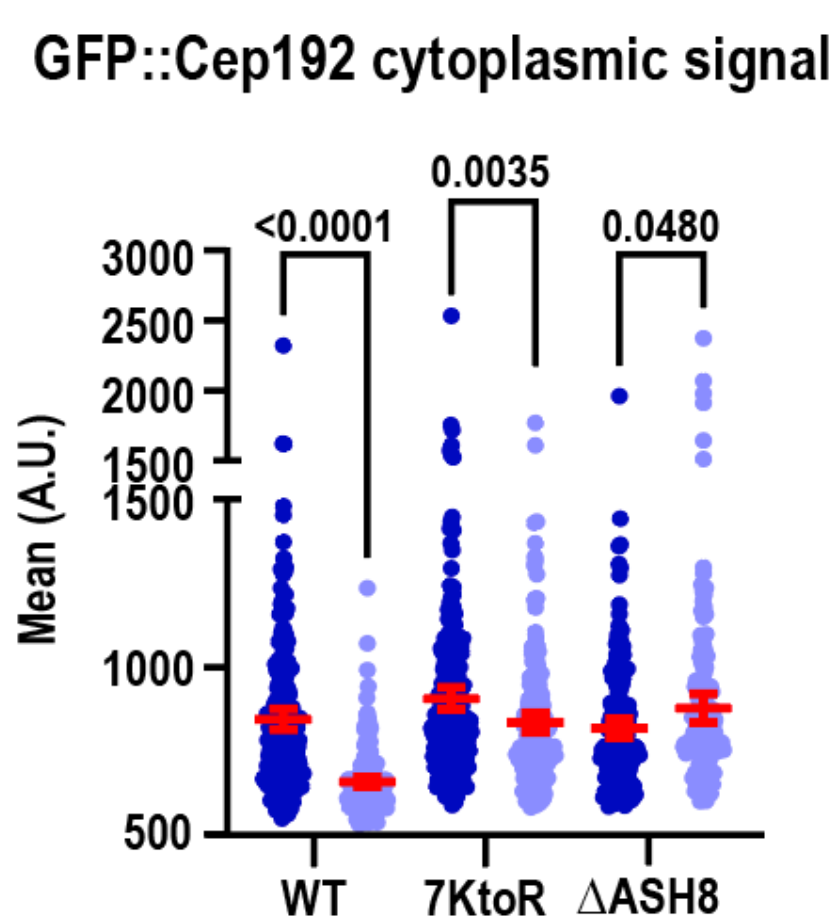

Still on this figure, the western blot does not show a reduction of WT cep192 at 24hrs, but the graphs in C and D do show highly statistically significative differences in the levels and number of foci. How to interpret this?

The graphs in Fig. 2 show data at **48 hr** following tetracycline removal, not 24 hr. We include a label explain this below Fig 2D and describe it in the figure legend. This result is consistent with our western blots, which are now quantified in triplicate (see revised Fig. S1J,K).

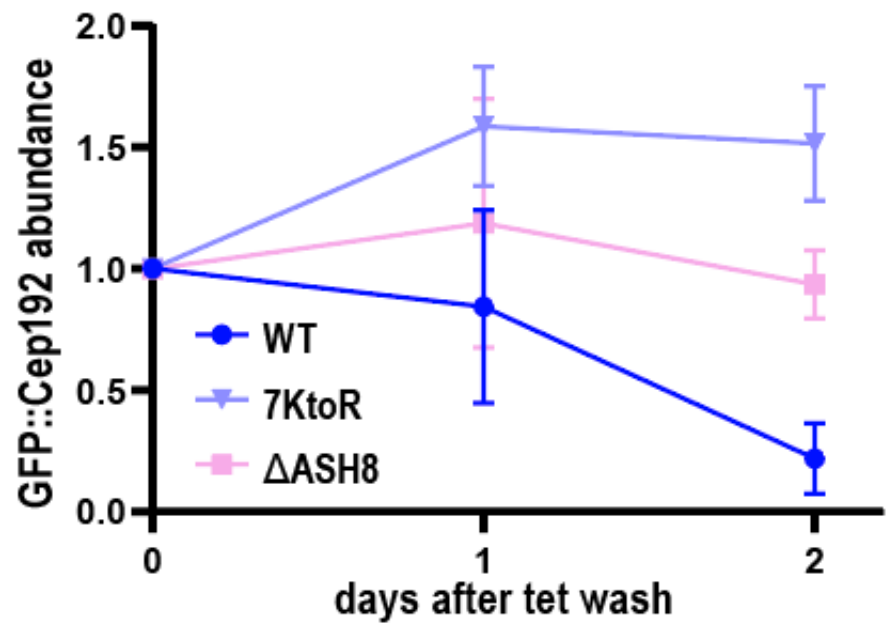

The lengthier mitotic timing is interesting, but it should be better quantified or characterized. Can the authors show movies with a chromosome marker or if not possible DIC, to infer in cell shape changes that may inform on possible differences in mitotic duration? Do they think this lengthier mitosis accounts for decreased proliferation or there are other factors? I ask this question because the lengthier mitosis is seen in the cell line that expresses 7KtoR, but the proliferation of this cell is not as decreased as in cells expressing delta ASH8. Maybe they should actually film these cells over an entire cell cycle to take such conclusions? Otherwise they should be very careful in presenting these results together.

In the revised version, we characterized this phenomenon better. First, we used DIC imaging over 20 hr to monitor cell cycle length and fate. Second, we synchronized cells in mitosis, release them, and measured the percentage of cells still in mitosis over time. Third, we use super-resolution imaging (SoRa) to monitor spindle assembly and morphology and centrosome morphology. The results are included in the revised Fig. 3 and S2. Our data suggest that over-expression of the Cep192 mutant constructs causes defects in centrosome and spindle morphology, which arrests some cells in mitosis and leads to cell death. In each metric, there are differences between the 7KtoR and ΔASH8 mutants, which we cannot fully explain at this time. Cep192(7KtoR) had a stronger effect on centrosome morphology, while ΔASH8 had a stronger effect on spindle density. We cannot rule out that either mutation blocks just ubiquitination; the binding of other proteins could certainly be affected.

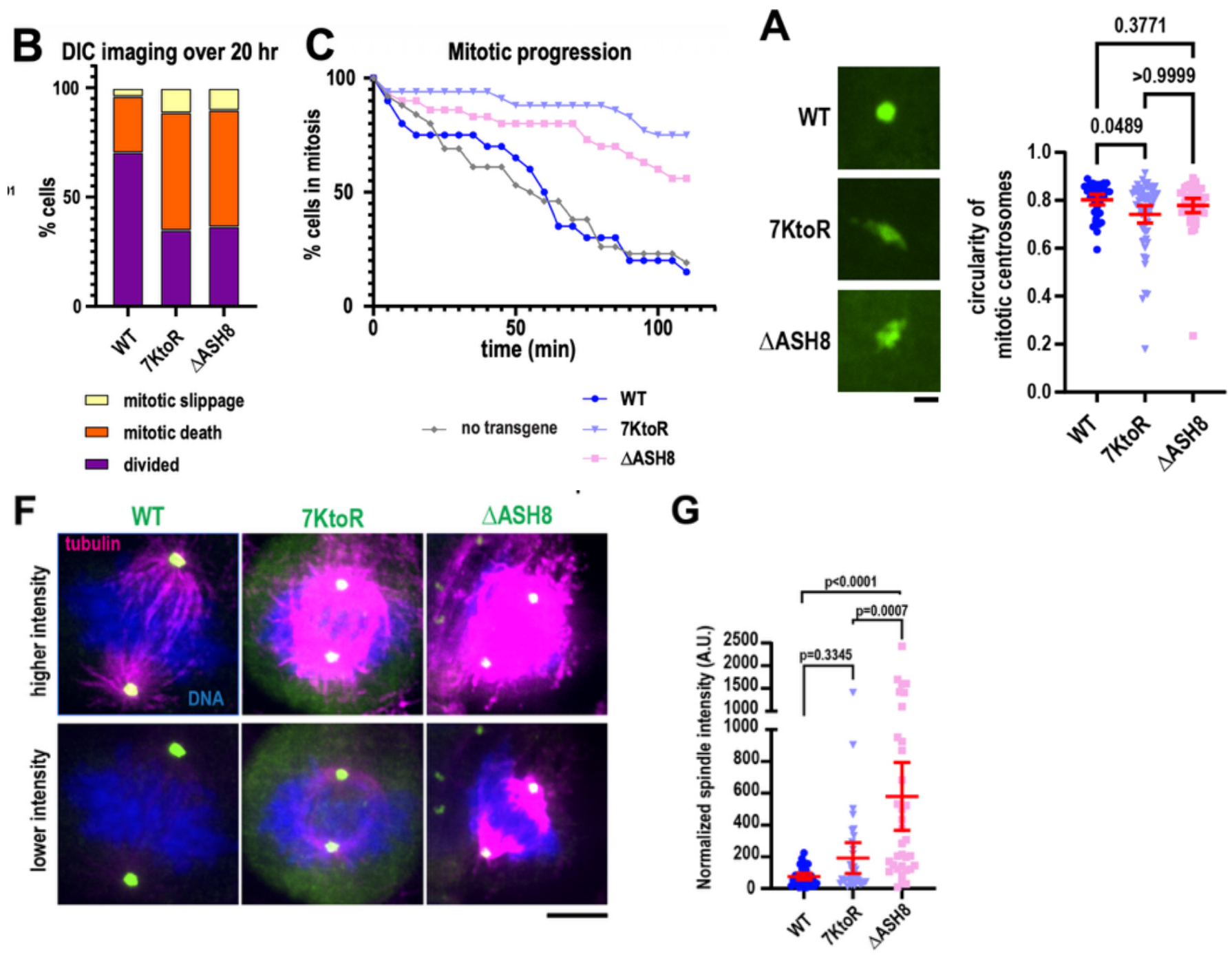

As a minor comment
I think the results can be better structured and should be limited to presenting the results and taking conclusions. Some data related to other studies, are presented in results and discussion. This is not necessary. They can be left for the discussion section.

We now have removed comments regarding other studies (e.g., comments on the TRIM37-binding affinity with centrobin) and focus only on our results and conclusions.

Significance

This is an interesting study certainly contributing for our understanding of how TRIM37 recognizes its substrates and interacts with CEP192 proteins.

Reviewer #2

Evidence, reproducibility and clarity

In this manuscript, the authors elucidate the molecular mechanism by which TRIM37 mediates the ubiquitination and subsequent degradation of Cep192. Using mass spectrometry, they identify seven amino acid residues on Cep192 that serve as ubiquitination sites targeted by TRIM37. They further map ASH8 as a binding region within Cep192. Through an in vitro reconstitution system, the authors biochemically demonstrate that TRIM37 ubiquitinates Cep192, and that this process critically depends on both the identified ubiquitination sites and the interaction between two proteins.

In cells, expression of either an ASH8-deficient or ubiquitination-site mutants results in stabilization of Cep192 during interphase, suggesting that TRIM37-dependent ubiquitination contributes to Cep192 turnover. During mitosis, these mutants show reduced accumulation at centrosomes, accompanied by phenotypes including prolonged mitosis and suppressed cell proliferation.
The authors then perform functional analyses of an IDR region containing ASH8 and the ubiquitination-related segment. When this region is fused to GFP-EB1, a reduction in protein abundance is observed, and this effect is rescued by siTRIM37, indicating that this region is sufficient to confer TRIM37-dependent targeting. In addition, the authors characterize the binding affinity and interaction mode between this region and TRIM37 using purified-protein binding assays and XL-MS.

Overall, this study provides the first detailed experimental evidence describing the mechanism by which TRIM37 ubiquitinates Cep192, and links this intracellular ubiquitination process to cellular phenotypes through analyses of mutant proteins. However, the downstream mechanisms explaining how this ubiquitination pathway regulates mitotic duration and cell proliferation remain insufficiently resolved.

Major Points

1. Physiological significance of ubiquitination: What is the physiological role of this ubiquitination event? Although bipolar spindle formation appears to proceed to some extent, mitosis is still prolonged-why is this the case? More specifically, which mitotic phase is delayed or arrested?

In the revised version, we characterized this phenomenon better. We synchronized cells in late G2, released them, then tracked cells once they entered mitosis. In the revised Fig. 3C, we now show the percentage of cells still in mitosis over time. These data support our original conclusion that over-expression of degradation-resistant Cep192, but not wild-type Cep192, alters mitotic progression. The live-cell imaging suggests they are stuck in metaphase, as chromosomes (area of excluded fluorescence) become aligned on the metaphase plate. SoRa imaging of fixed cells stained with tubulin antibodies and DAPI also show that the mitotic spindles are denser and look abnormal, yet chromosomes are aligned on the metaphase plate.

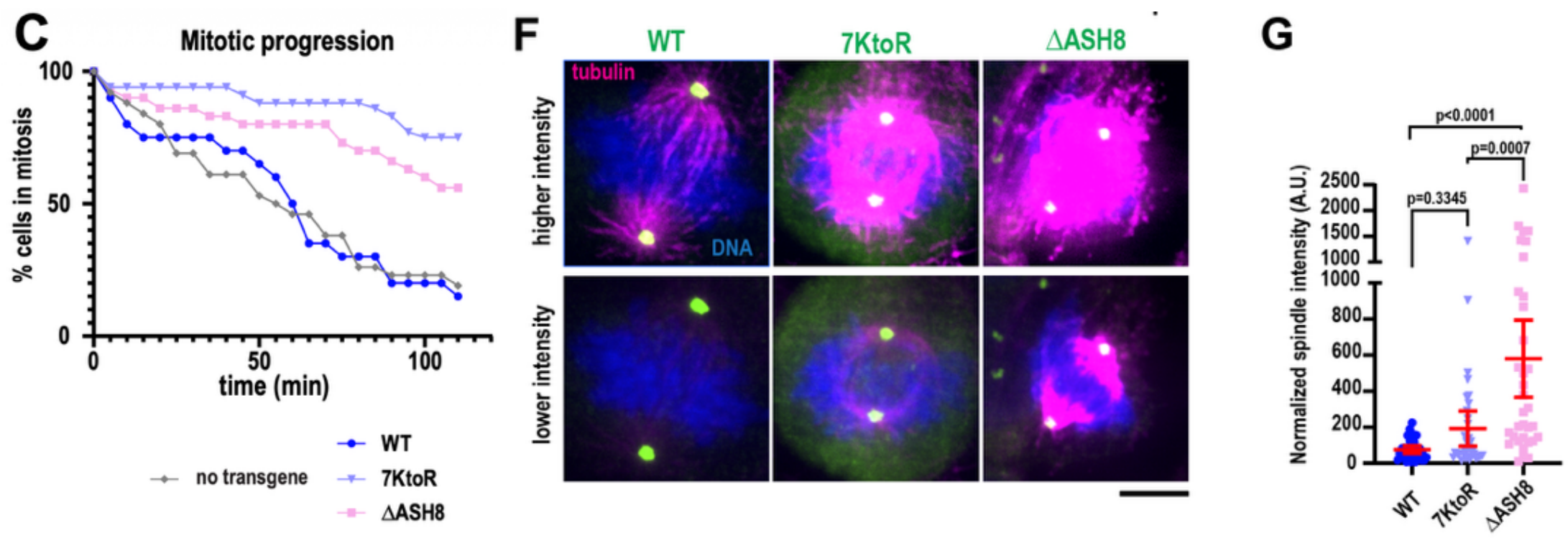

2. Discrepancy between mutant phenotypes and siTRIM37 phenotype: Why do the mitotic phenotypes of the ASH8-deficient and ubiquitination-site mutant forms of Cep192 differ from the phenotype observed upon siTRIM37? If these mutants exhibit additive phenotypes, how can this be mechanistically explained?

In our revised Figure S1H,I, we show that siTRIM37 blocks degradation of GFP-Cep192(WT), just like what we saw for the Cep192 mutants in Figure 2.

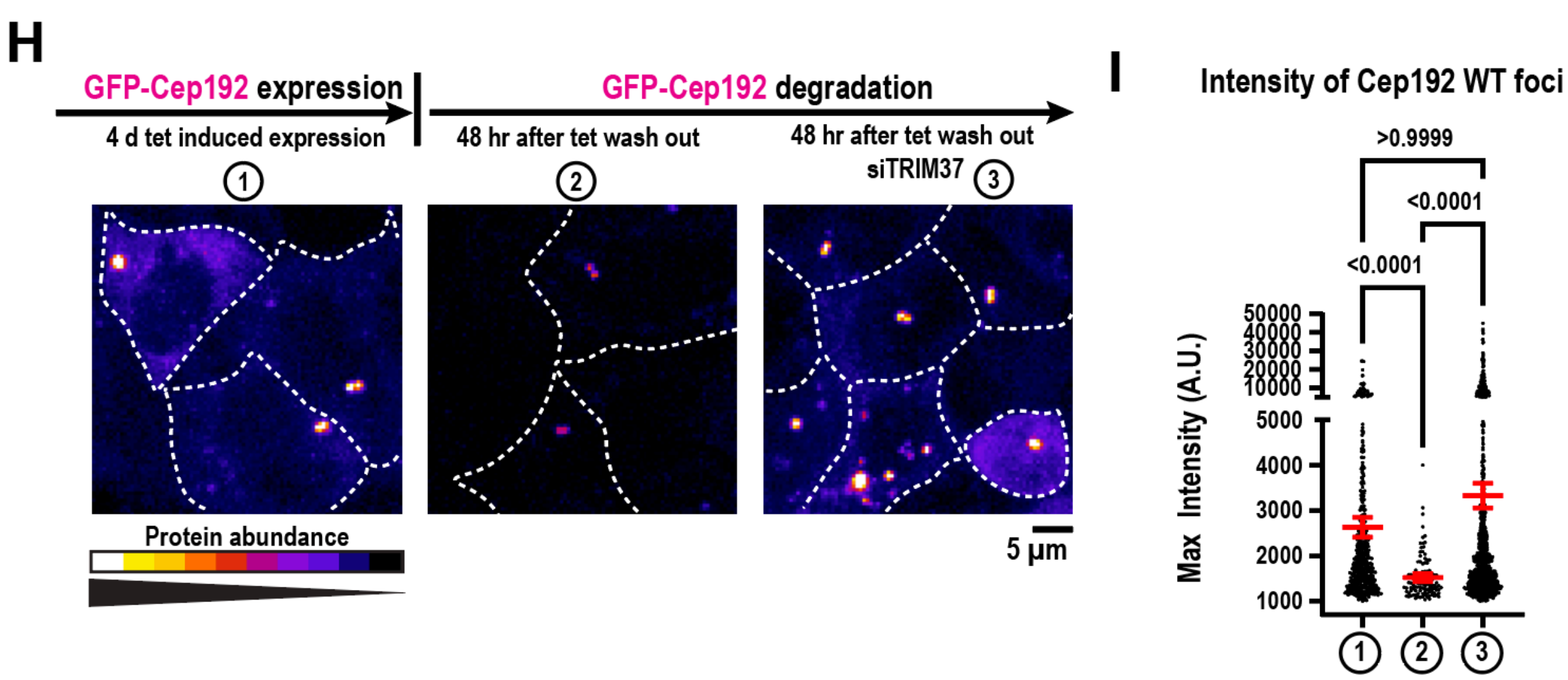

However, we did not observe extended mitosis seen before in TRIM37-depleted cells, rather mitotic arrest in the majority of our mutant cells.

We mentioned this in the Discussion:

“Overexpression of both Cep192 mutants (7KtoR and ΔASH8), but not Cep192(WT), caused cell cycle arrest in metaphase and eventually death. The molecular cause of this phenotype is unclear. Extended mitosis was observed in TRIM37-depleted cells (Meitinger et al., 2021), but this phenotype was rescued by Centrobin depletion,

suggesting that excessive accumulation of Cep192 was not the direct cause of cell cycle arrest."

We also saw impairment of PCM and spindle morphology. We do not have a complete molecular understanding yet.

We must note that our experiments differ importantly from previous studies. First, we are over-expressing mutant transgenes. It is possible that this represents an "orphan quality control" event that does not happen when simply silencing TRIM37. Here, Cep192 may be out of stoichiometric balance with some partner, which could activate degradation of Cep192(WT) but not the mutants. Second, the mutations are specific alterations to Cep192, which could, in theory, affect more than just ubiquitination. We provide a caveat in the discussion on this point.

Alternatively, ubiquitination could serve as a post-translational modification that is needed for mitotic PCM maturation and mitotic progression. We speculate on these points in the Discussion. However, we believe that this line of inquiry is beyond our main goal in identifying how TRIM37 recognizes Cep192.

3. Reduced centrosomal localization in mitosis: Why do these mutants display reduced Cep192 accumulation at centrosomes during mitosis? Additionally, if examined by super-resolution microscopy, does the intra-centrosomal distribution pattern of Cep192 change?

We are not sure why mutant Cep192 accumulates fine at interphase centrosomes but not at mitotic centrosomes. There could be some change in the Cep192 assembly mechanism that requires ubiquitination (perhaps as post-translational modification rather than a degradative signal) or an intact C-terminus. We believe that this is an interesting finding; however, getting mechanistic insight into this phenomenon is beyond the scope of the current study.

Following the reviewer's advice, we used super-resolution imaging (SoRa) to monitor centrosome morphology (Fig. S2A). Our data suggest that over-expression of the Cep192 mutant constructs causes centrosome disorganization. We provide quantification of circularity, which differs between the mutants and WT (however, when using a strict non-parametric multiple comparisons test, the difference is not statistically significant between ∆ASH8 and WT). We still do not know why these mutations cause alterations in centrosome structure.

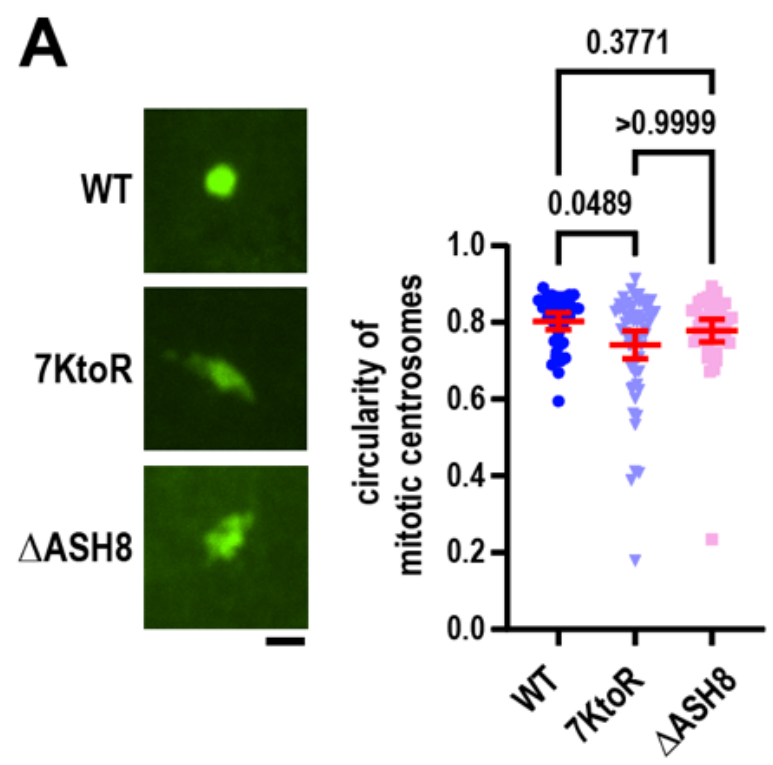

4. Impact on PCM architecture and MTOC function: Upon expression of the ASH8 deficient and ubiquitination-site mutant forms of Cep192, how are PCM organization and microtubule-nucleation/MTOC function altered during interphase and mitosis?

In our revised Fig. 2A, we show that all transgenes localized to centrosomes, as indicated by centrin staining. In Fig. 3, we used SoRa imaging to illustrate that over-expression of the mutant proteins unexpectedly caused excessive microtubule nucleation at PCM and altered spindle density. Such aberrant spindle density, combined with aberrant centrosome morphology (see above), could explain why these transgenic cells arrest in mitosis.

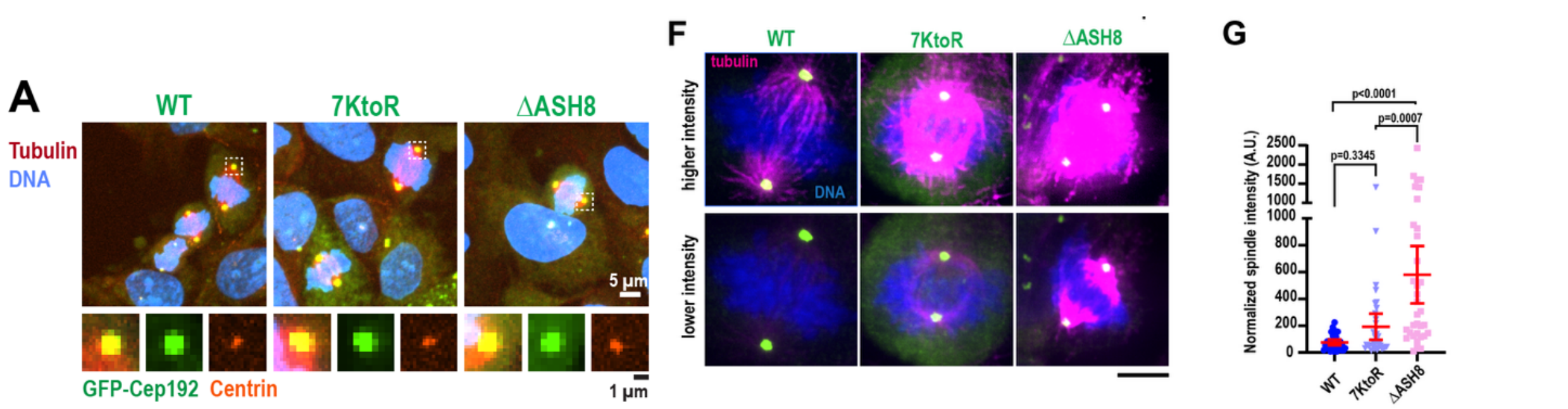

5. Subcellular localization of TRIM37 across the cell cycle: Where does TRIM37 localize throughout the cell cycle, and where is Cep192 likely being degraded? The manuscript would benefit from a clearer model of the spatiotemporal site of degradation.

Localization of TRIM37 was previously described (Meitinger et al., 2021; Yeow et al., 2025; see embedded figure below). TRIM37 localizes to centrosomes primarily in metaphase and anaphase, suggesting a role for driving PCM disassembly. In our revised Fig 2H, we show that TRIM37 is required for PCM disassembly upon cell cycle exit. Thus, TRIM37 likely ubiquitinates PCM-associated proteins at anaphase to promote PCM disassembly.

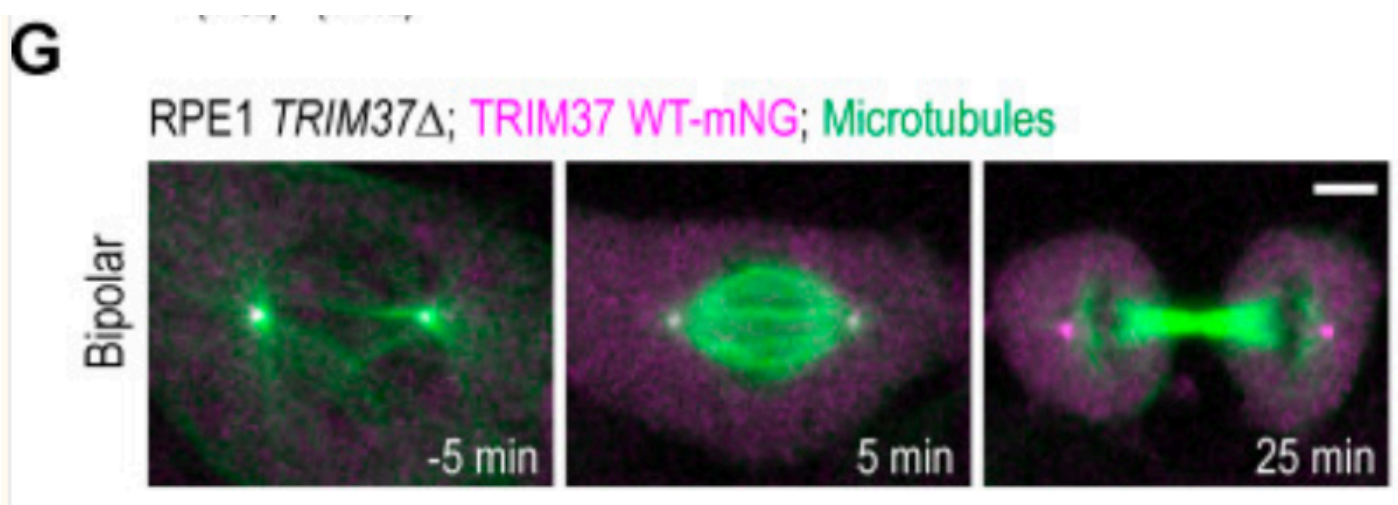

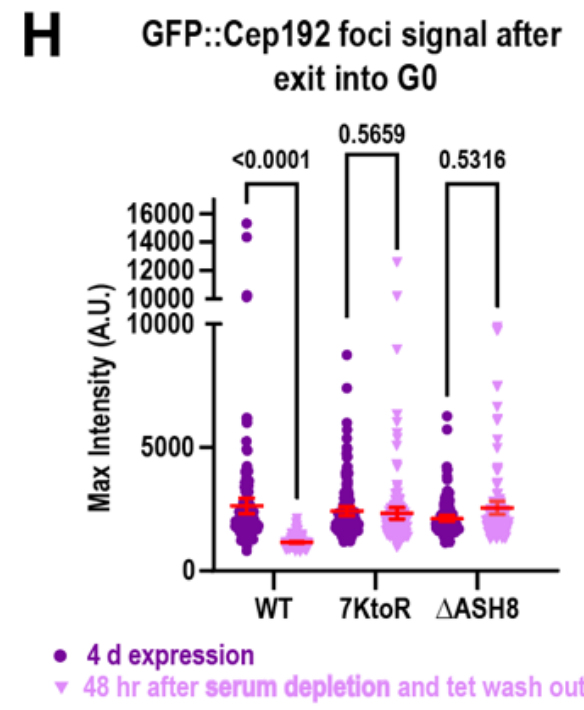

Furthermore, TRIM37 localizes to the cytoplasm in all cell cycle stages. In our revised manuscript, we demonstrate the ability of TRIM37 to bind and degrade both centrosome-localized proteins (Cep192 and GFP-EB1-IDR+ASH8)(Fig. 3) and non-centrosome-localized targets (GFP-IDR+ASH8) (Revised Fig. S3B,C). Therefore, TRIM37 is active at centrosomes and in the cytoplasm. We speculate that the cytoplasmic role is likely not restricted to a cell cycle stage and is used primarily to regulate baseline levels of proteins.

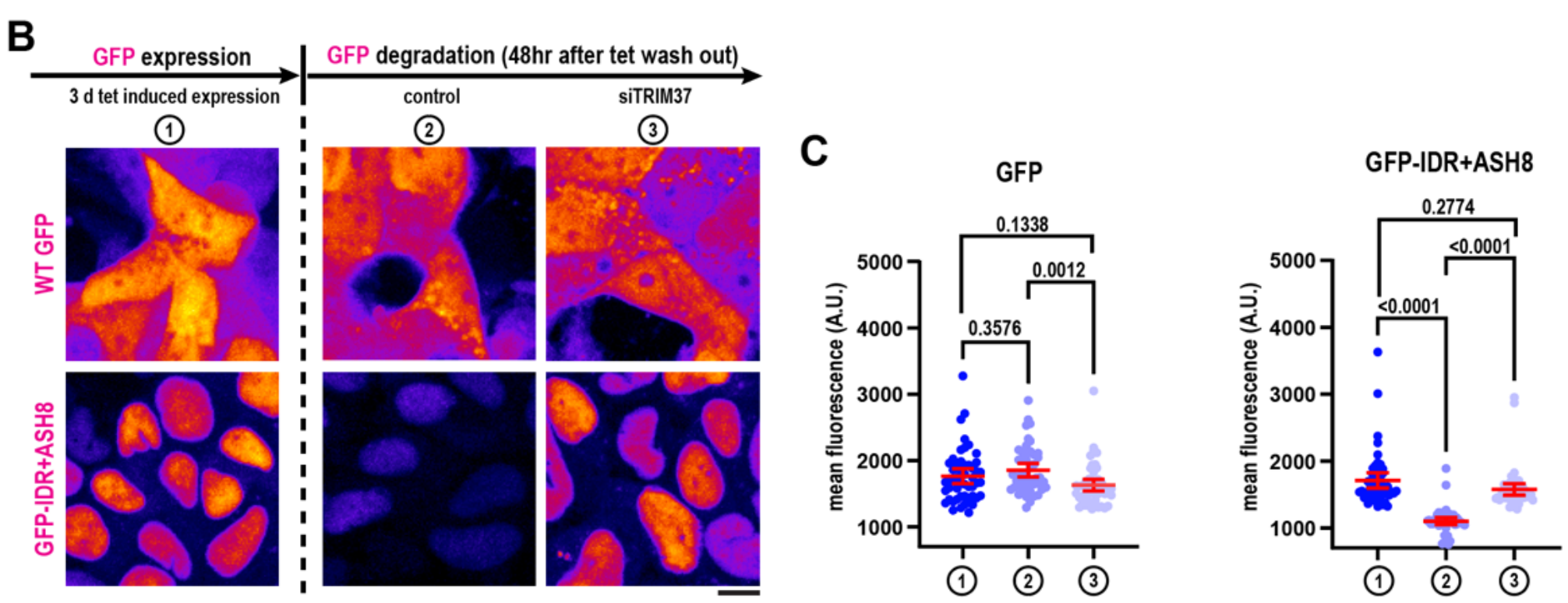

Minor Points

6. In Figure 1E, the IDR+ASH8 protein appears to bind TRIM37 more strongly than ASH8 alone. Does the IDR region by itself also bind to TRIM37?

We have repeated this experiment using just the IDR. As shown in Fig. S1D, the IDR region is not sufficient to bind TRIM37. This supports our claim that the binding motif is

bipartite, requiring both the IDR and ASH8.

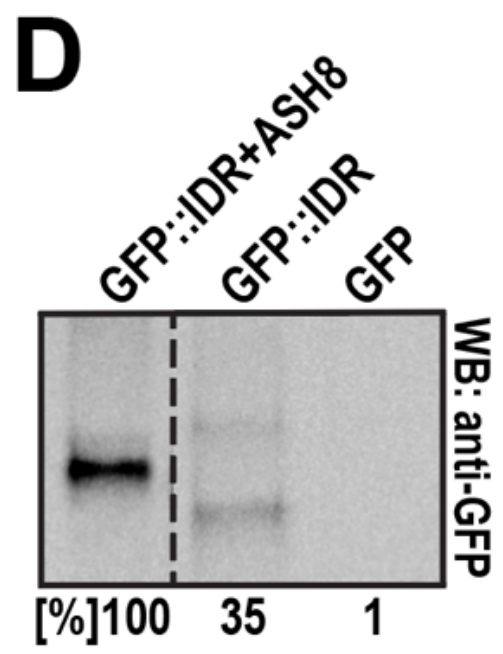

7. In Figure 2B, in WT, Cep192 degradation seems more pronounced in the cytoplasm, whereas in the two mutants Cep192 appears to persist in the cytoplasm for a longer period. It would be helpful to compare quantitative measurements of total cellular or cytoplasmic Cep192 signal. Also, where in the cell is this degradation occurring?

We now have measured cytoplasmic fluorescent signal of the Cep192 variants. After silencing transgene expression, cytoplasmic signal decreased dramatically for Cep192(WT), similar to the decline in centrosome signal (Fig. 2D,E). However, the cytoplasmic signal remained elevated for the Cep192 mutants. Total cellular levels are quantified by western blot (Figure S1J,K).

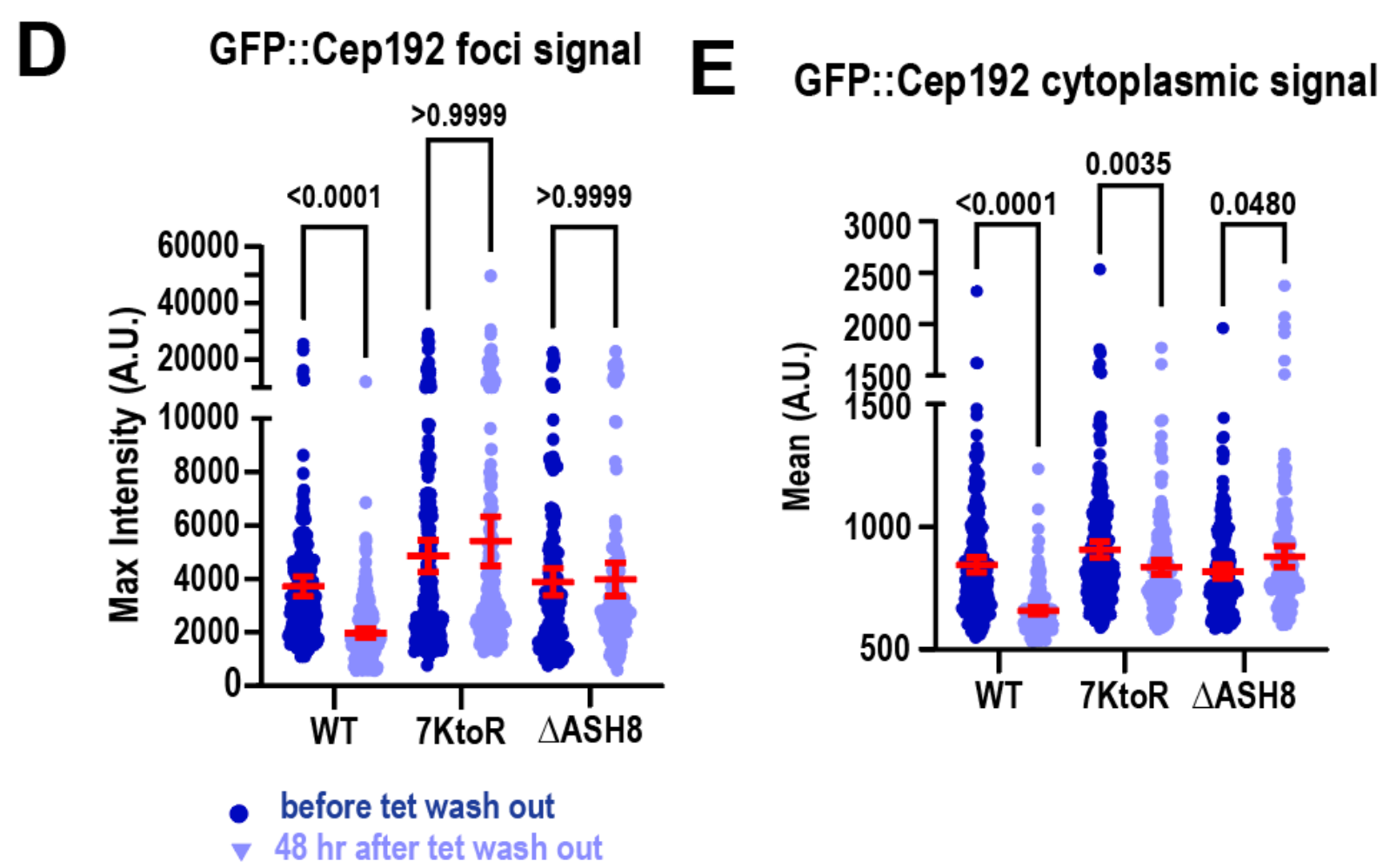

8. In Figure 3, mitosis is prolonged in cells expressing the two mutant proteins. What happens to these cells afterward? Do they undergo mitotic slippage or chromosome segregation errors? Quantification of these outcomes may strengthen the conclusions.

We imaged the cells over 20 hrs using DIC and found that many of the cells remain arrested in mitosis, then die. Only a small percentage undergo mitotic slippage.

9. In Figure 5D,E, how would a full-length AF model of TRIM37 predict binding to full-length ASH8 and/or full-length Cep192? In Figure 5D, ASH8 appears proximal to

multiple regions of TRIM37-this may warrant further clarification.

We have tried modeling full-length TRIM37 with IDR+ASH8, but the confidence is so low (ipTM and pTM <0.4) that the result is meaningless. Even models of TRIM37 fragments (e.g., a.a. 1-458 of TRIM37, which excludes the long tail) with IDR+ASH8 are too low in confidence to report.

The cartoon model in Figure 5D is based upon the combination of our XL-MS and pull-down experiments which shows an interaction between the CC2 and CC3 domains of TRIM37 with IDR+ASH8. We now label those domains on the figure.

10. To what extent is this ubiquitination mechanism conserved across species?

There is strong sequence conservation of ASH8 and the TRIM37 docking domains (CC2 and CC3) across mammals. The conservation declines somewhat in Zebrafish. TRIM37 is not found outside of vertebrates. Thus, we speculate that this ubiquitination mechanism is conserved among mammals and possibly among vertebrates**.** In our revised Fig. S4G, we show sequence alignments for these regions.

We also state in the discussion,

“This mechanism is likely active in other species, since IDR+ASH8 and the coiled-coil recognition motifs of TRIM37 are well conserved amongst vertebrates (Figure S4G).”

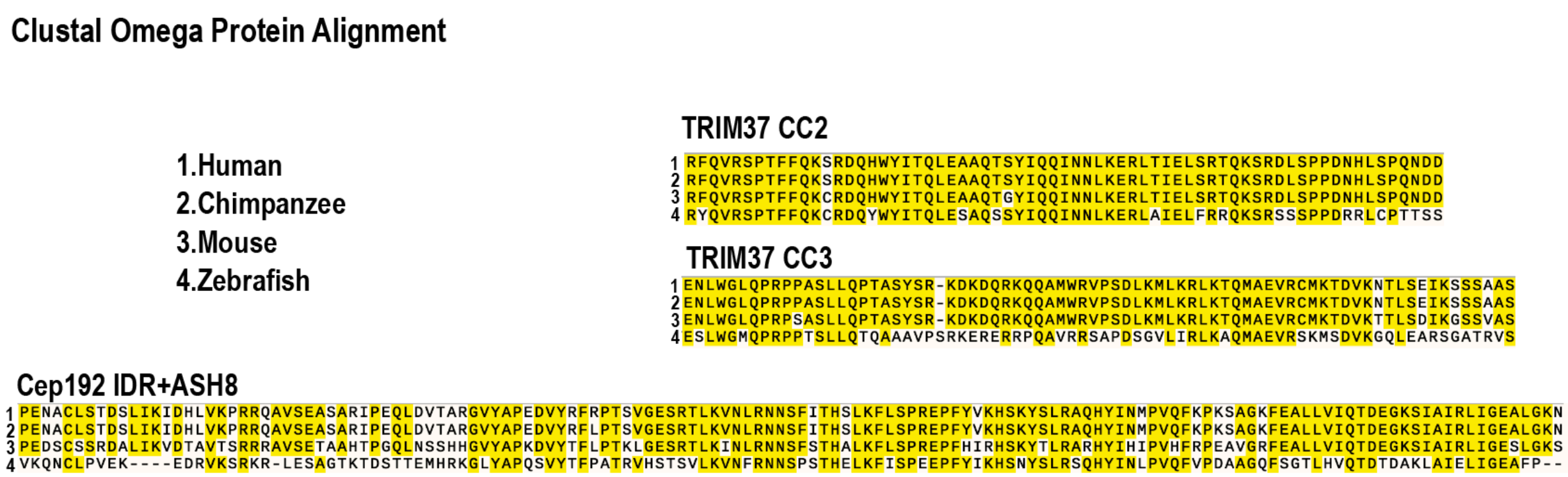

Significance

This manuscript is valuable in that it experimentally clarifies the previously unresolved interaction between TRIM37 and Cep192 and provides detailed insights into the ubiquitination mechanism. The work is expected to contribute meaningfully to the centrosome field and have substantial impact. On the other hand, the analysis of how this mechanism influences centrosome structure and function is not yet fully developed, and further clarification in this direction would significantly strengthen the manuscript. I am an expert of centrosome research field.

Reviewer #3 (Evidence, reproducibility and clarity (Required)):

Summary
Stachera et al., examine how the E3 ubiquitin-ligase TRIM37 targets the centrosomal protein CEP192 for degradation. They identify 7 C-terminal Lys residues that can be ubiquitinated by TRIM37 in vitro and identify a domain in CEP192 (IDR+ASH8) that binds to TRIM37 in a bipartite manner. The behaviour of various mutant constructs suggest that this binding mechanism is important in regulating CEP192 levels in cells. Moreover, when fused to another protein, this IDR+ASH8 domain can trigger its degradation, confirming that it can function as a degron. Importantly, the authors suggest that IDR+ASH8 is monomeric and binds to TRIM37 with relatively high-affinity, indicating a different mode of binding to that suggested in previous studies.

The findings reported here are potentially very interesting, but there are several points that the authors should consider.

Major points:

1. Two recent papers assessed how TRIM37 targets centrosomal proteins for degradation (Yeow et al., NSMB., 2025; Bellaart et al., NSMB., 2025). They both concluded that the TRAF domain of TRIM37 initially engages with substrates (either Centrobin or CEP192) through low-affinity interactions but, if the substrates are in an oligomeric form, this can trigger the formation of high-avidity TRIM37-dependent assemblies and stimulate degradation. The authors cite these papers but don't mention the TRAF domain in their analysis, making it hard for readers to assess the significance and novelty of the current work. Instead, the authors describe CEP192 binding primarily in terms of interactions with TRIM37 CC2/CC3 and the C terminal tail and suggest that the MATH domain is not involved. It took me a while to realise that the MATH domain is (or at least partially overlaps with) the TRAF domain. I'm not exactly sure how these domains are related, but this is clearly relevant, and it shouldn't be up to the reader to try to figure this out. I think a unified domain schematic aligning these domains and summarising the different ways CEP192 and Centrobin are thought interact with this module would be helpful. Out of interest, is it obvious why the previous studies identified the low-affinity TRAF interaction with CEP192 but missed the high-affinity interaction the authors report here?

The reviewer may be confused. Bellaart et al., 2025 showed that the TRAF domain of TRIM37 can bind a peptide from Centrobin with 14 uM affinity. No study has ever

biochemically reconstituted the interaction between TRIM37 and Cep192 in vitro or measured their affinity.

Furthermore, Yeow et al., 2025 showed that a mutation in the MATH domain (G322V) hinders CEP192 degradation in cells, but this could be because this mutation prevents TRIM37 dimerization (shown in that study; dimerization is needed for its ligase activity). In essence, the G322V mutation is a ligase-dead mutation; it’s not clear if this mutation directly hinders substrate binding. Thus, prior to our study, no one understood how TRIM37 binds with Cep192 or how strong the interaction is.

Also, we have updated Figure S1A to illustrate that the TRAF domain is a composite of CC2, MATH, and CC3 domains within TRIM37. The MATH domain is a more broadly recognized domain class. From a structural point-of-view, I believe it is clearer and more specific to refer to these individual modules rather than grouping them together.

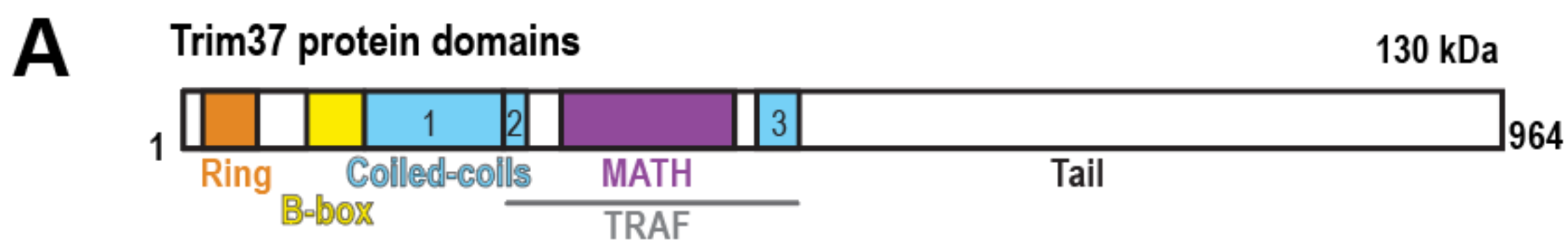

2. The most interesting and novel aspect of this work is that TRIM37 can apparently bind monomeric IDR+ASH8 with high affinity and target it for destruction. Thus, in contrast to both previous reports described above, there is no need for the substrate to be already incorporated into higher order assemblies to be degraded. There are two potential issues with this that should at least be commented on.

2a. While the IDR+ASH8 domain is mostly monomeric, it can clearly form multimers in vitro (Figure 5C). Is it possible that these multimers are preferentially the target of ubiquitination/degradation in vitro? Could this effect the Kd calculation (i.e. could the multimeric forms bind more tightly than the monomers, skewing the "average" Kd)?

It is possible. However, the monomeric species is dominant. The trimer and tetramer fractions make up <5% of the total. Our point was that a non-multimer could be targeted. We wanted to contrast our data with previous studies suggesting that TRIM family proteins only target supramolecular assemblies (e.g., micron-scale Centrobin condensates or HIV capsids).

2b. The authors should discuss why it might be advantageous to target CEP192 monomers, as this is not obvious to me. Couldn't the mechanism they describe here still

be used to preferentially target CEP192 oligomers in the cell due to the oligomerizing potential of TRIM37?

We speculate that TRIM37 uses this high-affinity binding to degrade proteins in the cytoplasm, before their incorporation into the centrosome. We further demonstrate this point by showing that GFP-IDR+ASH8 (which doesn't localize at centrosomes) gets degraded by TRIM37 in cells (Fig. S3B,C). This could be advantageous in regulating baseline levels of proteins.

Our results, however, do not exclude the possibility that TRIM37 binds Cep192 oligomers, which likely occur at the centrosome. Oligomerization should make the reaction more efficient through avidity. We simply wanted to make the point that higher-order oligomerization is not essential for TRIM37 to recognize a substrate. The previous studies (Yeow et al., 2025 and Bellaart et al., 2025) concluded that TRIM37 only acts on supramolecular oligomers (e.g., Centrobin condensates), drawing similarities to TRIM5 recognition of HIV capsids. Our data emphasize that TRIM37 has two modes of substrate recognition, one for large-scale assemblies and one for monomers or small protein complexes. We have elaborated this point in the discussion.

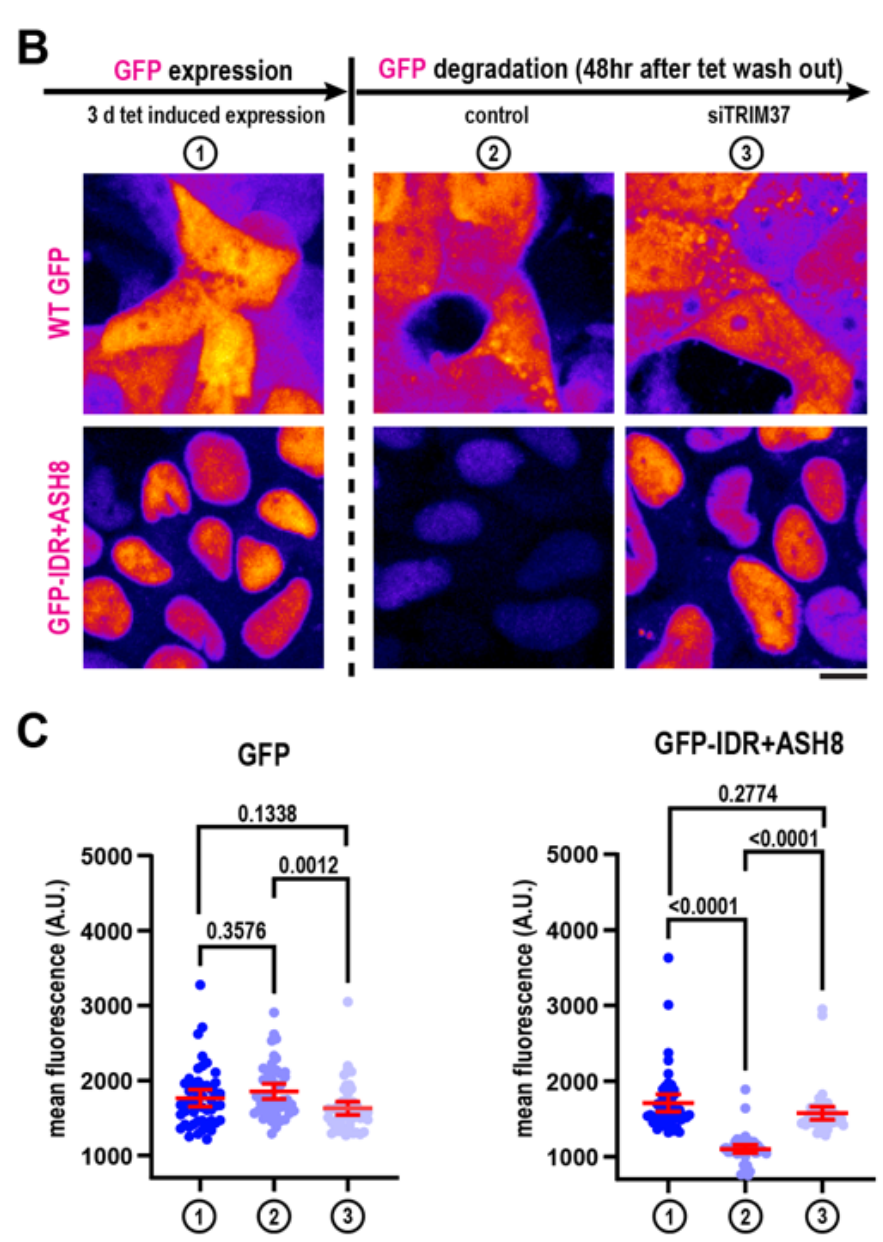

3. The important data characterising the degradation of WT and mutant forms of CEP192 by western blotting seems to rely on a single western blot that is not quantified (Figure 2E). Is this correct? This experiment should be repeated and ideally quantified.

We have repeated this experiment and show the quantification in Fig. S1K. Our

conclusion remains the same. Please note that the live-cell imaging experiments also support this conclusion.

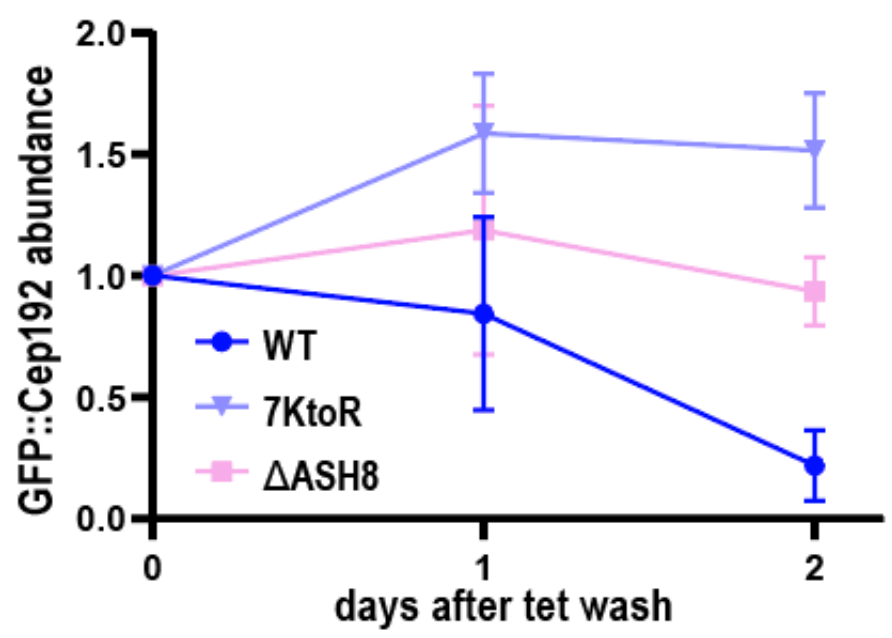

Minor points:

1. In several places the authors make statements that I think should be modified.

Intro Para. 1: "Even modest disruptions in PCM protein levels can impair spindle assembly, promote aneuploidy, and drive tumorigenesis (REFs)." I don't think the papers they quote (or any papers for that matter) show that modest disruptions in PCM protein levels can drive tumorigenesis.

This a good point, and we have revised the text as suggested.

Intro Para. 2: "Whether TRIM37 targets CEP192 directly or indirectly through other pathways has not been tested." When I read this, I thought it odd as my impression is that there is strong evidence that TRIM37 binds to CEP192 and targets it for degradation. I take it the authors mean that it was possible that TRIM37 might not bind to CEP192 directly, but could bind via another protein and engage a different E3 ligase to target CEP192? Of course this was possible, but nobody thought this was likely, so I would reword.

We have re-worded this section:

“Despite literature demonstrating a functional dependency between the two proteins, the evidence confirming that TRIM37 binds to Cep192 directly is still missing. Furthermore, the molecular mechanism underlying Cep192 recognition and ubiquitination by E3 ligases remains unknown.”

Last sentence of Introduction: "Together, these findings reveal the molecular basis of Cep192 regulation and illustrate how a RING E3 ligase recognizes both unstructured and folded substrate elements to achieve substrate specificity." These are very grand claims. I'm sure CEP192 function is regulated in many ways (e.g., phosphorylation), and there are no actual atomic structures presented here (just domain deletions), which is what I would expect for this type of statement.

We agree and have eliminated this statement.

2. The observation that ubiquitin-deficient CEP192 accumulates at interphase centrosomes but is depleted from mitotic centrosomes is intriguing. Has this been observed in any previous studies? I would be interested to hear a bit more detail on the authors thinking on this point.

To our knowledge, this result has not been reported before. We do not understand the molecular basis of this phenomenon yet. There could be some change in the Cep192 assembly mechanism that requires ubiquitination (perhaps as post-translational modification rather than a degradative signal) or an intact C-terminus. We added some speculation in the discussion:

"Although unexpected, these findings raise the intriguing possibility that Cep192 ubiquitination acts as an important post-translational signal that supports correct PCM maturation during mitosis. This could impact cell proliferation given that mitotic PCM assembly affects the speed and fidelity of mitotic division in many species (Cabral et al., 2019; Conduit et al., 2014; Hamill et al., 2002; Lawo et al., 2012; Meitinger et al., 2020). These data align with earlier reports that observed increased levels of ubiquitinated proteins at the centrosome in metaphase relative to S (Kimura et al., 2014). However, we must express caution in interpreting these results, as they come from cells over-expressing transgenes. Together, these observations point to regulatory layers that extend beyond the scope of the present study and warrant further investigation."

Reviewer #3 (Significance (Required)):

This is the first paper to directly show that CEP192 can be ubiquitinated by TRIM37, and that ubiquitination is required CEP192 degradation. This is important but was widely anticipated.

The most interesting and novel aspect of this work is that the authors suggest a mechanism for TRIM37 degrading CEP192 that is different to the consensus mechanism that emerged from the

two recently published papers discussed above. These papers were probably more substantial pieces of work and they both postulated a multimerization mechanism that drew interesting parallels with how TRIM5 degrades HIV capsids. This paper is potentially important because it suggests an alternative mechanism where substrate (CEP192) multimerization is not required to trigger degradation. This is interesting, although it remains unproven whether the TRIM37-dependent degradation of CEP192 monomers in the cytoplasm suggested here is physiologically important.

This paper will be of interest to the centrosome/mitosis field and to a more general ubiquitination/protein degradation audience.
My expertise is in centrosome and cell cycle biology.

September 26, 2026

RE: JCB Manuscript #202608031T

Jeffrey Woodruff
The University of Texas Southwestern Medical Center

Dear Dr. Woodruff,

Thank you for submitting your revised manuscript entitled "TRIM37 recognizes a bipartite degron to ubiquitinate centrosome substrates." The manuscript was re-reviewed by two of the original reviewers from Review Commons. Reviewer #1 was not available to re-review. We would be happy to publish your paper in JCB pending final revisions necessary to meet our formatting guidelines (see details below).

To avoid unnecessary delays in the acceptance and publication of your paper, please read the following information carefully.

A. MANUSCRIPT ORGANIZATION AND FORMATTING:

Full guidelines are available on our Instructions for Authors page, https://jcb.rupress.org/submission-guidelines#revised. **Submission of a paper that does not conform to JCB guidelines will delay the acceptance of your manuscript.**

1) Text limits: Character count for Articles is < 40,000, not including spaces. Count includes title page, abstract, introduction, results, discussion, and acknowledgments. Count does not include materials and methods, figure legends, references, tables, or supplemental legends.

2) Figure formatting: Articles may have up to 10 main text figures. Scale bars must be present on all microscopy images, including inset magnifications. Molecular weight or nucleic acid size markers must be included on all gel electrophoresis. Please add MW markers to figures 1D/G, 4D (ponceau), & S1D.

Also, please avoid pairing red and green for images and graphs to ensure legibility for color-blind readers. If red and green are paired for images, please ensure that the particular red and green hues used in micrographs are distinctive with any of the colorblind types. If not, please modify colors accordingly or provide separate images of the individual channels.

3) Statistical analysis: Error bars on graphic representations of numerical data must be clearly described in the figure legend. The number of independent data points (n) represented in a graph must be indicated in the legend. Please indicate whether 'n' refers to technical or biological replicates (i.e. number of analyzed cells, samples or animals, number of independent experiments). If independent experiments with multiple biological replicates have been performed, we recommend using distribution-reproducibility SuperPlots (please see Lord et al., JCB 2020) to better display the distribution of the entire dataset, and report statistics (such as means, error bars, and P values) that address the reproducibility of the findings.

Statistical methods should be explained in full in the materials and methods. For figures presenting pooled data the statistical measure should be defined in the figure legends. Please also be sure to indicate the statistical tests used in each of your experiments (both in the figure legend itself and in a separate methods section) as well as the parameters of the test (for example, if you ran a t-test, please indicate if it was one- or two-sided, etc.). Also, if you used parametric tests, please indicate if the data distribution was tested for normality (and if so, how). If not, you must state something to the effect that "Data distribution was assumed to be normal but this was not formally tested."

4) Materials and methods: Should be comprehensive and not simply reference a previous publication for details on how an experiment was performed. Please provide full descriptions (at least in brief) in the text for readers who may not have access to referenced manuscripts. The text should not refer to methods "...as previously described." Please also indicate the acquisition and quantification methods for immunoblotting/western blots.

5) For all cell lines, vectors, strains, constructs/cDNAs, etc. - all genetic material: please include database / vendor ID (e.g. Addgene, ATCC, etc.) or if unavailable, please briefly describe their basic genetic features, even if described in other published work or gifted to you by other investigators (and provide references where appropriate). Please be sure to provide the sequences for all of your oligos: primers, si/shRNA, RNAi, gRNAs, etc. in the materials and methods. You must also indicate in the methods the source, species, and catalog numbers/vendor identifiers (where appropriate) for all of your antibodies, including secondary. If antibodies are not commercial, please add a reference citation if possible.

6) Microscope image acquisition: The following information must be provided about the acquisition and processing of images:
a. Make and model of microscope
b. Type, magnification, and numerical aperture of the objective lenses

c. Temperature
d. Imaging medium
e. Fluorochromes
f. Camera make and model
g. Acquisition software
h. Any software used for image processing subsequent to data acquisition. Please include details and types of operations involved (e.g., type of deconvolution, 3D reconstitutions, surface or volume rendering, gamma adjustments, etc.).

7) References: There is no limit to the number of references cited in a manuscript. References should be cited parenthetically in the text by author and year of publication. Abbreviate the names of journals according to PubMed.

8) Supplemental materials: Articles may have up to 5 supplemental figures and 10 videos. Please also note that tables, like figures, should be provided as individual, editable files. A summary of all supplemental material should appear at the end of the Materials and methods section. Please include one brief sentence per item.

9) eTOC summary: A ~40-50 word summary that describes the context and significance of the findings for a general readership should be included on the title page. The statement should be written in the present tense and refer to the work in the third person. It should begin with "First author name(s) et al..." to match our preferred style.

10) Conflict of interest statement: JCB requires inclusion of a statement in the acknowledgements regarding competing financial interests. If no competing financial interests exist, please include the following statement: "The authors declare no competing financial interests." If competing interests are declared, please follow your statement of these competing interests with the following statement: "The authors declare no further competing financial interests."

11) A separate author contribution section is required following the Acknowledgments in all research manuscripts. All authors should be mentioned and designated by their first and middle initials and full surnames. We encourage use of the CRediT nomenclature (https://casrai.org/credit/).

12) ORCID IDs: ORCID IDs are unique identifiers allowing researchers to create a record of their various scholarly contributions in a single place. Please note that ORCID IDs are required for all authors. At resubmission of your final files, please be sure to provide your ORCID ID and those of all co-authors.

13) JCB requires authors to submit Source Data used to generate figures containing gels and Western blots with all revised manuscripts. This Source Data consists of fully uncropped and unprocessed images for each gel/blot displayed in the main and supplemental figures. For assays performed using capillary electrophoresis and/or immunoassay-based detection, authors should instead provide the electropherogram graph(s) for each experiment, plotting fluorescence/chemiluminescence intensity vs. molecular weight/size. Since your paper includes cropped gel and/or blot images, please be sure to provide one Source Data file for each figure gels, blots, and/or capillary electrophoresis assays along with your revised manuscript files. File names for Source Data figures should be alphanumeric without any spaces or special characters (i.e., SourceDataF#, where F# refers to the associated main figure number or SourceDataFS# for those associated with Supplementary figures). For traditional gels and blots, the lanes of the gels/blots should be labeled as they are in the associated figure, the place where cropping was applied should be marked (with a box), and molecular weight/size standards should be labeled wherever possible. For capillary electrophoresis assays, each trace in the graph should be color-coded and labeled to indicate which protein, gene, or sample is being measured (please try to avoid red/green combinations to accommodate our color-blind readers).

Source Data files will be directly linked to specific figures in the published article. Source Data Figures should be provided as individual PDF files (one file per figure). Authors should endeavor to retain a minimum resolution of 300 dpi or pixels per inch. Please review our instructions for export from Photoshop, Illustrator, and PowerPoint here: https://rupress.org/jcb/pages/submission-guidelines#revised.

14) Journal of Cell Biology now requires a data availability statement for all research article submissions. These statements will be published in the article directly above the Acknowledgments. The statement should address all data underlying the research presented in the manuscript. Please visit the JCB instructions for authors for guidelines and examples of statements at (https://rupress.org/jcb/pages/editorial-policies#data-availability-statement).

B. FINAL FILES:

Please upload the following materials to our online submission system. These items are required prior to acceptance. If you have any questions, contact JCB's Managing Editor, Lindsey Hollander (lhollander@rockefeller.edu).

-- An editable version of the final text (.DOC or .DOCX) is needed for copyediting (no PDFs).

-- High-resolution figure and MP4 video files: See our detailed guidelines for preparing your production-ready images, https://jcb.rupress.org/fig-vid-guidelines.

-- Cover images: If you have any striking images related to this story, we would be happy to consider them for inclusion on the journal cover. Submitted images may also be chosen for highlighting on the journal table of contents or JCB homepage carousel. Images should be uploaded as TIFF or EPS files and must be at least 300 dpi resolution.

It is JCB policy that if requested, original data images must be made available to the editors. Failure to provide original images upon request will result in unavoidable delays in publication. Please ensure that you have access to all original data images prior to final submission.

The license to publish form must be signed before your manuscript can be sent to production. A link to the license to publish form will be sent to the corresponding author only. Please take a moment to check your funder requirements before choosing the appropriate license.

Additionally, JCB encourages authors to submit a short video summary of their work. These videos are intended to convey the main messages of the study to a non-specialist, scientific audience. Think of them as an extended version of your abstract, or a short poster presentation. We encourage first authors to present the results to increase their visibility. The videos will be shared on social media to promote your work. For more detailed guidelines and tips on preparing your video, please visit https://rupress.org/jcb/pages/submission-guidelines#videoSummaries.

Thank you for your attention to these final processing requirements. Please revise and format the manuscript and upload materials within 14 days. If you need an extension for whatever reason, please let us know and we can work with you to determine a suitable revision period.

Please contact the journal office with any questions at cellbio@rockefeller.edu.

Thank you for this interesting contribution, we look forward to publishing your paper in Journal of Cell Biology.

Sincerely,

Anna Akhmanova, PhD
Monitoring Editor
Journal of Cell Biology

Dan Simon, PhD
Scientific Editor
Journal of Cell Biology

Reviewer #2 (Comments to the Authors (Required)):

The authors have made substantial efforts to address many of the reviewer's questions by performing additional experiments and incorporating new data into the revised manuscript. As a result, the overall quality of the manuscript has been significantly improved. I am therefore in favor of publication of this manuscript.

Reviewer #3 (Comments to the Authors (Required)):

In this revised manuscript Stachera et al., have thoroughly addressed the major concerns raised in my initial review and I enthusiastically support publication in The JCB.
