# Supplementary material for: TRIM37 recognizes a bipartite degron to ubiquitinate centrosome substrates
Source: J Cell Biol. 2026 Nov 10;226(1):e202608031. doi: 10.1083/jcb.202608031 (PMC13750308; doi:10.1083/jcb.202608031)
Supplement: Supplementary file 2 [file jcb_202608031_sourcedataf1.pdf]

A

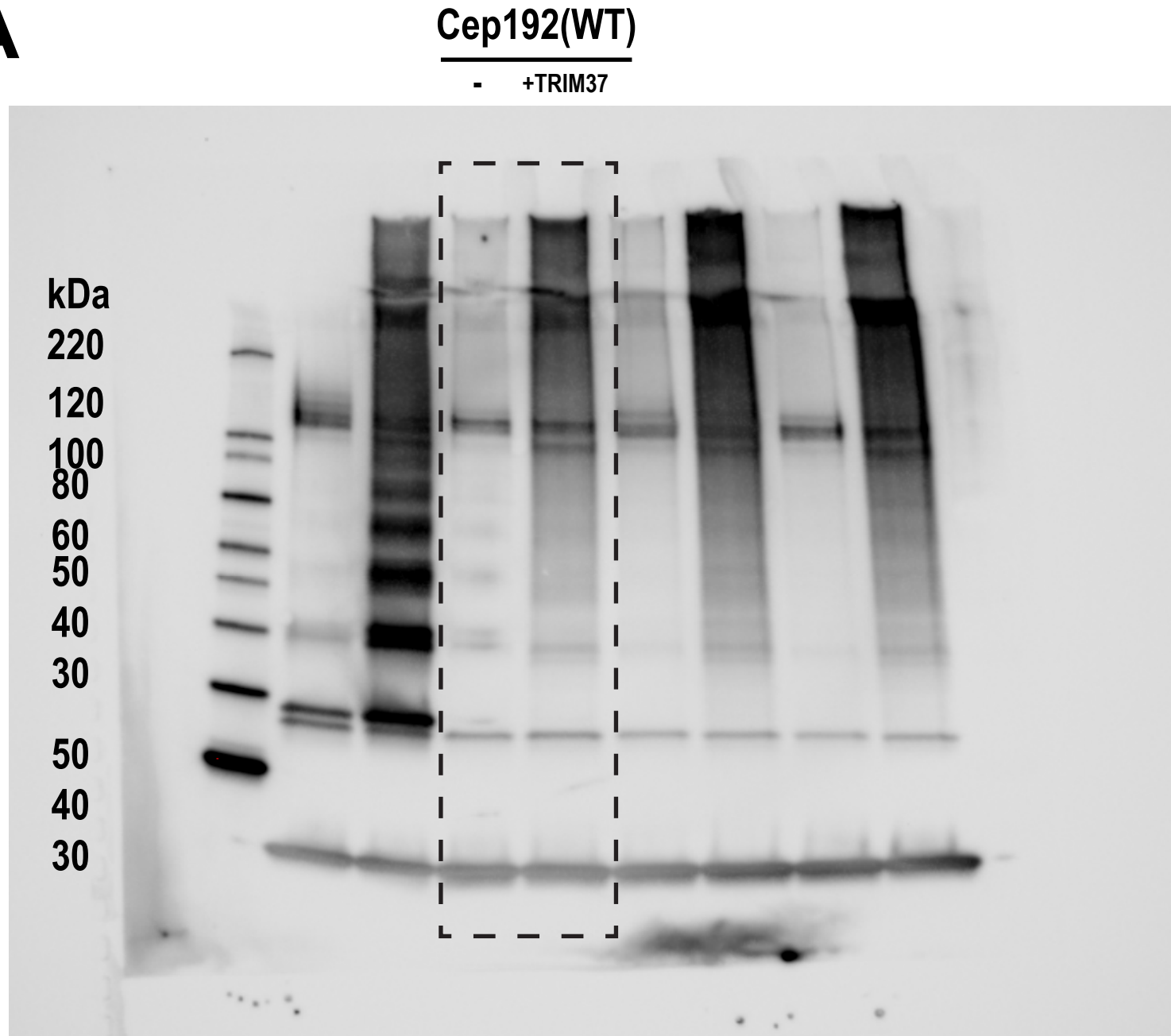
Cep192(WT)
-
+TRIM37
kDa
220
120
100
80
60
50
40
30
50
40
30

D

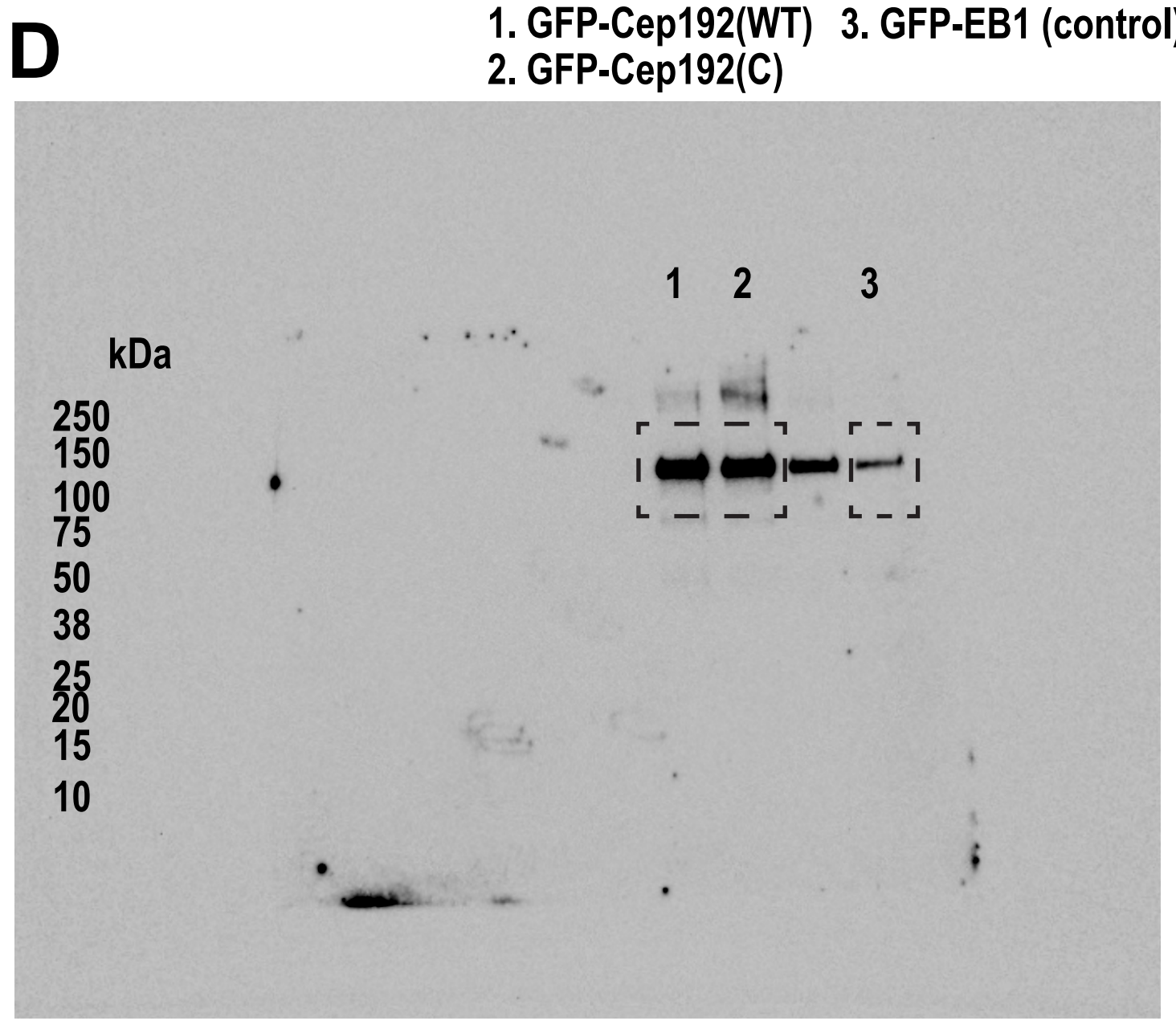
1. GFP-Cep192(WT)
2. GFP-Cep192(C)
3. GFP-EB1 (control)
1
2
3
kDa
250
150
100
75
50
38
25
20
15
10

E

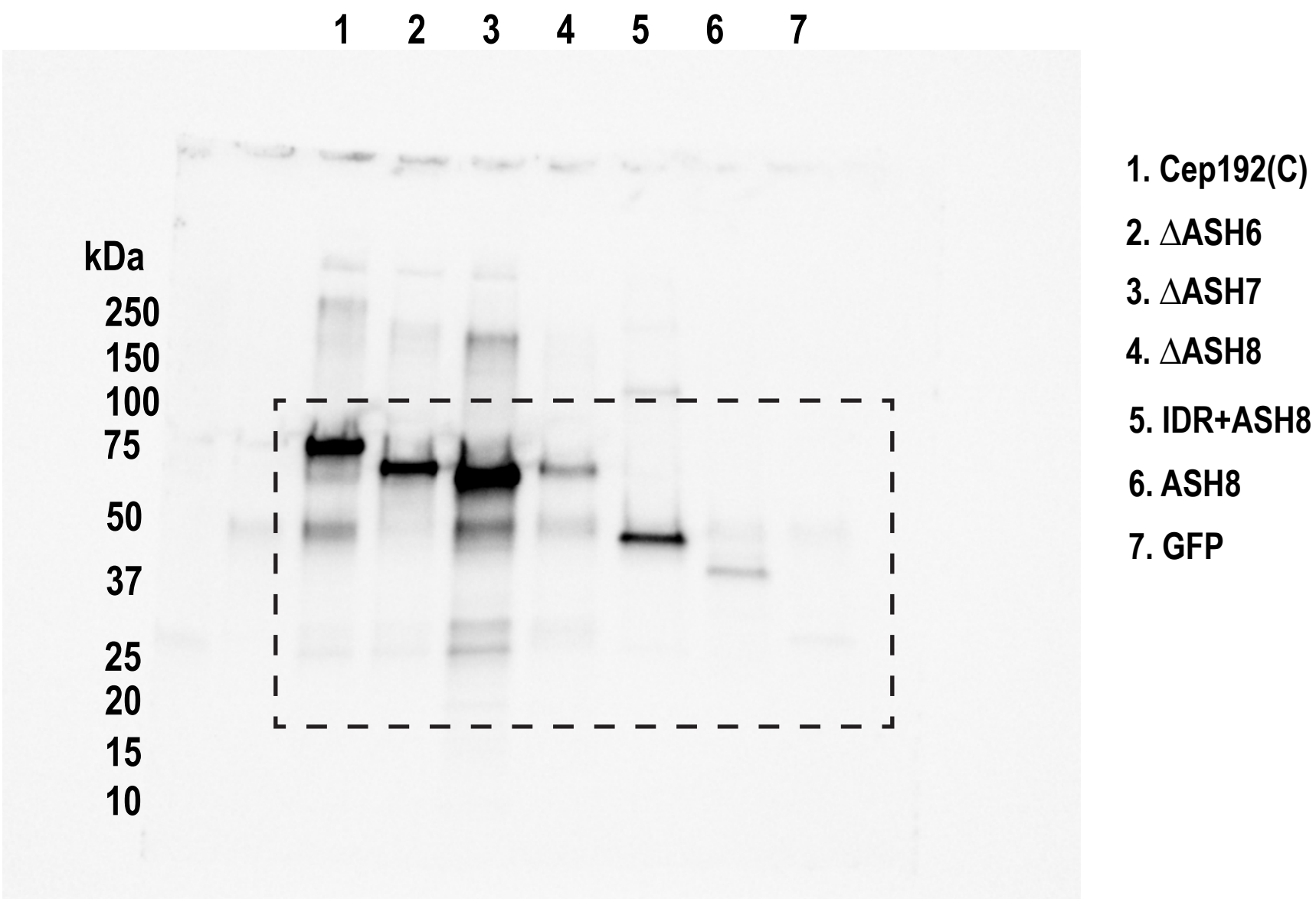
1
2
3
4
5
6
7
kDa
250
150
100
75
50
37
25
20
15
10
1. Cep192(C)
2. ∆ASH6
3. ∆ASH7
4. ∆ASH8
5. IDR+ASH8
6. ASH8
7. GFP

G

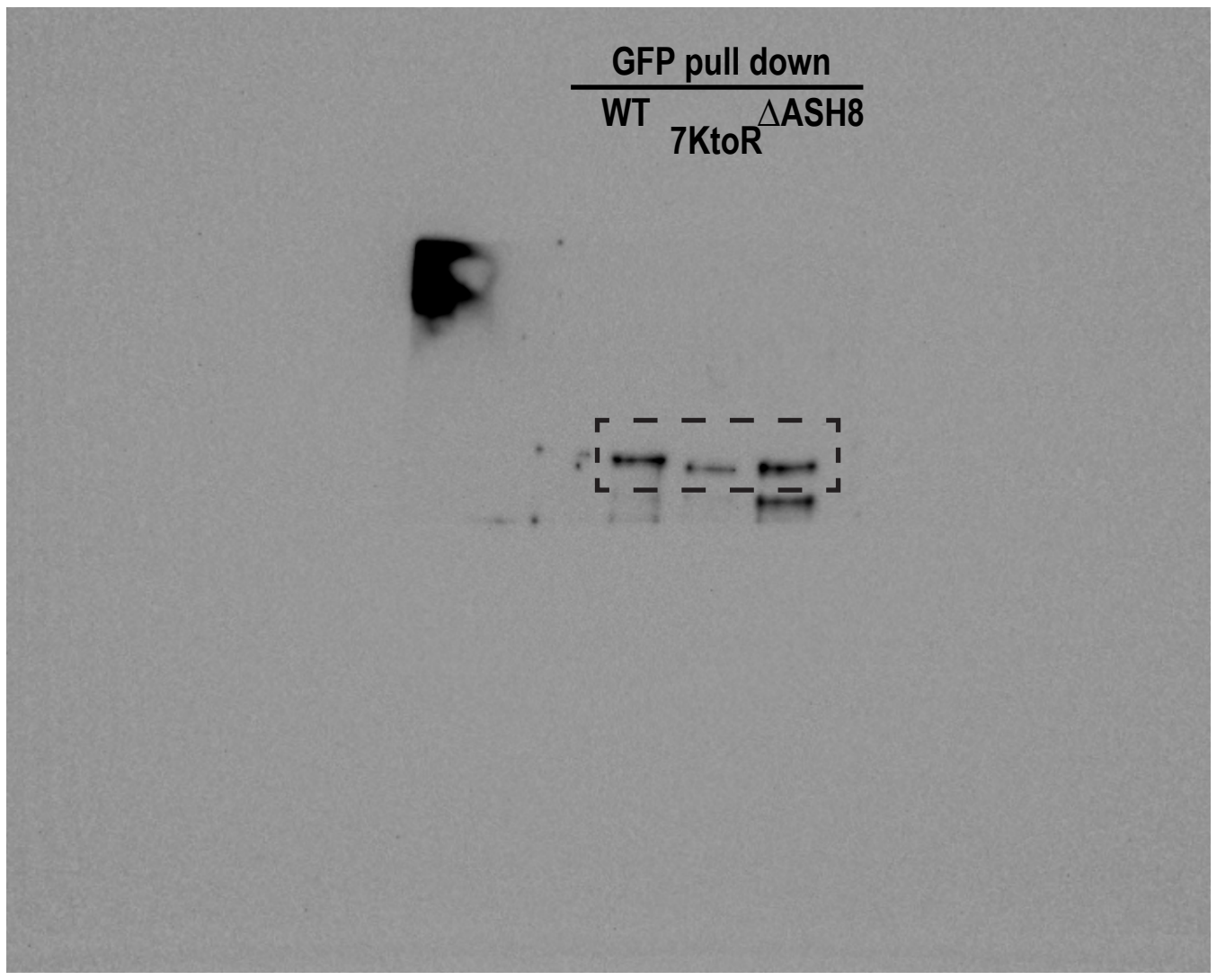
GFP pull down
WT
7KtoR
∆ASH8

H

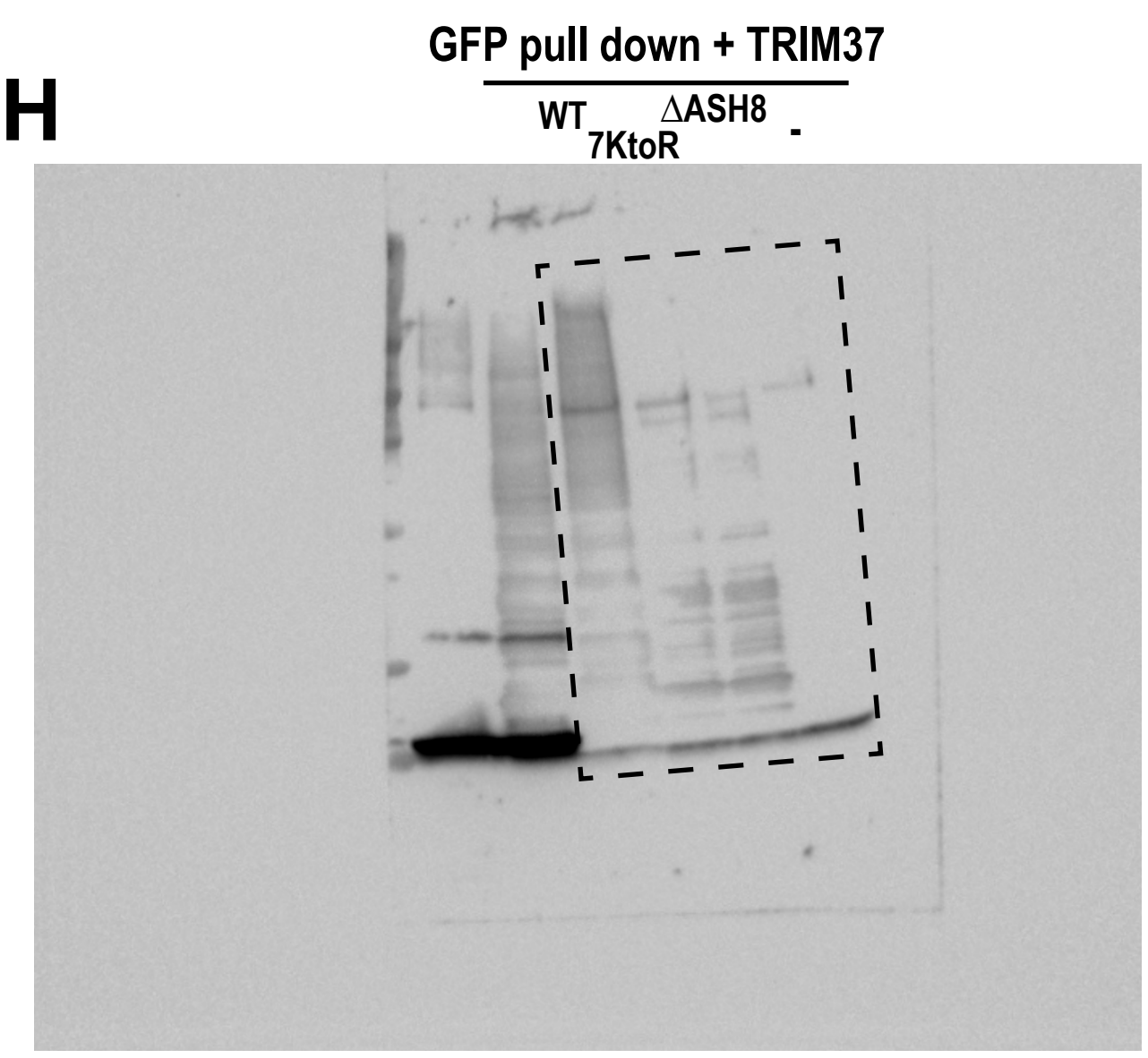
GFP pull down + TRIM37
WT
7KtoR
∆ASH8
-
