# Supplementary material for: TRIM37 recognizes a bipartite degron to ubiquitinate centrosome substrates
Source: J Cell Biol. 2026 Nov 10;226(1):e202608031. doi: 10.1083/jcb.202608031 (PMC13750308; doi:10.1083/jcb.202608031)
Supplement: Supplementary file 3 [file jcb_202608031_sourcedataf4.pdf]

D

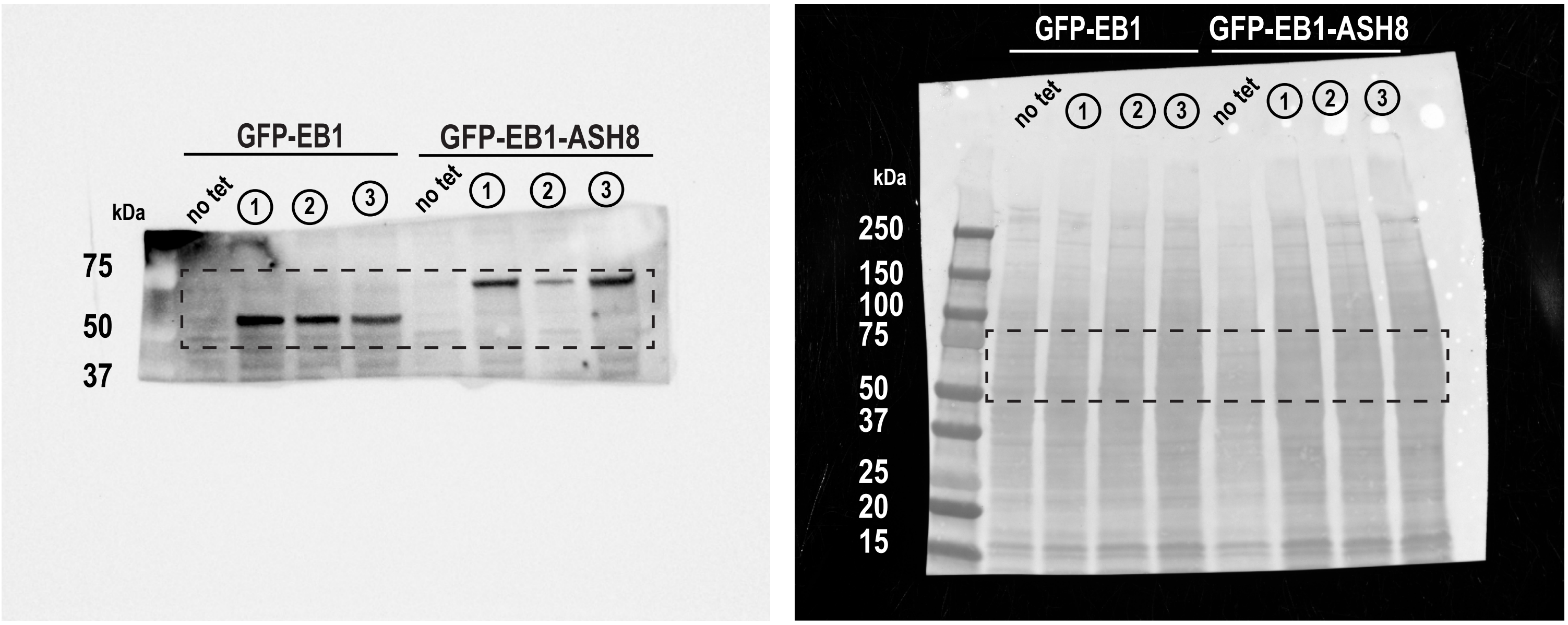

GFP-EB1
GFP-EB1-ASH8
kDa
no tet
1
2
3
no tet
1
2
3
75
50
37
GFP-EB1
GFP-EB1-ASH8
no tet
1
2
3
no tet
1
2
3
kDa
250
150
100
75
50
37
25
20
15
