# Supplementary material for: TRIM37 recognizes a bipartite degron to ubiquitinate centrosome substrates
Source: J Cell Biol. 2026 Nov 10;226(1):e202608031. doi: 10.1083/jcb.202608031 (PMC13750308; doi:10.1083/jcb.202608031)
Supplement: Supplementary file 4 [file jcb_202608031_sourcedatafs1.pdf]

B

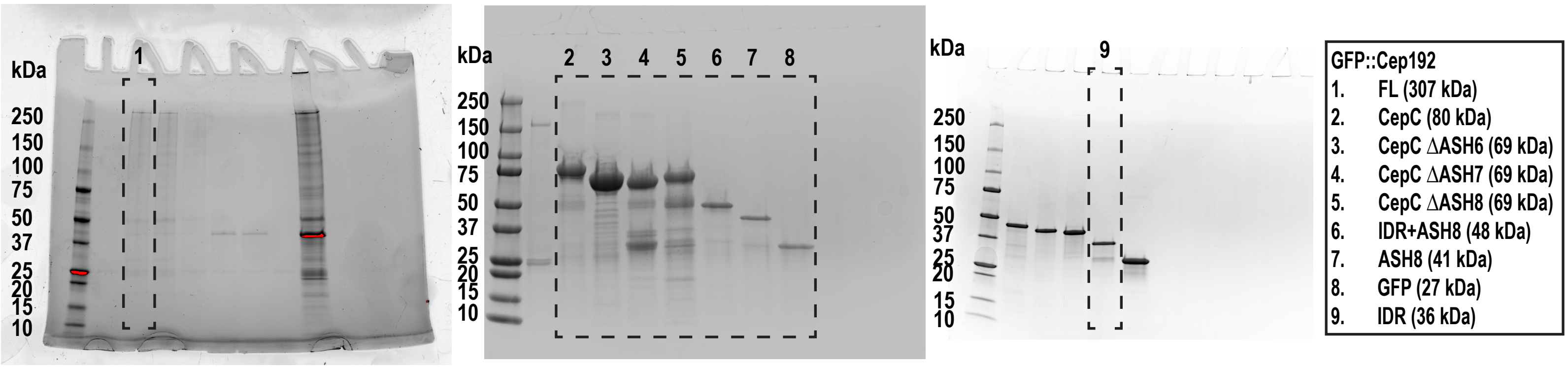
kDa
250
150
100
75
50
37
25
20
15
10
1
2
3
4
5
6
7
8
9
GFP::Cep192
1. FL (307 kDa)
2. CepC (80 kDa)
3. CepC ΔASH6 (69 kDa)
4. CepC ΔASH7 (69 kDa)
5. CepC ΔASH8 (69 kDa)
6. IDR+ASH8 (48 kDa)
7. ASH8 (41 kDa)
8. GFP (27 kDa)
9. IDR (36 kDa)

D

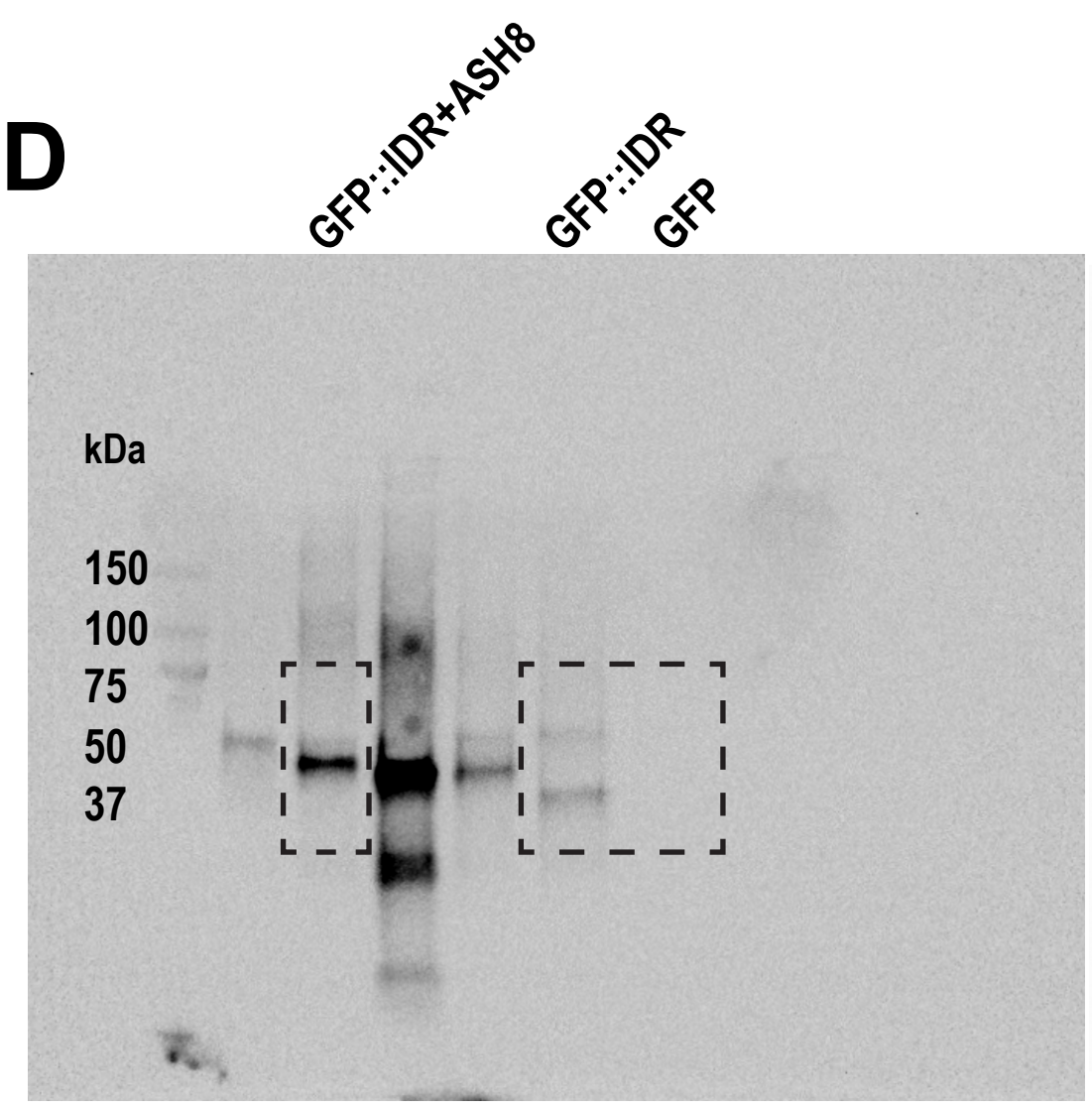
GFP::IDR+ASH8
GFP::IDR
GFP
kDa
150
100
75
50
37

F

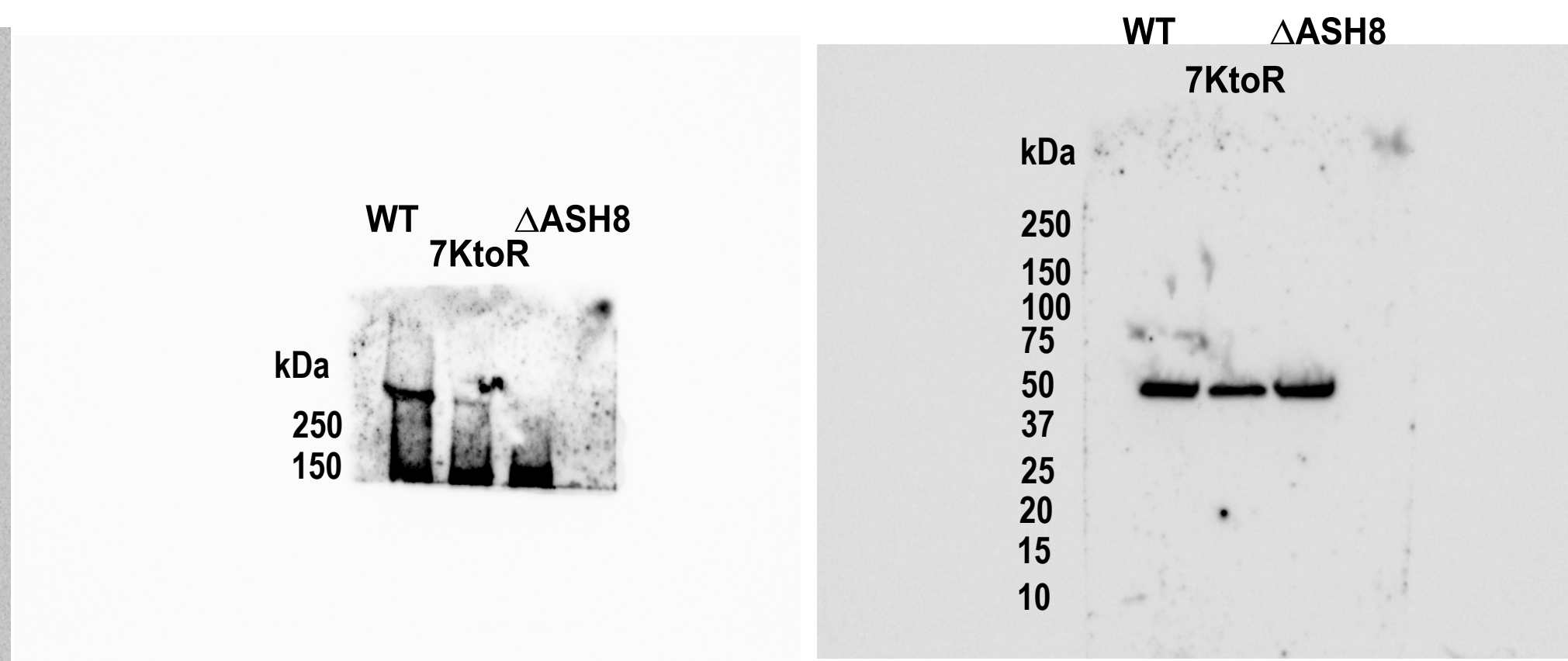
WT
7KtoR
ΔASH8
kDa
250
150
WT
7KtoR
ΔASH8
kDa
250
150
100
75
50
37
25
20
15
10

J

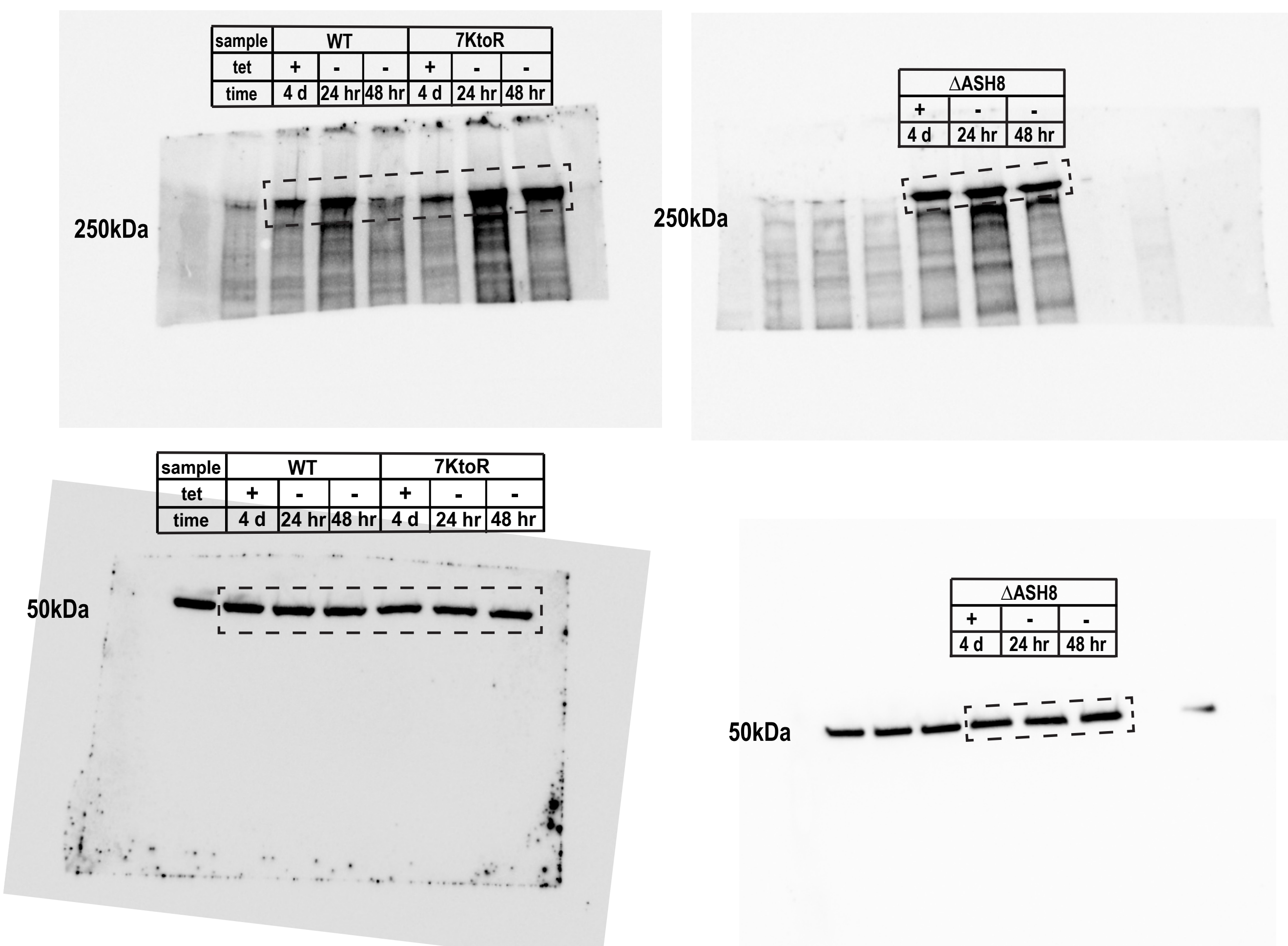
sample | WT | 7KtoR
tet | + | - | - | + | - | -
time | 4 d | 24 hr | 48 hr | 4 d | 24 hr | 48 hr
250kDa
ΔASH8
+ | - | -
4 d | 24 hr | 48 hr
250kDa
sample | WT | 7KtoR
tet | + | - | - | + | - | -
time | 4 d | 24 hr | 48 hr | 4 d | 24 hr | 48 hr
50kDa
ΔASH8
+ | - | -
4 d | 24 hr | 48 hr
50kDa
