# Supplementary material for: TRIM37 recognizes a bipartite degron to ubiquitinate centrosome substrates
Source: J Cell Biol. 2026 Nov 10;226(1):e202608031. doi: 10.1083/jcb.202608031 (PMC13750308; doi:10.1083/jcb.202608031)
Supplement: Supplementary file 5 [file jcb_202608031_sourcedatafs3.pdf]

A

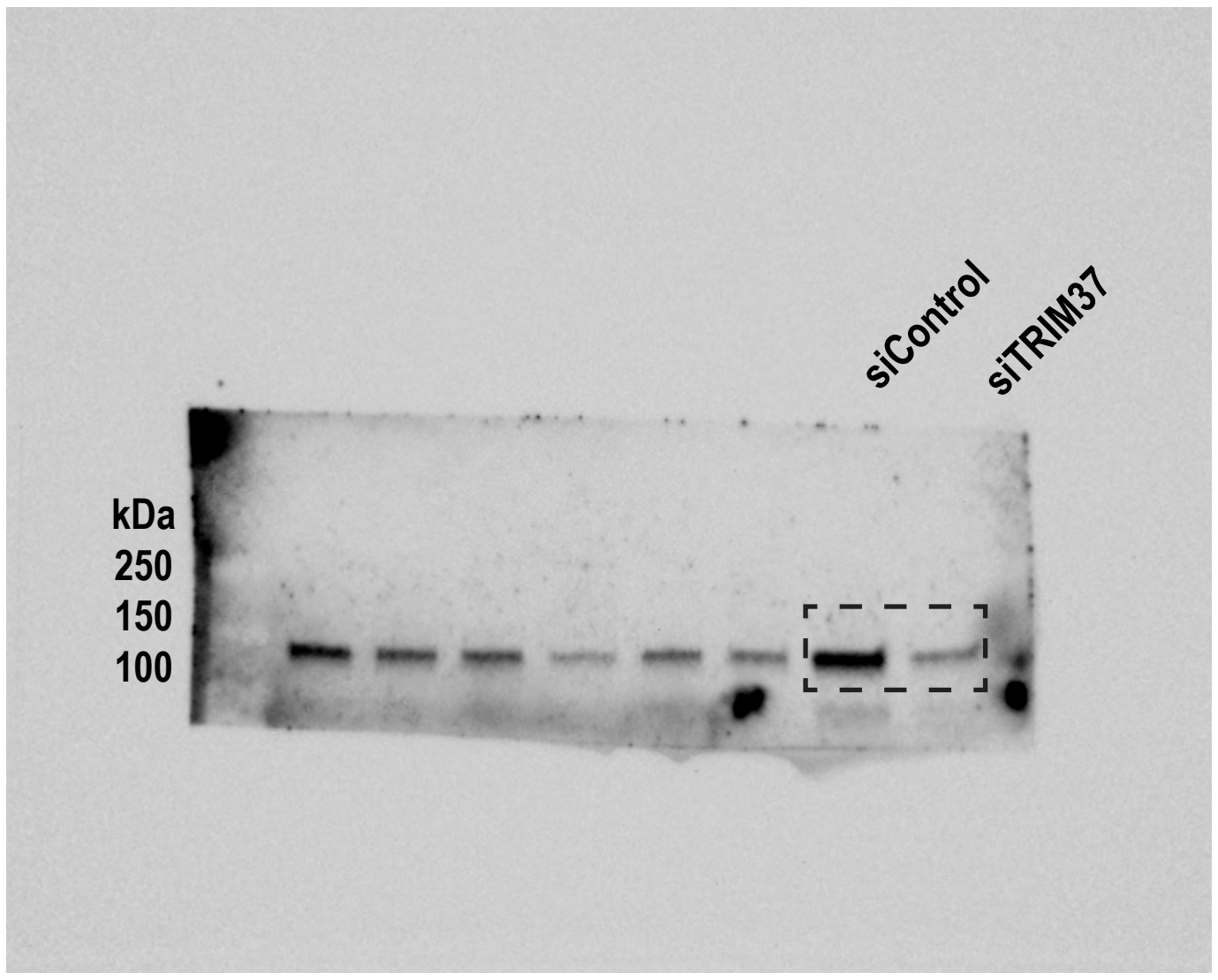

siControl
siTRIM37
kDa
250
150
100

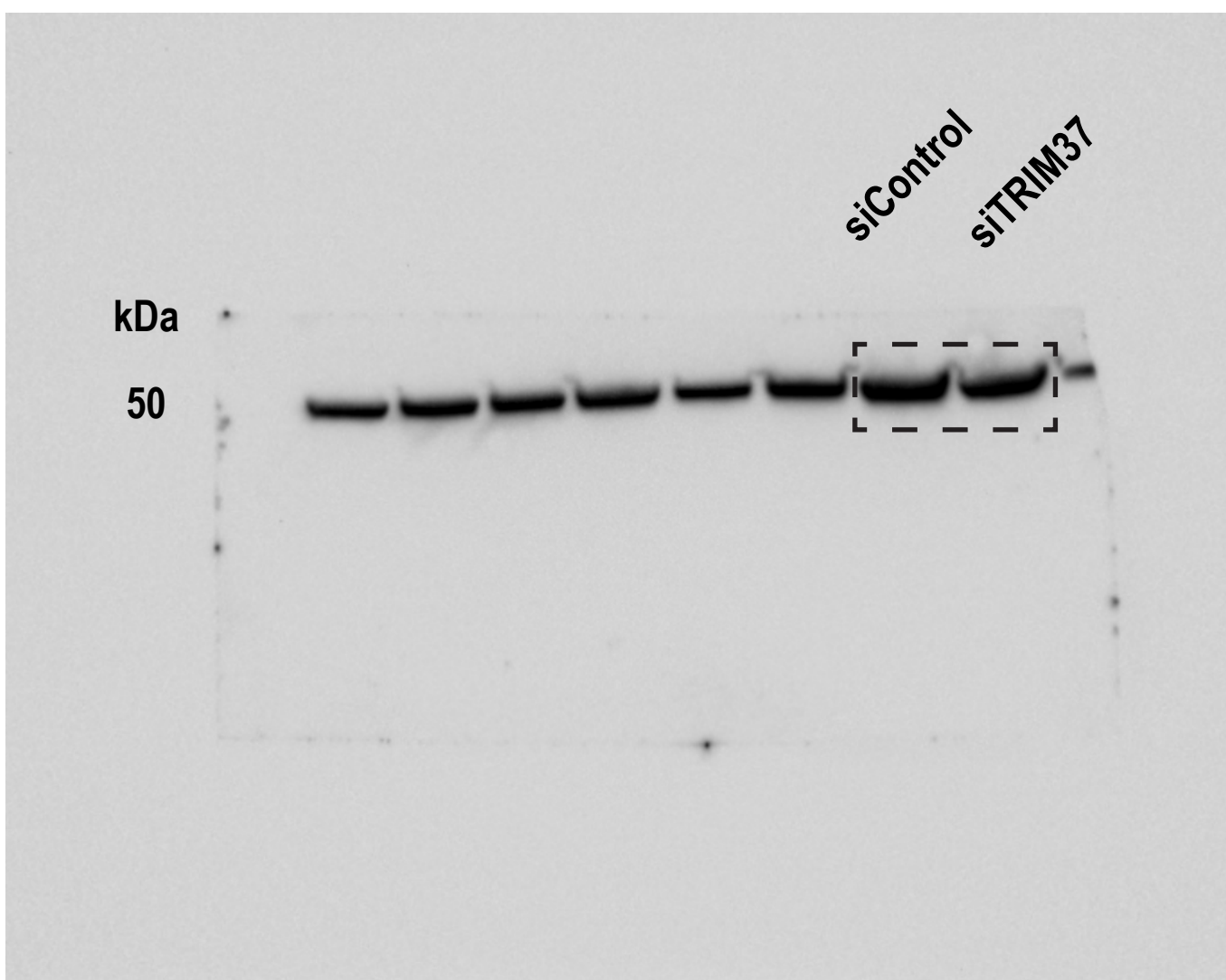

siControl
siTRIM37
kDa
50
