# Supplementary material for: TRIM37 recognizes a bipartite degron to ubiquitinate centrosome substrates
Source: J Cell Biol. 2026 Nov 10;226(1):e202608031. doi: 10.1083/jcb.202608031 (PMC13750308; doi:10.1083/jcb.202608031)

Shown in figure

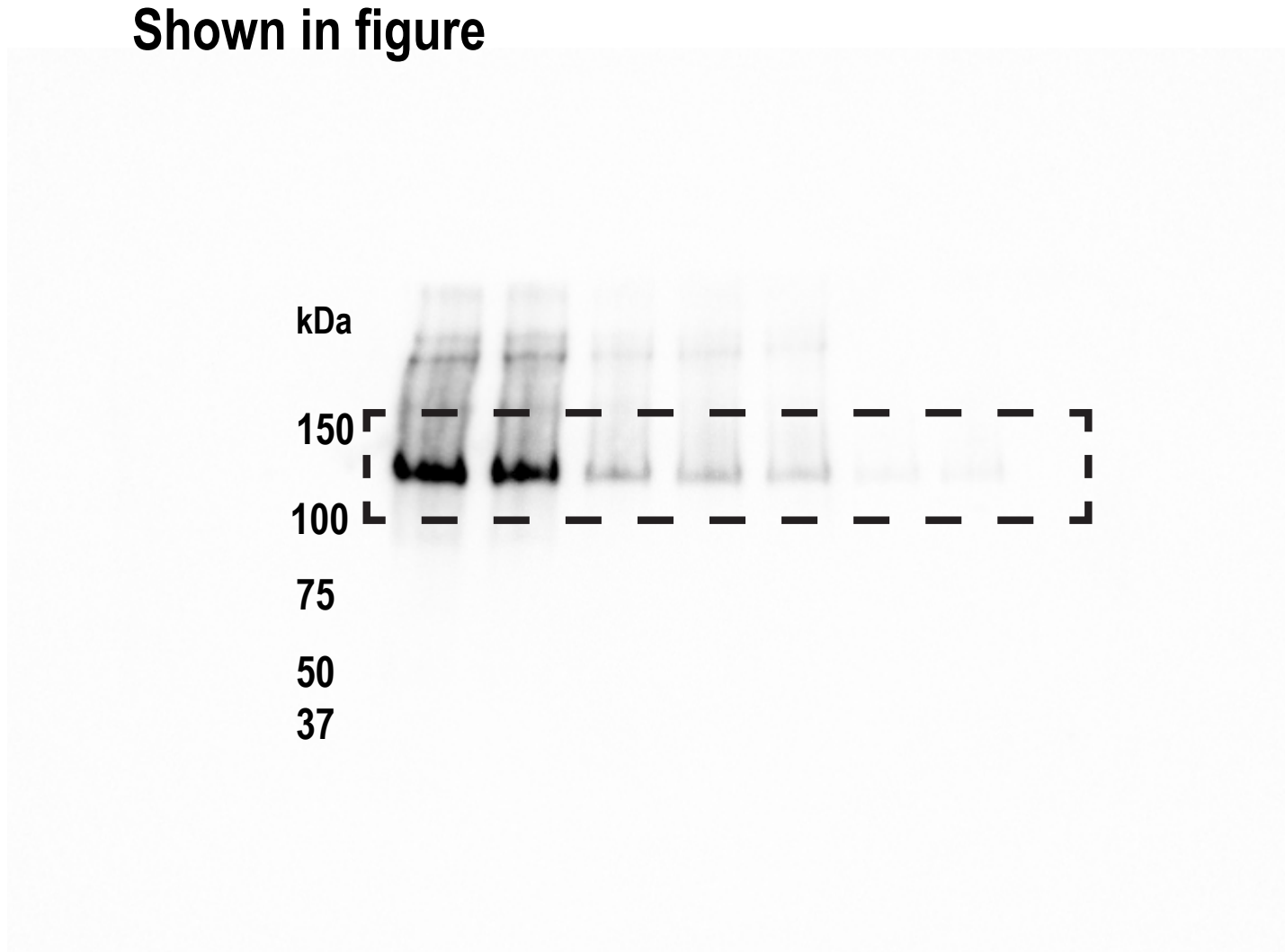

Enhanced contrast

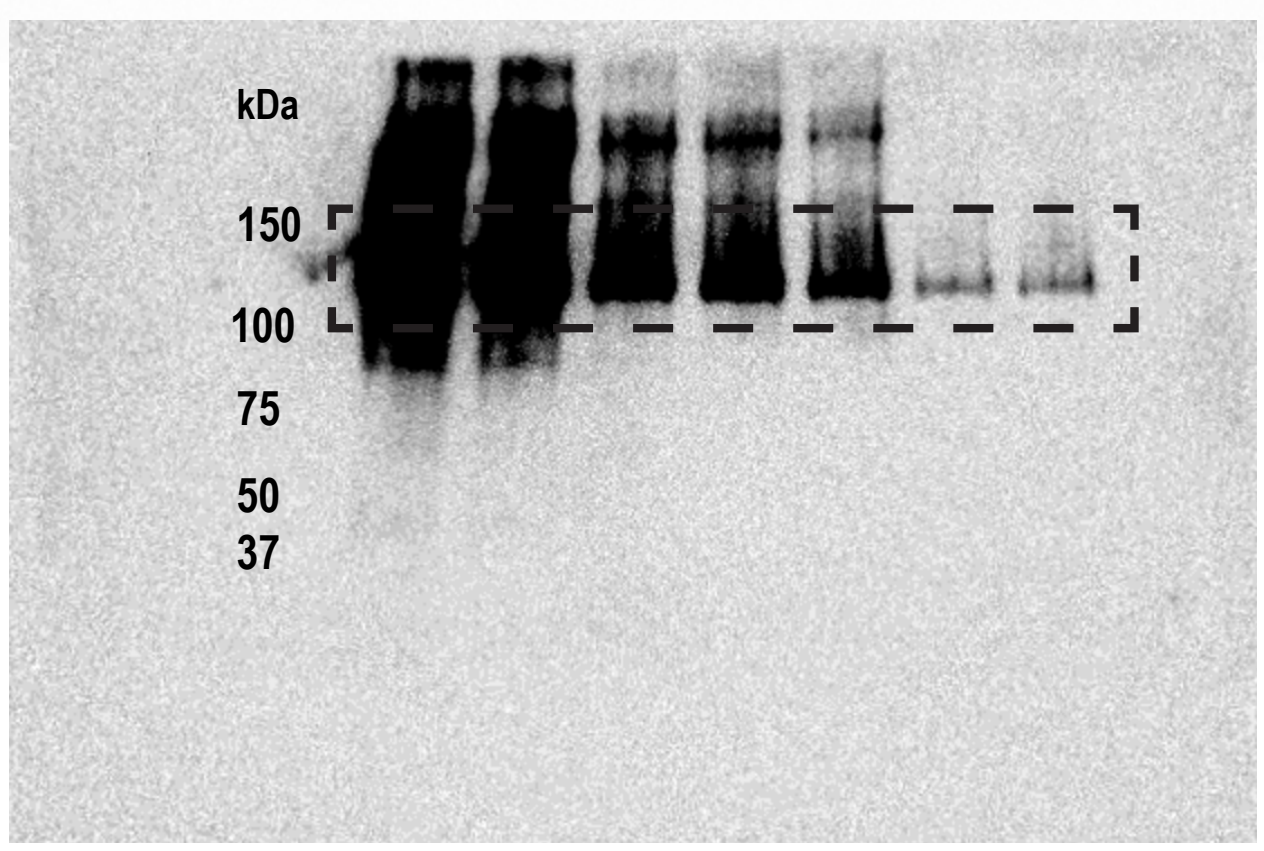

Supplement: Supplementary file 6 [file jcb_202608031_sourcedatafs4.pdf]
