# Supplementary material for: TRIM37 recognizes a bipartite degron to ubiquitinate centrosome substrates
Source: J Cell Biol. 2026 Nov 10;226(1):e202608031. doi: 10.1083/jcb.202608031 (PMC13750308; doi:10.1083/jcb.202608031)
Supplement: Supplementary file 7 [file jcb_202608031_tables1.docx]

**TABLE S1. Baculovirus constructs protein expression.**

| **protein** | **sequence** | **plasmid name** | **parent plasmid** | **N-term tag** | **C-term tag** |
| --- | --- | --- | --- | --- | --- |
| Cep192 | Full-length | JWV99 | pOCC29 | MBP-PreScission -**eGFP** | PreScission-6xHis |
| Cep192 C-term | a.a. 2070-2537 | JWV106 | pOCC27 | MBP-PreScission | **eGFP**-PreScission-6xHis |
| TRIM37 C18R | Full-length | JWV130 | pOCC27 | MBP-PreScission | **eGFP**-PreScission-6xHis |
