# Supplementary material for: TRIM37 recognizes a bipartite degron to ubiquitinate centrosome substrates
Source: J Cell Biol. 2026 Nov 10;226(1):e202608031. doi: 10.1083/jcb.202608031 (PMC13750308; doi:10.1083/jcb.202608031)
Supplement: Supplementary file 8 [file jcb_202608031_tables2.docx]

**TABLE S2. Bacterial constructs for protein expression.**

| **protein** | **plasmid name** | **parent plasmid** | **N-term tag** | **C-term tag** |
| --- | --- | --- | --- | --- |
| GFP | JWB66 | pET | 6xHis-GFP-TEV |  |
| EB1 | JWB3 | pTEMM1 | 6xHis-TEV | **eGFP** |
| Cep192 C-term | JWB194 | JWB66 | 6xHis-**eGFP**-TEV |  |
| Cep192 C-term ΔASH6 | JWB210 | JWB66 | 6xHis-**eGFP**-TEV |  |
| Cep192 C-term ΔASH7 | JWB206 | JWB66 | 6xHis-**eGFP**-TEV |  |
| Cep192 C-term ΔASH8 | JWB208 | JWB66 | 6xHis-**eGFP**-TEV |  |
| IDR + ASH8 | JWB223 | JWB66 | 6xHis-**eGFP**-TEV |  |
| ASH8 | JWB225 | JWB66 | 6xHis-**eGFP**-TEV |  |
