# Supplementary material for: TRIM37 recognizes a bipartite degron to ubiquitinate centrosome substrates
Source: J Cell Biol. 2026 Nov 10;226(1):e202608031. doi: 10.1083/jcb.202608031 (PMC13750308; doi:10.1083/jcb.202608031)
Supplement: Supplementary file 9 [file jcb_202608031_tables3.docx]

**TABLE S3. Constructs for protein expression using TREx Flp-In™ system in DLD-1 human colon cancer cells.**

| Protein expressed | mutation | Cell line name | parent plasmid |
| --- | --- | --- | --- |
| GFP::Cep192 | WT | JWM25 | JWB179 |
| GFP::Cep192 | 7KtoR | JWM24 | JWB180 |
| GFP::Cep192 | no ASH8 (1-2437aa) | JWM28 | JWB224 |
| GFP::EB1 | WT | JWM 29 | JWB237 |
| GFP::EB1::ASH8 | IDR_ASH8 | JWM 30 | JWB230 |
| GFP | WT | JWM 32 | JWB259 |
| GFP::IDR_ASH8 | IDR_ASH8 | JWM 31 | JWB260 |
